# Monomer and dimer pathways of earth-abundant manganese tricarbonyl pre-catalysts for CO_2_ reduction studied by time-resolved IR spectroscopy

**DOI:** 10.1039/d5cp03590b

**Published:** 2025-12-23

**Authors:** Luka Tatarashvili, Noah von Fellenberg, Kerstin Oppelt, Peter Hamm

**Affiliations:** a Department of Chemistry, University of Zürich Zürich Switzerland peter.hamm@chem.chem.uzh

## Abstract

The activation mechanism of Mn-based molecular catalysts is reported in a three-component system (photosensitizer, electron donor, catalyst), investigated by time-resolved infrared spectroscopy. In total four complexes were studied that are derived from Mn(2,2′-bipyridine)(CO)_3_Br by varying substituents on the ligand, which impose steric constraints or modulate electronic properties. Thereby, ligand effects on catalyst activation pathways are systematically assessed. A unified feature across all systems is that the intermediate after one-electron reduction and subsequent Br^−^ dissociation, *i.e.*, Mn^0^(L)(CO)_3_, possesses a more positive reduction potential than the parent complex, leading to its rapid second reduction. This step outcompetes dimerization of Mn^0^ radicals, which instead proceeds through a symproportionation between the two-electron reduced species and the parent complex. However, when dimer formation is sterically hindered, two Mn^0^ species arise instead. The final process is the re-oxidation of the reduced intermediates by either hydrogen evolution or regeneration of the electron donor. Although CO_2_ conversion was not the focus of this work, the elucidated pathways clarify how competing re-oxidation channels can limit reduction efficiency or alter product selectivity. These mechanistic insights provide a foundation for rational strategies to control the selectivity and amplify the desired catalytic reactions.

## Abstract 2

The activation mechanism of Mn-based molecular catalysts is reported in a three-component system (photosensitizer, electron donor, catalyst), investigated by time-resolved infrared spectroscopy.

## Graphical abstract

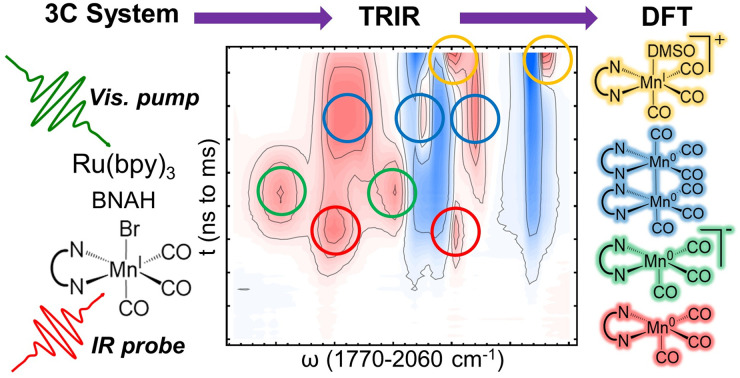

## 1 Introduction

Concerns about the accumulation of greenhouse gases have garnered increasing attention due to compelling evidence of global warming.^1^ Atmospheric CO_2_ has been identified as the major controlling factor for the greenhouse effect,^2^ but it is also a renewable and abundant C1 building block. Reduction of the atmospheric carbon dioxide through its utilization for producing valuable chemicals is desired.^3^ However, the latter is not an easy task, since CO_2_ is an inherently stable molecule and its reduction comes at a high thermodynamic cost.^4^ One-electron reduction of CO_2_ requires 1.9 eV of free energy at standard ambient temperature and pressure in aqueous solutions. Proton-coupled multi-electron transfer processes such as CO_2_+ 2H^+^+ 2e^−^ → CO + H_2_O significantly lower this energy requirement to 0.53 eV.^5^

Transition metal complexes have been applied since the 1980s as catalysts for these reactions.^6^ Originally they were made using rare metals such as Re,^7–9^ Ru,^10,11^ Ir and Rh.^12,13^ However, in 2011, Chardon-Noblat, Deronzier and coworkers reported the first application of a Manganese-based catalyst^14^ - Mn(2,2′-bipyridine)(CO)_3_Br - for CO_2_ reduction. This initiated a series of studies that focused on utilizing complexes of more abundant metals for this purpose. The results have been summarized in recent reviews.^4,15–29^

Improving catalyst design requires a deep understanding of underlying mechanisms involved in the catalytic cycle. While various electrochemical and spectroscopic investigations on Mn-based CO_2_ reduction catalysts have been conducted, time-resolved studies are much rarer. Nevertheless, a number of transient infrared studies have demonstrated the power of this technique in elucidating the reaction dynamics of Mn carbonyl complexes involved in other catalytic processes, such as C–H bond functionalization.^30–34^ As for the catalytic CO_2_ reduction, a notable kinetic study of a Mn-based catalyst (Mn(4,4′-ditertbutyl-2,2′-bipyridine)(CO)_3_ Br) was conducted by Grills *et al.*^35^ using pulse radiolysis to generate solvated electrons and measure IR absorption changes on nanosecond to millisecond timescales. Subsequently, the Wasielewski group investigated a modified Mn(2,2′-bipyridine)(CO)_3_Br complex covalently linked to a chromophore enabling observation of the first reduction step with enhanced temporal resolution.^36^ Very recently, Hammarström and colleagues have done extensive work towards identifying bifurcating pathways leading to CO or formate products by stopped-flow FTIR spectroscopy, using chemical reductant mixing to generate active catalyst and observe subsequent CO_2_ reduction.^37^

In parallel, the first steady-state photo-chemical experiments using the Mn-based catalyst were performed by Ishitani and colleagues,^38^ where they have utilized the originally reported catalyst - Mn(2,2′-bipyridine)(CO)_3_Br, Ru(dmb)_3_^2+^ as photosensitizer, and 1-benzyl-1,4-dihydronicotinamide as sacrificial electron donor (a typical 3-component system) in the presence of triethanolamine in dimethylformamide. Irradiation with 480 nm light selectively excited the photosensitizer, while avoiding the direct absorption of the catalyst which is known to act as a highly effective photoCORM (photo-induced CO-releasing molecule).^39–42^ Grills *et al.* noted that laser flash photolysis experiments could be carried out under the intermolecular electron transfer conditions established by Ishitani,^38^ but cautioned that introducing a more complex chemical system might lead to a mechanism different from that reported in their own study.^35^

In the present work we address this question and investigate the typical 3-component photocatalytic system using time-resolved infrared spectroscopy. Among the prior IR studies, only a few have successfully detected CO_2_-related intermediates.^37,43^ However, none have done so within a full three-component system, which poses a significant experimental and mechanistic challenge. Given this complexity, it is essential to first disentangle the intrinsic activation pathways before introducing CO_2_. Accordingly, the present study is carried out in the absence of CO_2_ to establish a clear mechanistic baseline, independent of substrate-specific reactivity. Because the presence of CO_2_ can fundamentally alter the reaction network, isolating the inherent steps of catalyst activation represents a necessary precursor to understanding the complete reduction cycle. The insights gained here thus provide a crucial foundation for future time-resolved studies under catalytic conditions and will guide the selection of suitable parameters for exploring the full CO_2_ reduction mechanism.

**Fig. 1:**
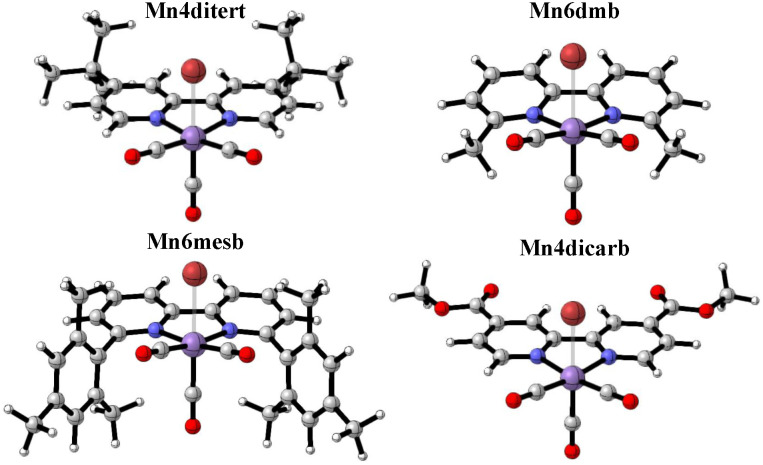
The four catalysts used in this study.

To that end, four different catalysts were selected to systematically probe steric and electronic influences on their activation pathways: Mn(4,4′-ditertbutyl-2,2′-bipyridine)(CO)_3_Br (hereafter Mn4ditert) previously employed by Grills *et al.* in their mechanistic investigation, Mn(6,6′-dimethyl-2,2′-bipyridine)(CO)_3_Br (hereafter Mn6dmb), that unlike the three other complexes exhibits a characteristic bending of the bipyridine ring system, Mn(6,6′-dimesityl-2,2′-bipyridine)(CO)_3_Br (hereafter Mn6mesb) utilized by Kubiak and colleagues, whose bulky ligands suppress the catalyst dimerization,^44^ and Mn(4,4′-dimethylester-2,2′-bipyridine)(CO)_3_Br^41^ (hereafter Mn4dicarb), which provides comparative insight about the reaction pathway in the presence of electron-withdrawing, instead of electron-donating, substituents on the bipyridine ligand. The four catalysts are shown in Fig. 1 below.

## 2. Results and discussion

A typical chemical system for homogeneous photo-catalysis contains at least two additional components besides the molecular catalyst: a photosensitizer (PS), which harvests light and converts it into chemical potential, and an electron donor. In photochemical reduction, the electron donor either reduces the excited photosensitizer (reductive quenching) or recovers the oxidized PS˙^+^ (oxidative quenching pathway).^45^ This study utilizes Ru(bpy)_3_(PF_6_)_2_ as PS, which is very commonly used for this role,^46^ whereas the electrons are provided by 1-benzyl-1,4-dihydronicotinamide (hereafter BNAH), an analogue compound of NAD(P)H in biological systems.^47^ The synthesis of the complexes is described in SI, Section 1.^14,42,44,48,49^ The PS was excited using 532 nm wavelength pump pulse to decrease the probability of direct absorption by the catalysts (see Fig. S6 in SI), which would lead to a number of photochemical side products (see Section S3.1 in SI).^40–42,50^ In fact, due to the inherent photo-instability of the Mn(α-diimine)(CO)_3_Br-type complexes, the number of steady-state experiments that can provide mechanistic insight into such complex three-component systems is rather limited. The strength of time-resolved spectroscopy is hereby underlined, as the use of pulsed actinic light is essential for preventing absorption process by reaction intermediates, which absorb green light far more strongly than the parent complex.^51^ The species formed after the actinic pulse were tracked by IR probe pulses in the region of Mn carbonyl vibrations around ∼1800–2000 cm^−1^ (see SI, Section S3, for details).^52,53^

### 2.1. Mn4ditert and Mn6dmb: reaction pathway of dimerizing catalysts

The most commonly described mechanism to reach the catalytically active state of a Mn tricarbonyl CO_2_ reduction catalyst, *i.e.*, the two-electron reduced complex, proceeds *via* one-electron reduction and subsequent halide loss, followed by dimer formation and then further reduction as shown in eqn (1) to (4) (L = α-diimine ligand).^14,35,54^ Rarely, two-electron reduction before dimer formation by comproportionation (see eqn (5) and (6)) has also been reported.^55,56^

1 Mn^I^(L)(CO)_3_Br + e^−^ ⇌ [Mn^I^(L˙^−^)(CO)_3_Br]^−^

2 [Mn^I^(L˙^−^)(CO)_3_Br]^−^ ⇌ Mn^0^(L)(CO)_3_ + Br^−^

3 Mn^0^(L)(CO)_3_ + Mn^0^(L)(CO)_3_ ⇌ [Mn^0^(L)(CO)_3_]_2_

4 [Mn^0^(L)(CO)_3_]_2_ + 2e^−^ ⇌ 2[Mn^0^(L˙^−^)(CO)_3_]^−^

5 Mn^0^(L)(CO)_3_+ e^−^ ⇌ [Mn^0^(L˙^−^)(CO)_3_]^−^

6 [Mn^0^(L˙^−^)(CO)_3_]^−^ + Mn^I^(L)(CO)_3_Br ⇌ [Mn^0^(L)(CO)_3_]_2_ + Br^−^

Mn4ditert and Mn6dmb systems serve as reference cases in this study due to their almost identical chemical behavior, whereas the other two complexes (Mn6mesb and Mn4dicarb) deviate from the standard reaction pathway through modifications on the bipyridine ligand. The following section is divided into parts focusing on reactions before and after dimer formation.

#### 2.1.1. Reduction, Br^−^ loss, second reduction: an electrochemical-chemical-electrochemical mechanism

**Fig. 2:**
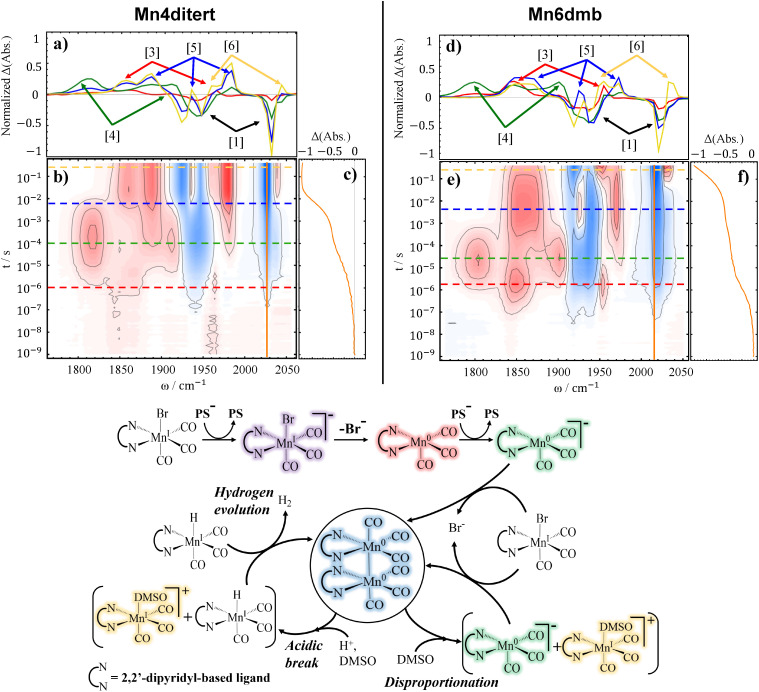
TRIR difference spectra accompanied by spectral cuts at selected delays and time trace(s) for 2 catalysts - Mn4ditert (a–c) and Mn6dmb (d–f) - at 10 mM concentration with 10 mM PS, and 50 mM BNAH in DMSO. The reaction pathway is shown below, with structure colors matching the spectral cuts that represent those intermediates. Additionally, the numbers in brackets assist in following the chronology of the major reaction pathway and point to the relevant bands in the horizontal traces.

TRIR data obtained for the reference complexes, Mn4ditert and Mn6dmb systems, suggests very similar reaction pathways (see Fig. 2). In the 2D contour plots of this work (for example Fig. 2b and e), blue color is used to show a negative change in absorption whereas red represents a positive one. That is, red bands belong to new species that are formed over the course of reaction, while blue bands, hereafter referred to as bleaches, indicate depletion of the initial catalyst. The parent complex of each catalyst belongs to the *C*_s_ point group and its 3 carbonyls generate 3 normal modes: 2A′ (lowest and highest frequency modes) and 1A″ mode (in between the two). Of the three modes, two with lower frequencies are close to each other and are not well-resolved.

For a systematic analysis of the spectra we have employed the so-called “lifetime distribution analysis”, a largely model-independent technique that facilitates extraction of time constants (*via* dynamical content plots) and subtle spectral variations that could otherwise go unnoticed (*via* lifetime density maps).^57–62^ A detailed description of the method is provided in the SI, Section S4.

The assignment of the observed spectral features to specific chemical intermediates was primarily guided by DFT-calculated vibrational frequencies and intensities,^63–70^ with assistance from prior literature reports whenever possible. For more detailed description of the computational methods the reader is referred to Section S5 in the SI.

The first reduction is observed on the ∼1 µs timescale (as deduced from the dynamical content in Fig. S14 in SI) and is assigned to the appearance of Mn^0^(L)(CO)_3_ (L = bpy-based ligand, bands labelled [3] in Fig. 2 - red structure), which manifests simultaneously alongside the bleaches (bands labelled [1] in Fig. 2) of the parent complex. The spectrum of Mn^0^(L)(CO)_3_ is in good agreement with the previous reports.^35,36^ We do not have spectroscopic evidence for the very first intermediate along the catalyst reduction pathway - [Mn^I^(L˙^−^)(CO)_3_Br]^−^ (violet structure in Fig. 2) - that would have carbonyl vibrations near ∼1900 cm^−1^ (2 bands) and 2000 cm^−1^, according to accompanying DFT calculations, see Table 1. This is because the diffusion-limited reaction between the reduced PS and the catalyst is slower than the loss of the Br^−^ ligand.

**Table 1:** DFT-calculated frequencies of the carbonyl modes of the discussed species for each complex. The modes are listed in the order from lowest to highest frequency. Modes in italic are for the theoretically possible but unobserved species. A more detailed version which includes intensities and free energies can be found in SI

Chemical species	Mn6dmb	Mn4ditert	Mn6mesb	Mn4dicarb
Mn^I^(L)(CO)_3_Br	1927/1936/2019	1931/1935/2023	1925/1942/2016	1940/1943/2026
[Mn^I^(L˙^−^)(CO)_3_Br]^−^	1900/1908/1997	1905/1908/2001	1900/1915/1996	1914/1917/2008
Mn^0^(L)(CO)_3_	1851/1856/1955	1860/1861/1961	1856/1862/1952	1903/1905/1978
[Mn^0^(L˙^−^)(CO)_3_]^−^	1803/1816/1887	1817/1823/1900	1827/1831/1900	1862/1862/1940
[Mn^0^(L)(CO)_3_]_2_	1862/1866/1881	1868/1873/1881	N/A	1900/1908/1917
	1888/1920/1969	1888/1925/1973		1920/1958/1995
[Mn^I^(L)(CO)_3_(DMSO)]^+^	1940/1954/2038	1948/1951/2042	1943/1952/2032	1955/1959/2044
Mn^I^(L)(CO)_3_H	1892/1893/1987	1893/1900/1992	1894/1899/1984	1906/1913/1995
Mer-Mn^I^(L)(CO)_3_Br	1924/1946/2041	1921/1949/2042	1931/1942/2035	1932/1962/2046

The next spectral changes are assigned to rapid accumulation of the doubly-reduced catalyst ([Mn^0^(L˙^−^)(CO)_3_]^−^), which is the active species for CO_2_ reduction, appearing with ∼13 µs time constant for Mn4ditert and ∼6 µs for Mn6dmb. This is evidenced by the bands near 1800 cm^−1^ and 1900 cm^−1^, that align well with the literature-reported values of the doubly-reduced complex^44^ (bands labelled [4] in Fig. 2, green structure). Considering the photon flux, beam spot size, and the extinction coefficient of the PS at 532 nm, one can estimate a ∼2 mM concentration of the excited PS (PS*). Even if all the PS* were reduced by BNAH, the resulting electron supply would still be insufficient to reduce all of the initial complex twice. The oxidized BNAH (BNAH˙^+^) is not reductive enough in this regard either (one-electron reduction of Mn(bpy)(CO)_3_ Br is reported at −1.56 V,^14^ and the deprotonated form of BNAH˙^+^, BNA˙, has potential of −1.38^71,72^*versus* Ag/Ag^+^ in Acetonitrile). The most plausible explanation is that the reduction potentials of Mn^I^ (L)(CO)_3_ Br and PS˙^−^ are about the same so the first reduction step is in fact incomplete and that Mn^0^(L)(CO)_3_ has a more positive reduction potential than the parent complex (Mn^I^(L)(CO)_3_Br), which leads to the preferential reduction of Mn^0^(L)(CO)_3_ by the PS˙^−^ as shown in eqn (5). The described reaction pathway is a typical example of an ECE mechanism, *i.e.*, electron transfer, chemical reaction, electron transfer.^73^

#### 2.1.2. Dimerization and proton attack

In the next reaction step, the doubly-reduced catalyst attacks the remaining parent complex and forms a dimer, see eqn (6). This is evidenced by additional bleaching of the resting state species in the millisecond window (see Fig. 2c and f). The dimer modes arise with a time constant of ∼6 ms for Mn4ditert and ∼0.5 ms for Mn6dmb (see Section S4 in SI). Notably this second reduction kinetically competes with the dimerization process shown in eqn (3). This rate constant for dimerization is significantly slower than the one reported by Grills *et al.*^35^ This discrepancy can be explained by the higher number of available electrons in their experiment, causing that dimer formation proceeds *via*eqn (3) instead of eqn (6).^55,56^

The dimer has *C*_2_ point group symmetry and 6 normal modes (3A and 3B symmetries). Of these six modes, two can be resolved at ∼1925 (B) and ∼1975 cm^−1^ (A). The band at ∼1925 cm^−1^ is predicted by DFT to be the most intense one (see Section S5 in SI), but appears around the same intensity as the rest due to the partial cancellation from overlapping broad bleach centered at ∼1940 cm^−1^. The broad bands located between ∼1840–1880 cm^−1^ originate from the remaining four CO modes that are superimposed, two pairs of A and B symmetries, respectively. The observed dimer spectrum is consistent with prior studies.^35,36^

**Fig. 3:**
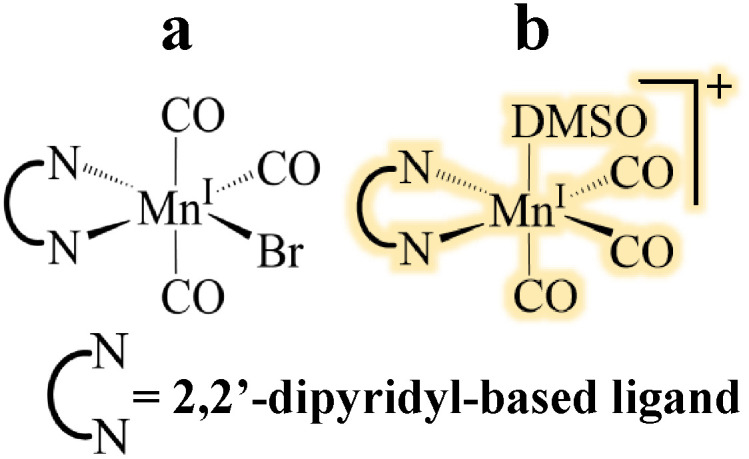
Two possible options to assign bands labelled [6] in Fig. 2a, the most likely option is highlighted.

The decay of the dimer bands is observed for both Mn4ditert and Mn6dmb samples coupled to the appearance of positive features at ∼1955 cm^−1^ and ∼2040 cm^−1^ (bands labelled [6] in Fig. 2a and d). For Mn6dmb, the new bands are temporally well separated from the dimer modes, emerging with a time constant of ∼200 ms, whereas for Mn4ditert they appear nearly simultaneously, *i.e.*, at ∼6 ms. These frequencies likely originate from a Mn^I^ species. Possible candidates include the meridional isomer of the catalyst and the solvent-coordinated *fac*-isomer (a and b, respectively in Fig. 3). The DFT calculated frequencies of the two candidates (see Table 1) indicate that both the meridional isomer - *mer*-Mn^I^ (L)(CO)_3_ Br - and the solvent-coordinated species can be responsible for these new peaks. However, since in the *mer*-isomer two of the carbonyls face opposite directions, the totally symmetric stretch mode becomes very weak, which does not match the observed intensity profile. Hence it is more reasonable to assign the bands to [Mn^I^(L)(CO)_3_(DMSO)]^+^ (yellow structure in Fig. 2 and 3). The solvato-complex is most plausibly generated through proton attack on the dimer, a reaction that would necessarily proceed *via* the hydride intermediate. However, direct observation of the hydride is challenging, because it can form H_2_ and dimerize with another metal hydride (see Fig. 2, hydrogen evolution pathway). Although Mn^I^(L)(CO)_3_H is not detected during the dimer re-oxidation, its spectroscopic evidence emerges in another process, which is discussed later on in Subsection 2.1.3.

The source of the proton is BNAH˙^+^, which is quite acidic^45^ and in the absence of a base the protons could attack the electron-rich Mn^0^–Mn^0^ dimer which would produce the Mn^I^ (L)(CO)_3_H and [Mn^I^ (L)(CO)_3_]^+^ according to:

7 [Mn^0^(L)(CO)_3_]_2_ + BNAH˙^+^ → Mn^I^(L)(CO)_3_H + BNA˙ + [Mn^I^(L)(CO)_3_]^+^

The latter would re-coordinate Br^−^ or a solvent molecule to either form the original complex or a solvent-coordinated one ([Mn^I^(L)(CO)_3_(DMSO)]^+^), whereas the BNA˙ can either dimerize with another radical (eqn (8)) or reduce BNAH˙^+^ to BNAH (eqn (9)).8
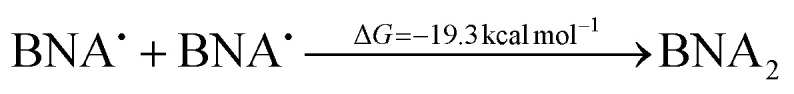
9
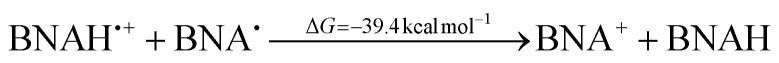


At first glance, it is surprising that the solvato-complex (yellow structure in Fig. 2) forms, rather than regeneration of the initial catalyst. Given the long timescale of the final process, one might expect establishment of equilibrium conditions that favor Br^−^ re-coordination over solvent binding. However, the experimental results suggest that solvent binding is thermodynamically preferred. This was confirmed by FTIR control experiments (Section S2 in SI). The solvolysis of Mn–Br bond for similar complexes has been described in literature before,^74,75^ however, it is a slow reaction (an hour timescale). Without the proton attack on Mn^0^–Mn^0^ dimer - which results in the re-oxidation of Mn^0^ to Mn^I^ - the solvato-complex would not be detected in this temporal window.

None of the reaction steps discussed so far account for the pronounced additional depletion of the parent complex observed on the same timescale as the formation of [Mn^I^(L)(CO)_3_(DMSO)]^+^ (see Fig. 2f), a phenomenon we only observed for the Mn6dmb system. To account for this, we propose a dimer disproportionation into [Mn^0^(6dmbpy˙^−^)(CO)_3_]^−^ (*i.e.*, the two-electron reduced catalyst, green structure in Fig. 2) and resting state [Mn^I^(6dmbpy)(CO)_3_]^+^ that binds DMSO (see eqn (10)).

10 [Mn^0^(L)(CO)_3_]_2_ + DMSO → [Mn^0^(L˙^−^)(CO)_3_]^−^ + [Mn^I^(L)(CO)_3_(DMSO)]^+^

The [Mn^0^(6dmbpy˙^−^)(CO)_3_]^−^ could reduce the resting state catalyst again, repeatedly regenerate the dimer, and ultimately lead to more solvent-coordinated Mn^I^ species. These reactions are also included in the Fig. 2 (disproportionation pathway), and are quite similar to the ones reported for analogous Ru-based system in the literature.^76–78^ Although this is putative, we propose that the bending of the bpy ring system in Mn6dmb favors the disproportionation reaction and this is the reason why it is not observed in the case of Mn4ditert experiments. DFT results show that Mn–Mn bond distance is 2.98 Å for Mn4ditert dimer, whereas for the Mn6dmb one it is 3.12 Å.

#### 2.1.3. Suppressing the acidic break pathway with base

**Fig. 4:**
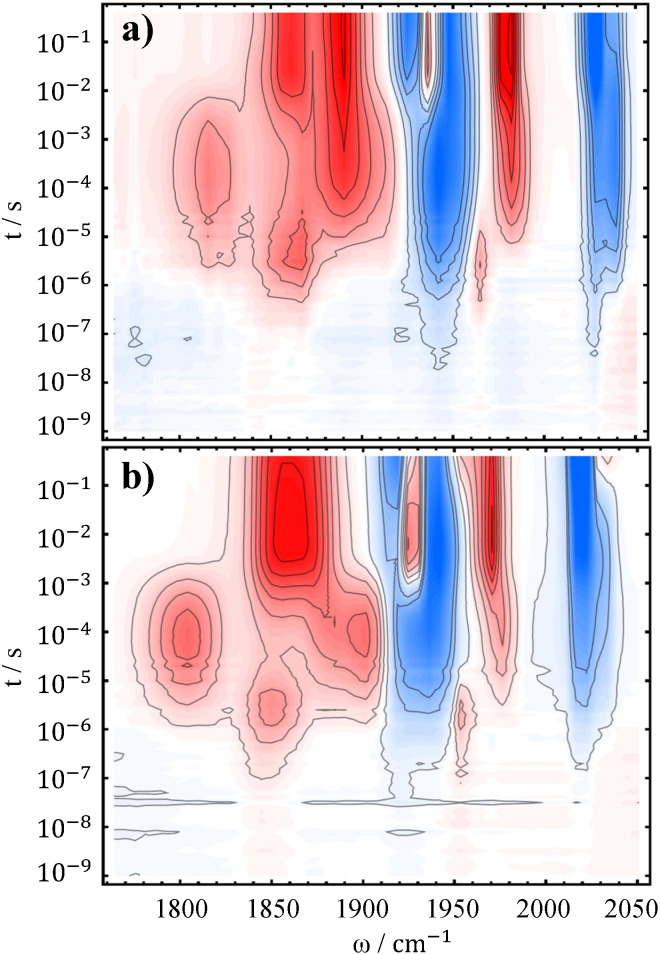
TRIR difference spectra of 10 mM Mn4ditert (a) and Mn6dmb (b), with 10 mM PS, 50 mM BNAH and additional 1 M TEOA.

Ishitani and colleagues^38^ utilized TEOA as a base in their experiments in order to suppress charge recombination of the reduced PS with the oxidized electron donor. To mimic their conditions, we added 1 M TEOA to our standard samples. The resulting TRIR spectra are shown in Fig. 4. Even though the added TEOA (p*K*_a_ of 7.77 in water^79^) did not completely suppress protonation of the dimer (eqn (7)), its net effect was unambiguous. Firstly, addition of base efficiently prevented the back-electron transfer from reduced PS to oxidized BNAH, by deprotonating the latter, which in turn improves the quantum yield of the overall reduction and thus the signal size in the difference spectra. Secondly, due to the reduction of the acidic proton concentration the dimer decays significantly slower, as evidenced by its persisting bands even at the end of the measured temporal window. For this reason, it can be regarded as the major product of the reaction under the basic conditions. Another re-oxidation pathway depleting the dimer can be electron back-transfer to PS, which has more positive potential than the dimer, but we don't have spectroscopic evidence of this reaction.

Addition of the base enhanced the detectability of another process that had previously gone unnoticed. Specifically, a new band appears near 1980 cm^−1^, most apparent for the Mn6dmb spectrum (Fig. 4b), while its lower frequency counterpart is obscured by the ∼1900 cm^−1^ mode of the [Mn^0^(L˙^−^)(CO)_3_]^−^. We have considered two reasonable options to explain this feature, which is discussed in greater detail in SI, Section S4.1. First, a different conformation of the dimer that forms earlier and then rearranges to the one we see in the millisecond regime. However, only one, pseudo-eclipsed conformer with respect to equatorial carbonyls, fits the observed intensity profile of the dimer. Second, considering the fact that the presence of the corresponding species is affected by base addition, it should be related to a metal hydride complex. We hence propose that Mn^0^(L)(CO)_3_ can receive hydrogen atom transfer from BNAH˙^+^ and form Mn^I^(L)(CO)_3_H, which would recombine with another hydride and lead to formation of the Mn^0^–Mn^0^ dimer:11
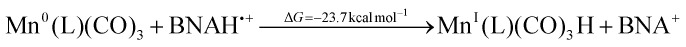
12
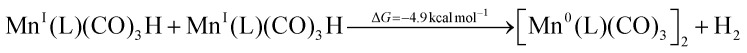


The metal hydride decomposition into a dimer has been reported to take place even in the solid state.^80^ DFT calculations suggest that the hydride has carbonyl bands near ∼1890 cm^−1^ (2 modes) and ∼1990 cm^−1^ (see Table 1), which closely matches the observed spectrum, as well as experimental reports on similar hydrides.^80,81^

### 2.2. Non-dimerizing activation pathway

**Fig. 5:**
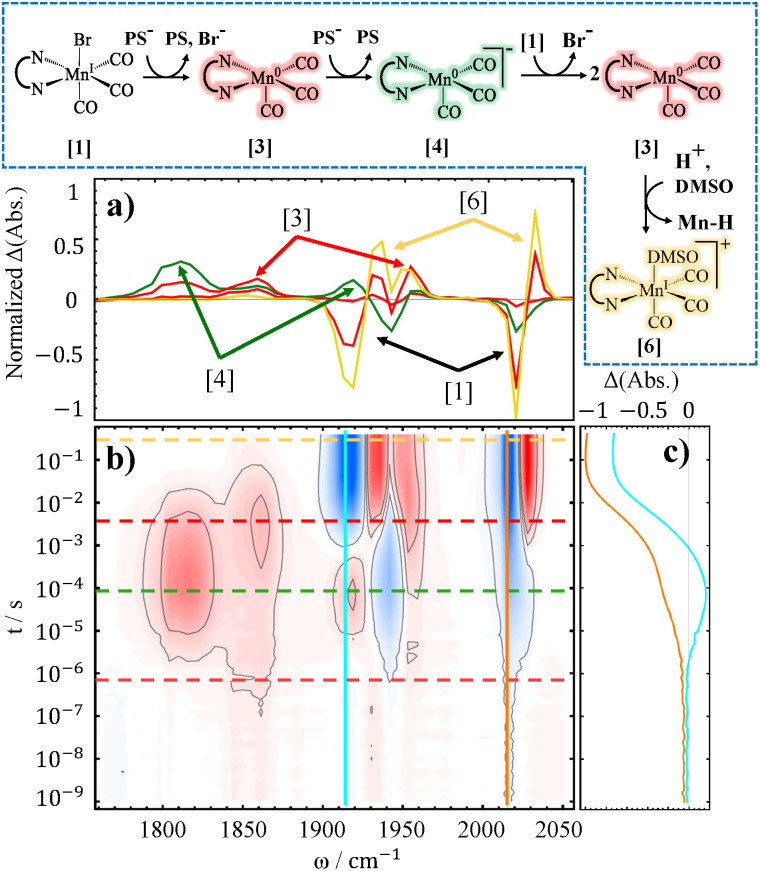
TRIR difference spectra of 10 mM Mn6mesb, 10 mM PS, and 50 mM BNAH in DMSO (b), accompanied by spectral cuts at selected delays (a), and time trace(s) (c). The reaction pathway is shown on top of the figure, boxed by blue dashed line. Mn-H denotes the hydride complex.

In order to understand the activation pathway of a non-dimerizing catalyst we investigated Mn(6,6′-dimesityl-2,2′-bipyridine)(CO)_3_Br, designed by Kubiak and coworkers^44,82^ utilizing the ligand first made by Schmittel *et al.*^49^ The TRIR spectrum of the compound is shown in Fig. 5b. The first observable intermediate, Mn^0^(L)(CO)_3_ (compound [3] in Fig. 5a), is not clearly resolved since its higher frequency band overlaps with 1940 cm^−1^ bleach and cancels out. Similarly, the higher frequency band of the doubly-reduced complex, [Mn^0^(L)(CO)_3_]^−^ (compound [4] in Fig. 5a), partially coincides with the same bleach. Nevertheless, Mn^0^(L)(CO)_3_ can be seen with a faint positive feature at 1955 cm^−1^ evolving at ∼1 µs timescale, whereas [Mn^0^(L)(CO)_3_]^−^ is clearly observed at 1810 cm^−1^ and 1919 cm^−1^ at ∼32 µs. Hence, up to the formation of the doubly-reduced catalyst the reaction mechanism for Mn6mesb is similar to the one described above for Mn4ditert and Mn6dmb.

The mesitylene substituents in Mn6mesb efficiently prevent dimerization after the formation of [Mn^0^(L)(CO)_3_]^−^. Instead, the 2-electron reduced complex undergoes a comproportionation reaction with the parent complex to form Mn^0^(L)(CO)_3_:13
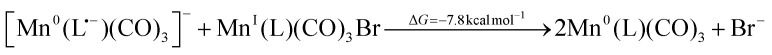


The equilibrium of this reaction seems to be on the side of the one-electron reduced intermediate since its population maximum arises later than that of the doubly reduced species, developing with a time constant of ∼130 µs. This outcome is predictable based on the DFT-calculated energy of −7.8 kcal mol^−1^ for the reaction (eqn (13)), that favors two Mn^0^(L)(CO)_3_ over [Mn^0^(L˙^−^)(CO)_3_]^−^ and Mn^I^(L)(CO)_3_Br. Another pathway depleting the doubly-reduced species could be a proton attack on the [Mn^0^(L˙^−^)(CO)_3_]^−^:14
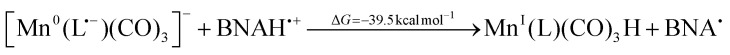


Moreover, the reverse reaction of two Mn^0^(L)(CO)_3_ radicals regenerating [Mn^0^(L˙^−^)(CO)_3_]^−^ and [Mn^I^(L)(CO)_3_]^+^ (reverse of eqn (13)) preferentially yields [Mn^I^(L)(CO)_3_(DMSO)]^+^ (compound [6] in Fig. 5a).

**Fig. 6:**
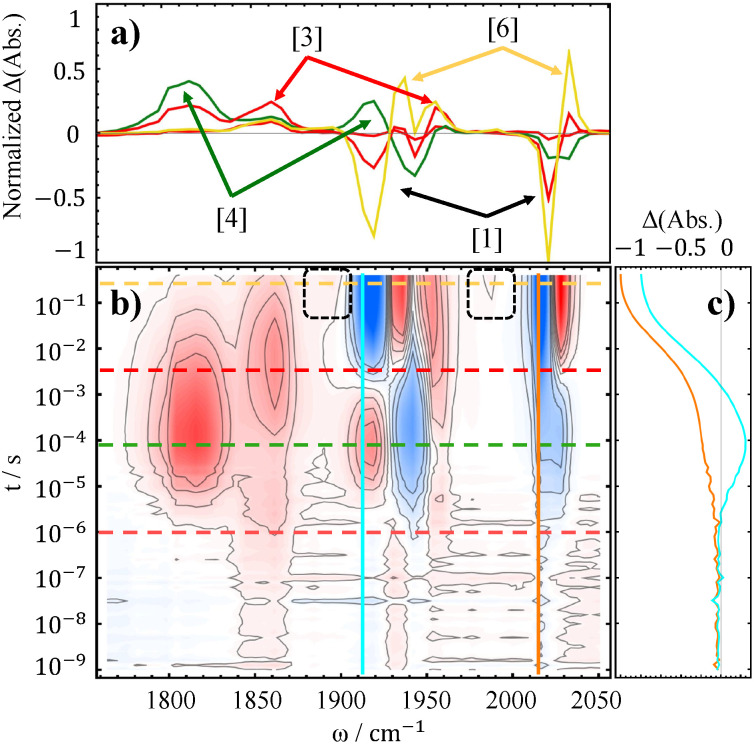
TRIR difference spectra of 10 mM Mn6mesb, 10 mM PS, 50 mM BNAH, and 1M TEOA in DMSO (b), accompanied by spectral cuts at selected delays (a), and time trace(s) (c). Dashed boxes depict regions of calculated bands for the hydride species.

To asses the effect of lowering proton concentration, the TRIR experiment was also carried out under additional 1 M TEOA (see Fig. 6). Although the final formation of [Mn^I^(L)(CO)_3_(DMSO)]^+^ was not suppressed, the reaction rate was noticeably slower (note the shift of the relevant bands to slower timescales). Consequently, [Mn^0^(L˙^−^)(CO)_3_]^−^ also “survives” much longer. Remarkably, the metal hydride intermediate, whose observation was regarded as challenging,^80^ builds up to a detectable concentration (see Fig. 6b, boxed bands). Although these faint bands are quite close to the noise level, their presence can be confirmed by inspecting the respective lifetime distribution map, see Fig. S17 in SI. This might seem counterintuitive since the solution contains a base, however, 1 M TEOA is not basic enough to prevent hydride formation completely. Instead, since in the absence of protons each metal hydride decays by reacting with another hydride, the base-induced decrease in its total concentration delays also this bimolecular reaction, and Mn^I^(L)(CO)_3_H becomes detectable. This hydride complex decay pathway by hydrogen evolution reaction is slightly different from the one shown in eqn (12), as dimer formation:15
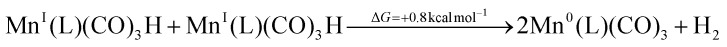
is excluded. The two Mn^0^(L)(CO)_3_ can again disproportionate leading to the solvato-complex ([Mn^I^(L)(CO)_3_(DMSO)]^+^) and [Mn^0^(L˙^−^)(CO)_3_]^−^ that re-enters the cycle. Besides these reactions, the hydride complex can be converted to [Mn^I^(L)(CO)_3_(DMSO)]^+^ by donating H^−^ to BNA^+^, regenerating BNAH:16
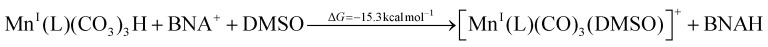


BNA^+^ could originate in the system as previously shown in eqn (9), or by another type of hydrogen evolution reaction:17
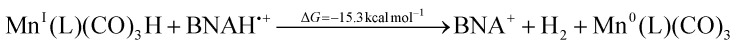


Although the precise occurrence of these reactions cannot be confirmed, the spectroscopic evidence strongly supports re-oxidation to the solvato-complex. We need to stress that the reactions (14)–(17) are presumptive, based only on the evidence obtained for two previous complexes, as well as DFT calculated reaction free energies. We propose them herein as possible pathways to explain the re-oxidation of the reduced complex to the final product, *i.e.*, [Mn^I^(L)(CO)_3_(DMSO)]^+^. As CO_2_ reduction is not taking place in this study, the electrons are either directed toward the aforementioned hydrogen evolution pathways or regeneration of the electron donor.

### 2.3. Influence of electron-withdrawing substituents on the ligand

All of the catalysts discussed so far contain electron-donating groups on the bipyridine ligand. A complex with the opposite type of substituent is Mn(CO)_3_Br(4,4′-dimethylester-2,2′-bipyridine), abbreviated as Mn4dicarb, with methyl ester substituents in 4,4′ positions on the bpy. The Bocarsly group^83^ studied a similar complex with a (2,2′-bipyridine)-4,4′-dicarboxylic acid as diimine ligand and reported diminished catalytic activity of the complex. They attributed this to the greater localization of the HOMO of the doubly-reduced complex on the ligand side rather than the metal center. They also predicted a slower dissociation of the axial Br^−^ ligand, a process we can potentially probe directly with TRIR to obtain additional insight into the activity of such complexes.

**Fig. 7:**
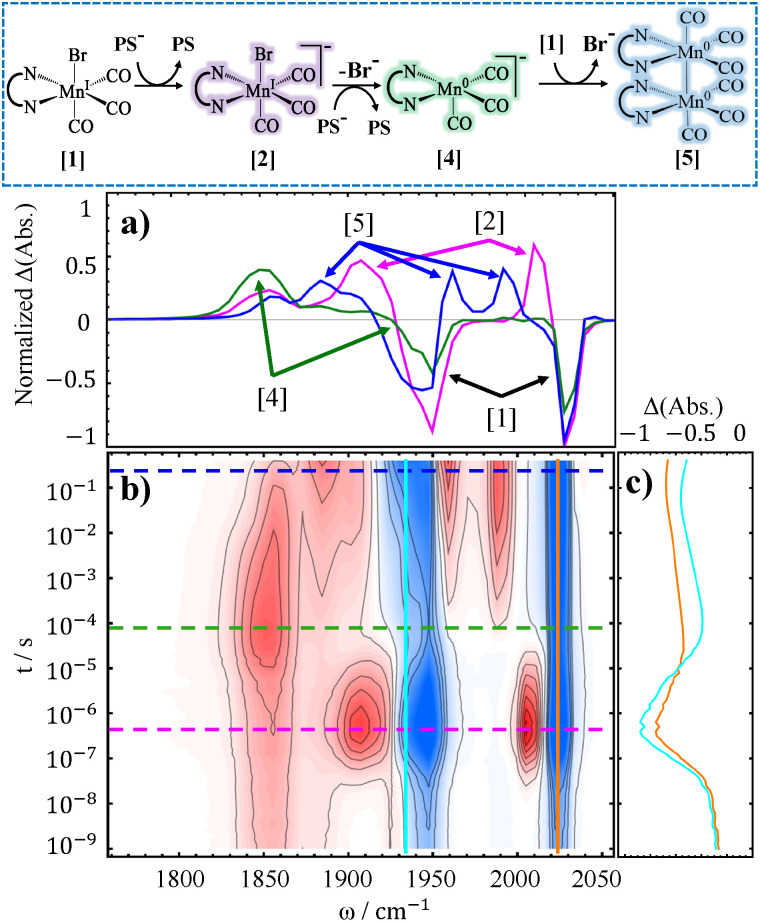
TRIR difference spectra of 10 mM Mn4dicarb, 10 mM PS, and 50 mM BNAH in DMSO (b), accompanied by spectral cuts at selected delays (a), and time trace(s) (c). The reaction pathway is shown in the blue box on top of the figure.

The TRIR spectrum of Mn4dicarb is shown in Fig. 7. Besides the expected intermolecular electron transfer reaction, the spectrum also contains a contribution from a photo-degradation of the catalyst, which is discussed in Section S3.1 of SI. As predicted by Bocarsly *et al.*,^83^ the Br^−^ ligand is not immediately lost upon one-electron reduction of the complex, instead the negatively charged intermediate [Mn^I^(L˙^−^)(CO)_3_Br]^−^ is observed (compound [2] in Fig. 7, violet structure, also shown in Fig. 2) on a ∼100 ns timescale. The reduction rate of Mn4dicarb is noticeably faster than that of the other three complexes, which is due to higher potential difference between this complex and PS^−^. The stronger localization of electron density on the ligand results in higher vibrational frequencies of the [Mn^I^(L˙^−^)(CO)_3_Br]^−^ carbonyls as the back-bonding effect from the metal center is suppressed. The subsequent radical with Br^−^ released is effectively a transition state with an energy of ∼+2.5 kcal mol^−1^. It is not observed, as its build-up is slower than the subsequent decay into the 2e-reduced [Mn^0^(L˙^−^)(CO)_3_]^−^ within ∼8 µs (compound [4] in Fig. 7, green structure). Of the modes of [Mn^0^(L˙^−^)(CO)_3_]^−^, the highest frequency one is completely canceled out by the superimposed bleach close to 1950 cm^−1^, but its presence can be inferred from the time trace at this frequency (Fig. 7c), which shows a positive slope. Furthermore, the time trace at the higher frequency bleach also shows a positive slope at approximately the same timescale of ∼8 µs. Two plausible explanations can be considered. First, there may be a solvent-coordinated intermediate that is in between Br^−^ loss and the second electron acceptance by the Mn^0^ species. Since solvent coordination shifts the electron density towards the ligand, which however is not desirable when there are electron-donating substituents on the bipyridine,^35^ when electron-withdrawing groups are present, it becomes thermodynamically more favorable. This explains the calculated solvent binding free energy of only +0.5 kcal mol^−1^ for DMSO coordination to Mn^0^(4dicarbpy)(CO)_3_*versus* +12.0 kcal mol^−1^ for Mn^0^(6dmbpy)(CO)_3_. The second, and more sound, hypothesis is back-electron transfer reaction from [Mn^I^(L˙^−^)(CO)_3_Br]^−^ to PS, which would recover the parent complex and thus reduce the bleach. The latter is more probable in the case of Mn4dicarb than, for example, Mn6dmb, where a chemical reaction follows directly after electron transfer.

## 3. Conclusions

In summary, we have elucidated the activation pathway of Mn(α-diimine)(CO)_3_Br-type catalysts employed in CO_2_ reduction. Thus far, only a few mechanistic studies have investigated the reaction dynamics using transient IR spectroscopy, and none have done so using a standard three-component chemical system. We provide a time-resolved spectroscopic perspective that follows the reaction pathway in such systems, while also revealing how modifications to the α-diimine ligand direct the selection of distinct reaction mechanisms.

In total four catalysts have been examined: Mn(4,4′-ditertbutyl-2,2′-bipyridine)(CO)_3_Br, Mn(6,6′-dimethyl-2,2′-bipyridine)(CO)_3_Br, Mn(6,6′-dimesityl-2,2′-bipyridine)(CO)_3_Br, and Mn(4,4′-dimethylester-2,2′-bipyridine)(CO)_3_Br. Each was chosen to exemplify a particular structural feature expected to influence catalytic behavior - whether by imposing steric constraints that suppress dimer formation or modulating electronic properties through electron-donating or electron-withdrawing substituents. These variations allowed us to systematically probe how specific ligand modifications translate into distinct mechanistic pathways, thereby providing a comparative framework for rational catalyst design.

**Fig. 8:**
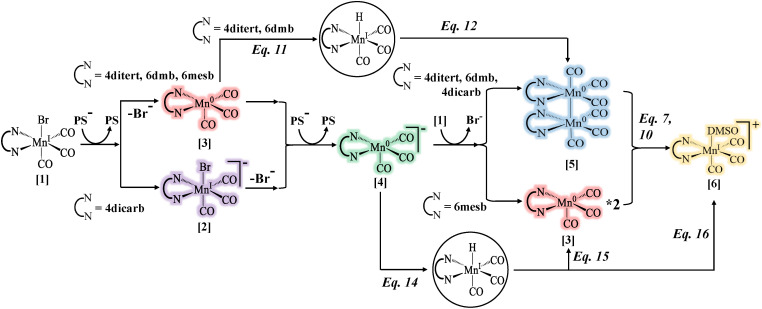
A summary of important pathways discussed in this study.

A common feature among the four catalysts is that the intermediate formed after initial reduction and Br^−^ dissociation, *i.e.*, Mn^0^(L)(CO)_3_, possesses a higher reduction potential than the resting state, which leads to a second reduction by PS˙^−^ (see Fig. 8, pathway from compound [1] to [4]). It has been known in the literature that the second reduction should either be at the same or higher potential than the first one based on cyclic voltammetry data of non-dimerizing catalyst - Mn(6,6′-dimesityl-2,2′-bipyridine)(CO)_3_Br - which exhibits a single peak towards [Mn^0^(L˙^−^)(CO)_3_]^−^.^44^ Using our time-resolved approach, we were able to directly capture this process and confirm the prediction. Furthermore, in systems where dimer formation is not sterically hindered, the second reduction proceeds substantially faster than dimerization between two Mn^0^ radicals. The dimer, [Mn^0^(L)(CO)_3_]_2_, still forms, but only after all the PS˙^−^ is exhausted in the system and proceeds *via* a symproportionation reaction between the parent complex and the doubly-reduced one (see Fig. 8, pathway from compound [4] to [5]).

Moreover, for complexes that form a dimer, its re-oxidation was documented in the millisecond regime, caused by acidic proton attack from BNAH˙^+^ forming a metal hydride - Mn^I^(L)(CO)_3_H and solvato-complex [Mn^I^(L)(CO)_3_(DMSO)]^+^ (Fig. 8, pathway from compound [5] to [6]). This process is, as expected, influenced by the concentration of base in solution, that was confirmed by introducing TEOA in the experiments. For a non-dimerizing pathway, the re-oxidation takes place by the proton attack on [Mn^0^(L˙^−^)(CO)_3_]^−^ species (Fig. 8, compound [4] *via*eqn (14)), or by hydrogen atom transfer from BNAH˙^+^ to Mn^0^(L)(CO)_3_ (Fig. 8, compound [3] *via*eqn (11)), both reactions leading to Mn^I^(L)(CO)_3_H. The latter again decays by hydride transfer to BNA^+^, regenerating the electron donor and producing the solvato-complex (Fig. 8, eqn (16)). While the other metal hydride depletion pathways are *via* hydrogen evolution reactions (Fig. 8, eqn (15) and (17)), both of which lead to Mn^0^(L)(CO)_3_, whose disproportionation also populates the final solvato-complex (compound [6] in Fig. 8).

The evidence presented here indicates that in the absence of CO_2_ reduction, the available electrons are instead diverted either into H_2_ evolution pathways or back toward regeneration of the electron donor. Among the reported intermediates, both [Mn^0^(L˙^−^)(CO)_3_]^−^ and hydride complexes, Mn^I^(L)(CO)_3_H, have been proposed as catalytically active species, preferentially leading to CO or formate, respectively,^51,84–87^ while some studies even show the evidence of the dimer's involvement.^43,88^ However, DFT-calculated hydricities^89^ of the hydride species investigated herein all exceed that of the formate anion in DMSO (∼29.0 kcal mol^−1^, see Section S5.1 in SI), indicating that these hydrides cannot reduce CO_2_. Consequently, suppression of the hydride pathway is necessary for achieving efficient CO_2_ reduction. This can be accomplished by introducing a suitable base into the solution. Moreover, since Mn-based catalysts require the presence of Brønsted acids to promote catalysis,^54^ the choice of base becomes critical. Ideally, the base should be sufficiently strong to deprotonate the oxidized electron donor, yet not so strong as to also deprotonate weaker acids present in solution. TEOA is a good starting choice, as in addition to being a relatively weak base, it reportedly promotes CO_2_ binding.^51^

In future work, we plan to investigate the pathway of CO_2_ reduction itself in greater detail. The experiments discussed herein were intentionally conducted in the absence of CO_2_ in order to disentangle the intrinsic electron–transfer processes of the complex 3-component system from substrate–specific reactivity. Establishing such a baseline understanding is essential before introducing CO_2_, as its presence can significantly alter the mechanistic landscape by opening new reaction channels. The spectra, which are already convoluted without the presence of CO_2_, will only become more complex. The mechanistic insights gained from the present study therefore serve as a crucial reference point for interpreting the subsequent catalytic behavior under CO_2_–reducing conditions and for rationally designing experiments that more effectively probe the full photo-catalytic cycle.

The findings presented herein allow us to distill several key conclusions. It will be important to avoid dimerization by symproportionation to prolong the lifetime of the doubly-reduced species. This will be achieved by using BIH as electron donor, instead of BNAH, as BI˙ is strong enough to reduce even the dimeric species.^51^ Moreover, a Brønsted acid will also be introduced to provide protons needed for the CO_2_ reduction, but it should be rather weak to avoid protonation of the metal center that would lead to competing hydrogen evolution pathways. Quite a few studies have focused on designing catalysts with intramolecular acidic hydrogen source near the catalytically active site.^84,85,90–96^ In order to increase the probability of detecting all the important intermediates, we plan to use such local hydrogen bond donor ligand in our future works.

Overall, the present study establishes a foundation for future investigations of the complete CO_2_ reduction catalytic cycle using time-resolved spectroscopy. Such studies will be instrumental in identifying the key conditions that must be considered when designing or deploying new catalysts.

## Conflicts of interest

There are no conflicts of interest to declare.

## Supplementary Material

CP-028-D5CP03590B-s001

## Data Availability

In the supplementary information (SI) one can find synthesis methods (Section S1), steady-state and control experiments (Section S2), TRIR experimental details (Section S3) and the data related to the catalyst photo-degradation (Section S3.1), data analysis (Section S4), as well as computational chemistry details and results (Section S5). See DOI: https://doi.org/10.1039/d5cp03590b. Data of all figures have been deposited in Zenodo: https://doi.org/10.5281/zenodo.17965049.

## References

[cit1] Nunes L. J. (2023). The Rising Threat of Atmospheric CO_2_: A Review on The Causes, Impacts, and Mitigation Strategies. Environments.

[cit2] Al-Ghussain L. (2019). Global Warming: Review on Driving Forces and Mitigation. Environ. Prog. Sustain. Energy.

[cit3] Ravanchi M. T., Sahebdelfar S. (2021). Catalytic Conversions of CO_2_ to Help Mitigate Climate Change: Recent Process Developments. Process Saf. Environ. Prot..

[cit4] Benson E. E., Kubiak C. P., Sathrum A. J., Smieja J. M. (2009). Electrocatalytic and Homogeneous Approaches to Conversion of CO_2_ to Liquid Fuels. Chem. Soc. Rev..

[cit5] Zhang S., Li M., Qiu W., Han J., Wang H., Liu X. (2019). Heterogeneous Molecular Rhenium Catalyst for CO_2_ Photoreduction with High Activity and Tailored Selectivity in an Aqueous Solution. Appl. Catal., B.

[cit6] Zhang S., Fan Q., Xia R., Meyer T. J. (2020). CO_2_ Reduction: from Homogeneous to Heterogeneous Electrocatalysis. Acc. Chem. Res..

[cit7] Hawecker J., Lehn J.-M., Ziessel R. (1983). Efficient Photochemical Reduction of CO_2_ to CO by Visible Light Irradiation of Systems Containing Re(bipy)(CO)_3_ X or Ru(bipy)_3_^2+^-Co^2+^ combinations as Homogeneous Catalysts. J. Chem. Soc., Chem. Commun..

[cit8] Sullivan B. P., Bolinger C. M., Conrad D., Vining W. J., Meyer T. J. (1985). One-and two-electron pathways in the electrocatalytic reduction of CO_2_ by *fac*-Re(bpy)(CO)_3_ Cl (bpy= 2,2'-bipyridine). J. Chem. Soc., Chem. Commun..

[cit9] Gholamkhass B., Mametsuka H., Koike K., Tanabe T., Furue M., Ishitani O. (2005). Architecture of Supramolecular Metal Complexes for Photocatalytic CO_2_ Reduction: Ruthenium-Rhenium Bi- and Tetranuclear Complexes. Inorg. Chem..

[cit10] Ishida H., Tanaka K., Tanaka T. (1987). Electrochemical CO_2_ Reduction Catalyzed by Ruthenium Complexes [Ru(bpy)_2_ (CO)_2_ ]^2 +^ and [Ru(bpy)_2_ (CO)Cl]^+^. Effect of pH on The Formation of CO and HCOO^−^. Organometallics.

[cit11] Ishida H., Terada T., Tanaka K., Tanaka T. (1990). Photochemical Carbon Dioxide Reduction Catalyzed by Bis(2,2'-bipyridine)dicarbonylruthenium(2 +) Using Triethanolamine and 1-Benzyl-1,4-dihydronicotinamide as an Electron Donor. Inorg. Chem..

[cit12] Rasmussen S. C., Richter M. M., Yi E., Place H., Brewer K. J. (1990). Synthesis and Characterization of A Series of Novel Rhodium and Iridium Complexes Containing Polypyridyl Bridging Ligands: Potential Uses in The Development of Multimetal Catalysts for Carbon Dioxide Reduction. Inorg. Chem..

[cit13] Bolinger C. M., Story N., Sullivan B. P., Meyer T. J. (1988). Electrocatalytic Reduction of Carbon Dioxide by 2,2'-Bipyridine Complexes of Rhodium and Iridium. Inorg. Chem..

[cit14] Bourrez M., Molton F., Chardon-Noblat S., Deronzier A. (2011). [Mn (bipyridyl)(CO)_3_ Br]: An Abundant Metal Carbonyl Complex as an Efficient Electrocatalyst for CO_2_ Reduction. Angew. Chem., Int. Ed..

[cit15] Appel A. M., Bercaw J. E., Bocarsly A. B., Dobbek H., DuBois D. L., Dupuis M., Ferry J. G., Fujita E., Hille R., Kenis P. J. (2013). *et al.*, Frontiers, Opportunities, and Challenges in Biochemical and Chemical Catalysis of CO_2_ Fixation. Chem. Rev..

[cit16] Hawecker J., Lehn J.-M., Ziessel R. (1986). Photochemical and Electrochemical Reduction of Carbon Dioxide to Carbon Monoxide Mediated by (2,2'-Bipyridine)tricarbonylchlororhenium(I) and Related Complexes as Homogeneous Catalysts. Helv. Chim. Acta.

[cit17] Arakawa H., Aresta M., Armor J. N., Barteau M. A., Beckman E. J., Bell A. T., Bercaw J. E., Creutz C., Dinjus E., Dixon D. A. (2001). *et al.*, Catalysis Research of Relevance to Carbon Management: Progress, Challenges, and Opportunities. Chem. Rev..

[cit18] Morris A. J., Meyer G. J., Fujita E. (2009). Molecular Approaches to the Photocatalytic Reduction of Carbon Dioxide for Solar Fuels. Acc. Chem. Res..

[cit19] Doherty M. D., Grills D. C., Muckerman J. T., Polyansky D. E., Fujita E. (2010). Toward more efficient photochemical CO_2_ reduction: Use of CO_2_ or photogenerated hydrides. Coord. Chem. Rev..

[cit20] Grills D. C., Fujita E. (2010). New Directions for the Photocatalytic Reduction of CO_2_: Supramolecular, CO_2_ or Biphasic Ionic Liquid-CO_2_ Systems. J. Phys. Chem. Lett..

[cit21] Mikkelsen M., Jørgensen M., Krebs F. C. (2010). The teraton challenge. A review of fixation and transformation of carbon dioxide. Energy Environ. Sci..

[cit22] Takeda H., Ishitani O. (2010). Development of Efficient Photocatalytic Systems for CO_2_ Reduction Using Mononuclear and Multinuclear Metal Complexes Based on Mechanistic Studies. Coord. Chem. Rev..

[cit23] Costentin C., Robert M., Savéant J.-M. (2013). Catalysis of the electrochemical reduction of carbon dioxide. Chem. Soc. Rev..

[cit24] Qiao J., Liu Y., Hong F., Zhang J. (2014). A review of catalysts for the electroreduction of carbon dioxide to produce low-carbon fuels. Chem. Soc. Rev..

[cit25] Yamazaki Y., Takeda H., Ishitani O. (2015). Photocatalytic reduction of CO_2_ using metal complexes. J. Photochem. Photobiol. C: Photochem. Rev..

[cit26] Elgrishi N., Chambers B., Wang M., Fontecave X. (2017). M. Molecular Polypyridine-Based Metal Complexes as Catalysts for the Reduction of CO_2_. Chem. Soc. Rev..

[cit27] Grice K. A. (2017). Carbon Dioxide Reduction with Homogenous Early Transition Metal Complexes: Opportunities and Challenges for Developing CO_2_ Catalysis. Coord. Chem. Rev..

[cit28] Takeda H., Cometto C., Ishitani O., Robert M. (2017). Electrons, Photons, Protons and Earth-Abundant Metal Complexes for Molecular Catalysis of CO_2_ Reduction. ACS Catal..

[cit29] Francke R., Schille B., Roemelt M. (2018). Homogeneously Catalyzed Electroreduction of Carbon Dioxide–Methods, Mechanisms, and Catalysts. Chem. Rev..

[cit30] Fairlamb I. J., Lynam J. M. (2024). Unveiling Mechanistic Complexity in Manganese-Catalyzed C-H Bond Functionalization Using IR Spectroscopy Over 16 Orders of Magnitude in Time. Acc. Chem. Res..

[cit31] Farmer A. L., Procacci B., Shaw D. J., Gurung S., Fairlamb I. J., Lynam J. M., Hunt N. T. (2025). Ultrafast vibrational spectroscopic analysis of the ubiquitous precatalyst [Mn_2_ (CO)_10_] in different solvents. J. Chem. Phys..

[cit32] Eastwood J. B., Procacci B., Gurung S., Lynam J. M., Hunt N. T. (2024). Understanding the Vibrational Structure and Ultrafast Dynamics of the Metal Carbonyl Precatalyst [Mn(ppy)(CO)_4_]. ACS Phys. Chem. Au.

[cit33] Eastwood J. B., Burden T. J., Hammarback L. A., Horbaczewskyj C., Tanner T. F., Clark I. P., Greetham G., Towrie M., Fairlamb I. J., Lynam J. M. (2024). The importance of understanding (pre)catalyst activation in versatile C-H bond functionalisations catalysed by [Mn_2_ (CO)_10_]. Chem. Sci..

[cit34] Burden T. J., Fernandez K. P., Kagoro M., Eastwood J. B., Tanner T. F., Whitwood A. C., Clark I. P., Towrie M., Krieger J.-P., Lynam J. M. (2023). *et al.*, Coumarin C-H Functionalization
by Mn(I) Carbonyls: Mechanistic Insight by Ultra-Fast IR Spectroscopic Analysis. Chem. – Eur. J..

[cit35] Grills D. C., Farrington J. A., Layne B. H., Lymar S. V., Mello B. A., Preses J. M., Wishart J. F. (2014). Mechanism of the Formation of a Mn-Based CO_2_ Reduction Catalyst Revealed by Pulse Radiolysis with Time-Resolved Infrared Detection. J. Am. Chem. Soc..

[cit36] Martinez J. F., La Porte N. T., Young R. M., Sinopoli A., Sohail M., Wasielewski M. R. (2019). Direct Observation of the Photoreduction Products of Mn(NDI-bpy)(CO)3X CO2 Reduction Catalysts Using Femtosecond Transient IR Spectroscopy. J. Phys. Chem. C.

[cit37] Chattopadhyay S., Barman S., Lomoth R., Hammarström L. (2025). Unraveling Bifurcating Pathways for CO and HCOOH Formation: Insights from Stopped-Flow FTIR Spectroscopy of a Second-Sphere Modified Mn Catalyst. J. Am. Chem. Soc..

[cit38] Takeda H., Koizumi H., Okamoto K., Ishitani O. (2014). Photocatalytic CO_2_ Reduction Using A Mn Complex as A Catalyst. Chem. Commun..

[cit39] Stor G. J., Morrison S. L., Stufkens D. J., Oskam A. (1994). The Remarkable Photochemistry of *fac*-XMn(CO)_3_ (α -diimine)(X = Halide): Formation of Mn_2_ (CO)_6_ (α -diimine)_2_ via the mer Isomer and Photocatalytic Substitution of X^−^ in the Presence of PR_3_. Organometallics.

[cit40] Yempally V., Moncho S., Hasanayn F., Fan W. Y., Brothers E. N., Bengali A. A. (2017). Ancillary Ligand Effects upon the Photochemistry of Mn(bpy)(CO)_3_ X Complexes (X = Br^−^, PhCC^−^). Inorg. Chem..

[cit41] Pordel S., White J. K. (2020). Impact of Mn(I) photoCORM ligand set on photochemical intermediate formation during visible light-activated CO release. Inorg. Chim. Acta.

[cit42] Pordel S., Schrage B. R., Ziegler C. J., White J. K. (2020). Impact of steric bulk on photoinduced ligand exchange reactions in Mn(I) photoCORMs. Inorg. Chim. Acta.

[cit43] Siritanaratkul B., Eagle C., Cowan A. J. (2022). Manganese Carbonyl Complexes as Selective Electrocatalysts for CO_2_ Reduction in Water and Organic Solvents. Acc. Chem. Res..

[cit44] Sampson M. D., Nguyen A. D., Grice K. A., Moore C. E., Rheingold A. L., Kubiak C. P. (2014). Manganese Catalysts with Bulky Bipyridine Ligands for the Electrocatalytic Reduction of Carbon Dioxide: Eliminating Dimerization and Altering Catalysis. J. Am. Chem. Soc..

[cit45] Pellegrin Y., Odobel F. (2017). Sacrificial Electron Donor Reagents for Solar Fuel Production. C. R. Chim..

[cit46] De Kreijger S., Gillard M., Elias B., Troian-Gautier L. (2024). Spectroscopic Techniques to Unravel Mechanistic Details in Light-Induced Transformations and Photoredox Catalysis. ChemCatChem.

[cit47] Fukuzumi S., Koumitsu S., Hironaka K., Tanaka T. (1987). Energetic Comparison between Photoinduced Electron-Transfer Reactions from NADH Model Compounds to Organic and Inorganic Oxidants and Hydride-Transfer Reactions from NADH Model Compounds to p-Benzoquinone Derivatives. J. Am. Chem. Soc..

[cit48] Staal L., Oskam A., Vrieze K. (1979). The Syntheses and Coordination Properties of M(CO)_3_ X(DAB) (M = Mn, Re; X= Cl, Br, I; DAB= 1,4-Diazabutadiene). J. Organomet. Chem..

[cit49] Schmittel M., Ganz A., Schenk W. A., Hagel M. (1999). Synthesis and Coordination Properties of 6,6'-Dimesityl-2,2'-bipyridine. Z. Naturforsch. B.

[cit50] O’Donoghue B. R., Flesch S., Courtney E., Choudhary S., Eastwood J. B., Mackey K., Pardo L. M., Clark I. P., Malakar P., Greetham G. M. (2025). *et al.*, Probing Mn Precatalyst Activation through Time-Resolved Spectroscopy: A Quantitative Evaluation
of the Effects of CO and PPh_3_ as Coligands on Ultrafast Dynamics and C–C Bond Formation. Inorg. Chem..

[cit51] Koizumi H., Tamaki Y., Kamogawa K., Nicaso M., Suzuki Y., Yamazaki Y., Takeda H., Ishitani O. (2025). Development of a Highly Durable Photocatalytic CO_2_ Reduction Using a Mn-Complex Catalyst: Application of Selective Photosplitting of a Mn(0)-Mn(0) Bond. J. Am. Chem. Soc..

[cit52] Farrell K. M., Ostrander J. S., Jones A. C., Yakami B. R., Dicke S. S., Middleton C. T., Hamm P., Zanni M. T. (2020). Shot-to-Shot 2D IR Spectroscopy at 100 kHz Using a Yb Laser and Custom-Designed Electronics. Opt. Express.

[cit53] Buhrke D., Ruf J., Heckmeier P., Hamm P. (2021). A Stop-Flow Sample Delivery System for Transient Spectroscopy. Rev. Sci. Instrum..

[cit54] Smieja J. M., Sampson M. D., Grice K. A., Benson E. E., Froehlich J. D., Kubiak C. P. (2013). Manganese as a Substitute for Rhenium in CO_2_ Reduction Catalysts: The Importance of Acids. Inorg. Chem..

[cit55] Rossenaar B. D., Hartl F., Stufkens D. J., Amatore C., Maisonhaute E., Verpeaux J.-N. (1997). Electrochemical and IR/UV- Vis Spectroelectrochemical Studies of *fac*-[Mn(X)(CO)_3_ (iPr-DAB)]^*n*^ (n = 0, X = Br, Me, Bz; n = +1, X = THF, MeCN, nPrCN, P(OMe)_3_; iPr-DAB= 1,4-Diisopropyl-1,4-diaza-1,3-butadiene) at Variable Temperatures: Relation between Electrochemical and Photochemical Generation of [Mn(CO)_3_ (α -diimine)]^−^. Organometallics.

[cit56] Zeng Q., Tory J., Hartl F. (2014). Electrocatalytic Reduction of Carbon Dioxide with a Manganese(I) Tricarbonyl Complex Containing a Nonaromatic α -Diimine Ligand. Organometallics.

[cit57] Lorenz-Fonfra V. A., Kandori H. (2006). Transformation of Time-Resolved Spectra to Lifetime-Resolved Spectra by Maximum Entropy Inversion of the Laplace Transform. Appl. Spectrosc..

[cit58] Hobson M., Lasenby A. (1998). The Entropic Prior for Distributions with Positive and Negative Values. Mon. Not. R. Astron. Soc..

[cit59] Lórenz-Fonfra V. A., Kandori H. (2007). Practical Aspects of the Maximum Entropy Inversion of the Laplace Transform for the Quantitative Analysis of Multi-Exponential Data. Appl. Spectrosc..

[cit60] Anderson P. A., Deacon G. B., Haarmann K. H., Keene F. R., Meyer T. J., Reitsma D. A., Skelton B. W., Strouse G. F., Thomas N. C. (1995). Designed Synthesis of Mononuclear Tris(heteroleptic) Ruthenium Complexes Containing Bidentate Polypyridyl Ligands. Inorg. Chem..

[cit61] Beckwith J. S., Rumble C. A., Vauthey E. (2020). Data Analysis in Transient Electronic Spectroscopy-An Experimentalist's View. Int. Rev. Phys. Chem..

[cit62] Buhrke D., Oppelt K. T., Heckmeier P. J., Fernandez-Teran R., Hamm P. (2020). Nanosecond Protein Dynamics in A Red/Green Cyanobacteriochrome Revealed by Transient IR Spectroscopy. J. Chem. Phys..

[cit63] Frisch M. J. et al. , Gaussian-16 Revision A.03, 2016; Gaussian Inc., Wallingford CT

[cit64] Zhao Y., Truhlar D. G. (2008). The M06 suite of density functionals for main group thermochemistry, thermochemical kinetics, noncovalent interactions, excited states, and transition elements: two new functionals and systematic testing of four M06-class functionals and 12 other functionals. Theor. Chem. Acc..

[cit65] Grimme S., Antony J., Ehrlich S., Krieg H. (2010). A consistent and accurate ab initio parametrization of density functional dispersion correction (DFT-D) for the 94 elements H-Pu. J. Chem. Phys..

[cit66] Weigend F., Ahlrichs R. (2005). Balanced Basis Sets of Split Valence, Triple Zeta Valence and Quadruple Zeta Valence Quality for H to Rn: Design and Assessment of Accuracy. Phys. Chem. Chem. Phys..

[cit67] Li H., Pomelli C. S., Jensen J. H. (2003). Continuum solvation of large molecules described by QM/MM: a semi-iterative implementation of the PCM/EFP interface. Theor. Chem. Acc..

[cit68] Li H., Jensen J. H. (2004). Improving the efficiency and convergence of geometry optimization with the polarizable continuum model: New energy gradients and molecular surface tessellation. J. Comput. Chem..

[cit69] Ochterski J. W. , Thermochemistry in Gaussian, Gaussian Inc, 2000, 1

[cit70] Hanwell M. D., Curtis D. E., Lonie D. C., Vandermeersch T., Zurek E., Hutchison G. R. (2012). Avogadro: An Advanced Semantic Chemical Editor, Visualization, and Analysis Platform. J. Cheminformatics.

[cit71] Qian B.-C., Zhu X.-Q., Shen G.-B. (2025). Thermodynamic Cards of Classic NADH Models and Their Related Photoexcited States Releasing Hydrides in Nine Elementary Steps and Their Applications. Molecules.

[cit72] Pavlishchuk V. V., Addison A. W. (2000). Conversion constants for redox potentials measured versus different reference electrodes in acetonitrile solutions at 25 C. Inorg. Chim. Acta.

[cit73] Grills D. C., Ertem M. Z., McKinnon M., Ngo K. T., Rochford J. (2018). Mechanistic Aspects of CO_2_ Reduction Catalysis with Manganese-Based Molecular Catalysts. Coord. Chem. Rev..

[cit74] Agarwal J., Stanton III C. J., Shaw T. W., Vandezande J. E., Majetich G. F., Bocarsly A. B., Schaefer III H. F. (2015). Exploring the effect of axial ligand substitution (X = Br, NCS, CN) on the photodecomposition and electrochemical activity of [MnX(N-C)(CO)_3_] complexes. Dalton Trans..

[cit75] Walsh J. J., Smith C. L., Neri G., Whitehead G. F., Robertson C. M., Cowan A. J. (2015). Improving the efficiency of electrochemical CO_2_ reduction using immobilized manganese complexes. Faraday Discuss..

[cit76] Haukka M., Hirva P., Luukkanen S., Kallinen M., Ahlgrén M., Pakkanen T. A. (1999). Reactions of Ruthenium Bipyridine Catalyst Precursors: Synthetic, Structural, and Theoretical Studies on Ruthenium Mono (Bipyridine) Carbonyls in Ethylene Glycol Solutions. Inorg. Chem..

[cit77] Kuramochi Y., Ito Y., Ishida H. (2012). Chain Reaction for Isomerization from trans(Cl) to cis(Cl)-Ru(bpy)(CO)_2_ Cl_2_ (bpy = 2,2'-Bipyridine) Induced by NaBH_4_. Eur. J. Inorg. Chem..

[cit78] Balducci G., Iengo E., Demitri N., Alessio E. (2015). New Insight into a Deceptively Simple Reaction: The Coordination of bpy to RuII–Carbonyl Precursors – The Central Role of the *fac*-[Ru(bpy)Cl(CO)3]+ Intermediate and the Chloride Rebound Mechanism. Eur. J. Inorg. Chem..

[cit79] Hall Jr H. (1957). Correlation of the Base Strengths of Amines. J. Am. Chem. Soc..

[cit80] Garcia Alonso F. J., Llamazares A., Riera V., Vivanco M., Garcia Granda S., Diaz M. R. (1992). Effect of an nitrogen-nitrogen chelate ligand on the insertion reactions of carbon monoxide into a manganese-alkyl bond. Organometallics.

[cit81] Dey S., Masero F., Brack E., Fontecave M., Mougel V. (2022). Electrocatalytic metal hydride generation using CPET mediators. Nature.

[cit82] Sampson M. D., Kubiak C. P. (2016). Manganese Electrocatalysts with Bulky Bipyridine Ligands: Utilizing Lewis Acids To Promote Carbon Dioxide Reduction at Low Overpotentials. J. Am. Chem. Soc..

[cit83] Tignor S. E., Kuo H.-Y., Lee T. S., Scholes G. D., Bocarsly A. B. (2018). Manganese-Based Catalysts with Varying Ligand Substituents for the Electrochemical Reduction of CO_2_ to CO. Organometallics.

[cit84] Rønne M. H., Cho D., Madsen M. R., Jakobsen J. B., Eom S., Escoudé É., Hammershøj H. C. D., Nielsen D. U., Pedersen S. U., Baik M.-H. (2020). *et al.*, Ligand-Controlled Product Selectivity in Electrochemical Carbon Dioxide Reduction Using Manganese Bipyridine Catalysts. J. Am. Chem. Soc..

[cit85] Roy S. S., Talukdar K., Jurss J. W. (2021). Electro- and Photochemical Reduction of CO_2_ by Molecular Manganese Catalysts: Exploring the Positional Effect of Second-Sphere Hydrogen-Bond Donors. ChemSusChem.

[cit86] Wang X., Thiel I., Fedorov A., Copéret C., Mougel V., Fontecave M. (2017). Site-isolated manganese carbonyl on bipyridine-functionalities of periodic mesoporous organosilicas: Efficient CO_2_ photoreduction and detection of key reaction intermediates. Chem. Sci..

[cit87] Hong W., Luthra M., Jakobsen J. B., Madsen M. R., Castro A. C., Hammershøj H. C. D., Pedersen S. U., Balcells D., Skrydstrup T., Daasbjerg K. (2023). *et al.*, Exploring the Parameters Controlling Product Selectivity in Electrochemical CO_2_ Reduction in Competition with Hydrogen Evolution Employing Manganese Bipyridine Complexes. ACS Catal..

[cit88] Bourrez M., Orio M., Molton F., Vezin H., Duboc C., Deronzier A., Chardon-Noblat S. (2014). Pulsed-EPR Evidence of a Manganese(II) Hydroxycarbonyl Intermediate in the Electrocatalytic Reduction of Carbon Dioxide by a Manganese Bipyridyl Derivative. Angew. Chem., Int. Ed..

[cit89] Waldie K. M., Ostericher A. L., Reineke M. H., Sasayama A. F., Kubiak C. P. (2018). Hydricity of Transition-Metal Hydrides: Thermodynamic Considerations for CO_2_ Reduction. ACS Catal..

[cit90] Franco F., Cometto C., Vallana F. F., Sordello F., Priola E., Minero C., Nervi C., Gobetto R. (2014). A local proton source in a [Mn(bpy-R)(CO)_3_ Br]-type redox catalyst enables CO_2_ reduction even in the absence of Brønsted acids. Chem. Commun..

[cit91] Agarwal J., Shaw T. W., Schaefer III H. F., Bocarsly A. B. (2015). Design of a Catalytic Active Site for Electrochemical CO_2_ Reduction with Mn(I)-Tricarbonyl Species. Inorg. Chem..

[cit92] Ngo K. T., McKinnon M., Mahanti B., Narayanan R., Grills D. C., Ertem M. Z., Rochford J. (2017). Turning on the Protonation-First Pathway for Electrocatalytic CO_2_ Reduction by Manganese Bipyridyl Tricarbonyl Complexes. J. Am. Chem. Soc..

[cit93] Franco F., Cometto C., Nencini L., Barolo C., Sordello F., Minero C., Fiedler J., Robert M., Gobetto R., Nervi C. (2017). Local Proton Source in Electrocatalytic CO_2_ Reduction with [Mn(bpy-R)(CO)_3_ Br] Complexes. Chem. – Eur. J..

[cit94] Sung S., Li X., Wolf L. M., Meeder J. R., Bhuvanesh N. S., Grice K. A., Panetier J. A., Nippe M. (2019). Synergistic Effects of Imidazolium-Functionalization on *fac*-Mn(CO)_3_ Bipyridine Catalyst Platforms for Electrocatalytic Carbon Dioxide Reduction. J. Am. Chem. Soc..

[cit95] Qing Y., Wu Q., He S., Zhang P., Xiong Y., Zhang Y., Huang F., Li F., Chen L. (2023). Effects of proton tunneling distance on CO_2_ reduction by Mn terpyridine species. Dalton Trans..

[cit96] Li M., Huang F., Zhang P., Xiong Y., Zhang Y., Li F., Chen L. (2024). Electrochemical CO_2_ Reduction by Urea Hangman Mn Terpyridine species. Chem. – Eur. J..

