# Supplementary material for: Monomer and dimer pathways of earth-abundant manganese tricarbonyl pre-catalysts for CO2 reduction studied by time-resolved IR spectroscopy
Source: Phys Chem Chem Phys. 2025 Dec 23;28(3):2281–93. doi: 10.1039/d5cp03590b (PMC12767779; doi:10.1039/d5cp03590b)
Supplement: CP-028-D5CP03590B-s001 [file CP-028-D5CP03590B-s001.pdf]

Supplementary Information (SI) for Physical Chemistry Chemical Physics.
This journal is © the Owner Societies 2026

Supporting Information: Monomer and Dimer Pathways of Earth-abundant Manganese Tricarbonyl Pre-catalysts for CO_2 Reduction Studied by Time-resolved IR Spectroscopy

Luka Tatarashvili, Noah von Fellenberg, Kerstin Oppelt, and Peter Hamm

Department of Chemistry, University of Zürich, Zürich, Switzerland

Contents

1 Synthesis **2**

1.1 Materials . . . 2

1.2 Synthesis of Mn6dmb . . . 3

1.3 Synthesis of Mn4ditert . . . 4

1.4 Synthesis of Mn4dicarb . . . 4

1.5 Synthesis of Mn6mesb . . . 5

1.5.1 6,6'-dimesityl-2.2'-dipyridine (6mesbpy) . . . 5

1.5.2 Synthesis of Mn6mesb . . . 6

2 Steady-state and Control Experiments **7**

2.1 Solvolysis Experiments . . . 9

3 TRIR **11**

3.1 Photodegradation Side Reaction of Mn4dicarb . . . 11

4 Data Analysis **13**

4.1 Gathering evidence for the elusive metal hydride 17

4.2 Mn4dicarb photo-degradation disentangled 19

5 Computational Chemistry **21**

5.1 Calculating Hydricities 23

5.2 Cartesian Coordinates for the reported species 25

5.2.1 Coordinates for Mn6dmb species 25

5.2.2 Coordinates for Mn4ditert species 37

5.2.3 Coordinates for Mn6mesb species 59

5.2.4 Coordinates for Mn4dicarb species 75

5.2.5 Coordinates for extra species 94

References **101**

1 Synthesis

1.1 Materials

All chemicals were used as received. $Mn(CO)_5Br$ (98%), Triethanolamine (≥99%), Toluene (EMSURE), and $Ru(bpy)_3(PF_6)_2$ (97%) were purchased from Sigma-Aldrich/Merck. DMSO (99+%) and 6,6'-dimethyl-2,2'-dipyridyl (98%) were obtained from Thermo Fischer Scientific. Dimethyl-[2,2'-bipyridine]-4,4'-dicarboxylate (95%) was purchased from Fluorochem. 4,4'-bis(tert-butyl)-2,2'-bipyridine (98%) was obtained from Apollo Scientific. 6,6'-dibromo-2,2'-bipyridine (≥97%) and 2,4,6-trimethylphenylboronic acid (≥98%) were purchased from ChemScene. Diethylether (≥99.5%) was obtained from Roth.

1.2 Synthesis of Mn6dmb

The synthesis of Mn6dmb was done using the procedure from literature[1–3] with a few differences that follow. 281.2 mg (1.02 mmol) of $Mn(CO)_5Br$ and 226.5 mg (1.23 mmol) of 6,6'-dimethyl-2,2'-dipyridine were put into a 100 mL brown glass round-bottom flask. 30 mL of diethyl ether added, and bubbled for ∼5 min. with N_2. The solution was then stirred and heated to reflux under N_2 atmosphere. The reflux was maintained overnight (∼17 hr). During this period, the reaction vessel was carefully covered with Aluminum foil and room lights were also limited. After cooling the solution to room temperature, vacuum filtration was used to separate the yellow precipitate, washed with cold diethyl ether. The solid was dried overnight under vacuum in darkness. The product weight was 349.5 mg (0.867 mmol, 85 % yield).

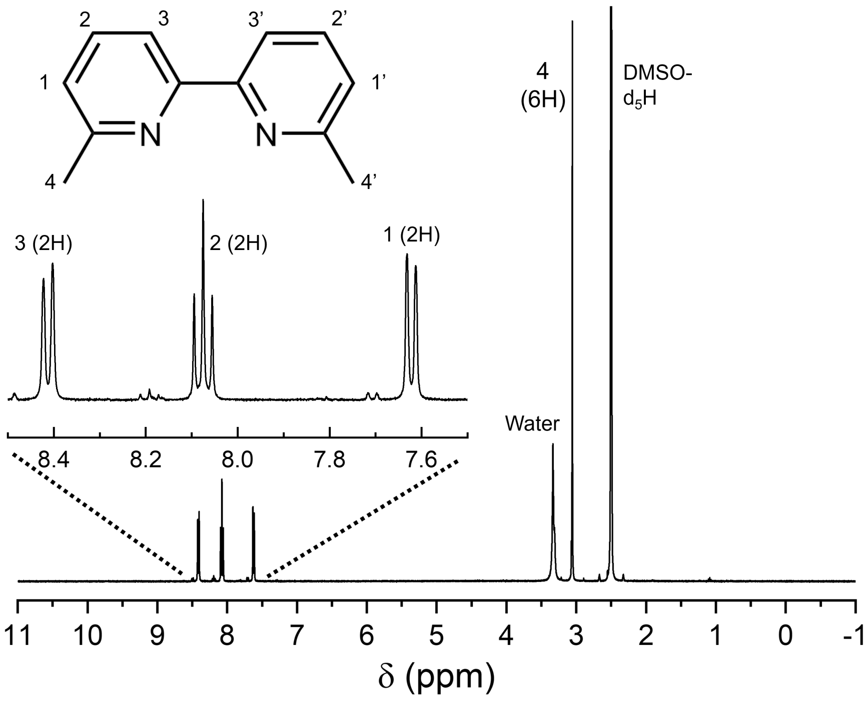

Figure S1: ^{1}H-NMR spectrum of Mn6dmb in DMSO-d_6, 400MHz, δ (ppm): 8.41 (d, 2H), 8.08 (t, 2H), 7.62 (d, 2H), 3.06 (s, 6H).

1.3 Synthesis of Mn4ditert

A 100 mL brown-glass round-bottom flask was equipped with a magnetic stir bar. $Mn(CO)_5Br$ (273 mg, 1.01 mmol) and 4,4'-tert-butyl-2,2'-dipyridyl (275 mg, 1.01 mmol) were dissolved in degassed diethyl ether (Et_2O, 30 mL, degassed with N_2 for 15 min.) and covered with aluminum foil. The reaction mixture was heated to reflux for 5 h under nitrogen atmosphere. After cooling to room temperature (23°C), the mixture was filtered through a Büchner funnel. The residue was washed with degassed Et_2O (2 × 10 mL). The product was obtained as an orange solid (291 mg, 0.597 mmol, 59.1%) without further purification.

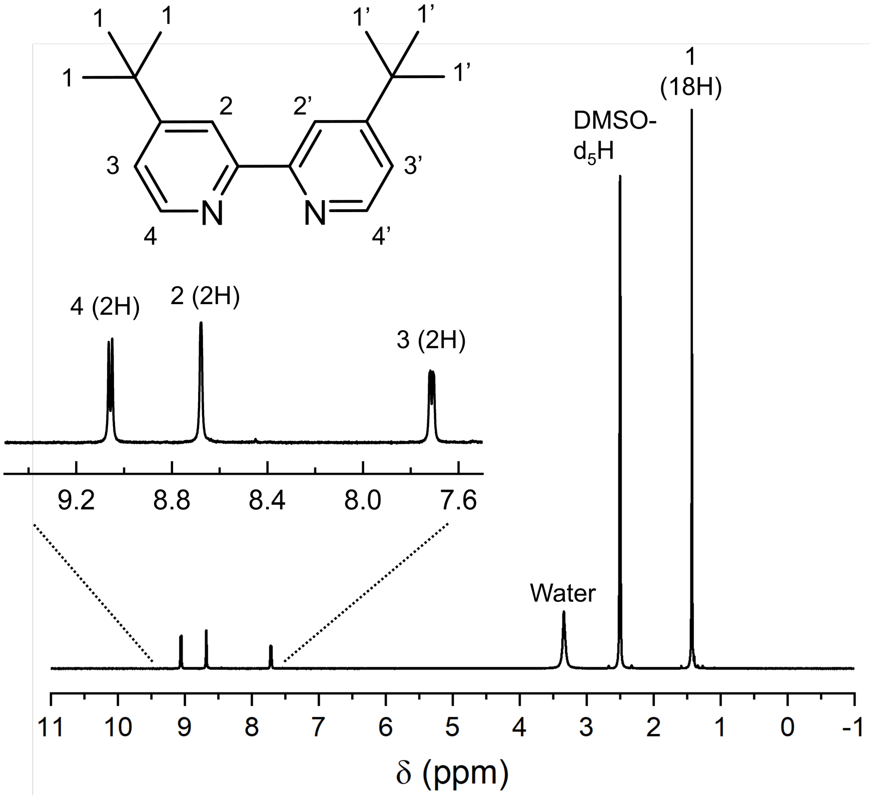

Figure S2: ^{1}H-NMR spectrum of Mn4ditert in DMSO-d_6, 400MHz, δ (ppm): 9.06 (dd, 2H), 8.68 (d, 2H), 7.71 (dd, 2H), 1.43 (s, 18H).

1.4 Synthesis of Mn4dicarb

Similarly to the synthesis above, 131.7 mg of $Mn(CO)_5Br$ (0.479 mmol) and 140.8 mg of dimethyl 2,2'-bipyridine-4,4'-dicarboxylate (0.517 mmol) were refluxed in Et_2O for 4 hours

under N_2 atmosphere in darkness. 197.3 mg of product was obtained (0.402 mmol, 83.9 % yield).

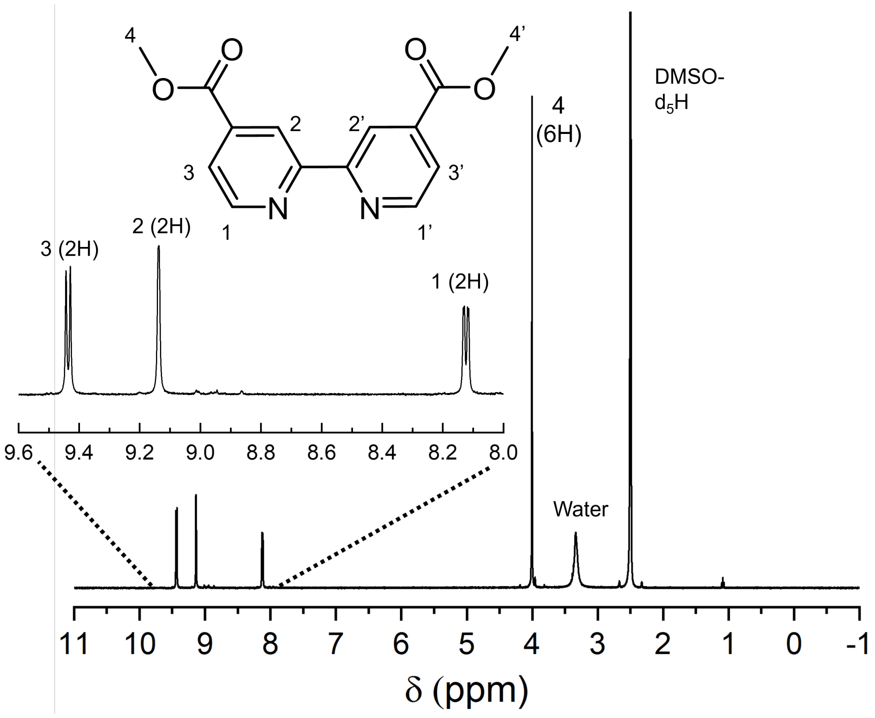

Figure S3: ^{1}H-NMR spectrum of Mn4dicarb in DMSO-d_6, 400MHz, δ (ppm): 9.44 (d, 2H), 9.14 (s, 2H), 8.12 (d, 2H), 4.00 (s, 6H).

1.5 Synthesis of Mn6mesb

1.5.1 6,6'-dimesityl-2.2'-dipyridine (6mesbpy)

The ligand, 6,6'-dimesityl-2.2'-dipyridine (6mesbpy) was synthesized via Suzuki-Miyaura cross-coupling using the known protocol[4,5] with a few modifications that are discussed next.

817.0 mg (2.60 mmol) of 6,6'-dibromo-2,2'-bipyridyl and 1070.5 mg (6.53 mmol) of 2,4,6-trimethylphenylboronic acid were put into 250 mL round-bottom flask. 40 mL of 2 M Na_2CO_3 aqueous solution was added alongside 20 mL of methanol, and 135 mL of toluene. This solution was bubbled for ∼10 min. with N_2, after which 32 mg of $Pd(PPh_3)_4$ catalyst was added. The oil bath was heated to 120 degrees with continuous stirring and reflux was maintained for 3 days (72 hours). After cooling the biphasic solution to room temperature,

organic and aqueous layers were separated by dropping funnel. Organic layer was washed twice with brine (saturated aqueous solution of NaCl). The latter was then transferred to another 250 mL round-bottom flask and solvent was evaporated in a rotary evaporator with 50°C water bath and ~70 mbar pressure. The obtained solid was then dissolved in ~7 mL $CHCl_3$ by slightly warming it up in the water bath and filtered. A significant amount of cold methanol (~80 mL) was added to the filtrate to precipitate a white solid as described by Kubiak et al.[5] The flask was stored in a freezer at -20°C overnight. After filtration of the white crystalline precipitate on the next day, it was dried under vacuum overnight to obtain 806.6 mg of the product (2.05 mmol, 79 % yield).

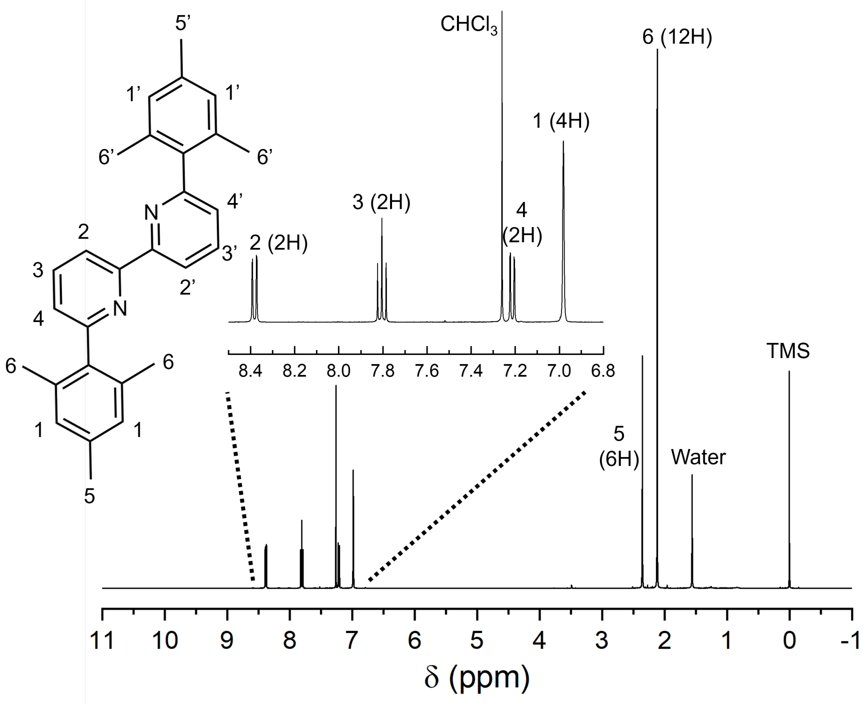

Figure S4: ^{1}H-NMR spectrum of Mn6mesb in $CDCl_3$, 400MHz, δ (ppm): 8.38 (d, 2H), 7.80 (t, 2H), 7.21 (d, 2H), 6.98 (s, 4H), 2.28 (s, 6H), 2.18 (s, 6H), 1.93 (s, 6H).

1.5.2 Synthesis of Mn6mesb

205 mg (0.746 mmol) of $Mn(CO)_5Br$ and 281.3 mg (0.717 mmol) were added to a brown-glass 100 mL round-bottom flask. Then 54 mL diethyl ether was added. Solution was refluxed under ambient air for 2 hrs. After ~1 hr the mixture became noticeably murky. After 2 hours

the heating was switched off and the flask was cooled to room temperature and then filtered. Washed with cold diethyl ether. After drying it under vacuum, 170 mg (0.278 mmol) of the complex was obtained (38.8 % yield).

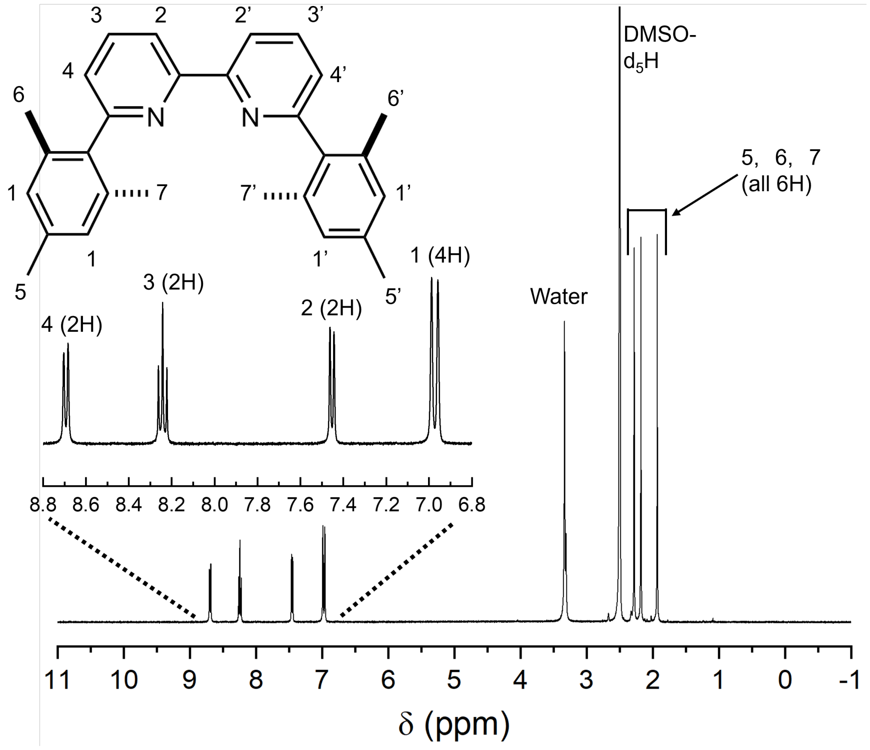

Figure S5: ^{1}H-NMR spectrum of Mn6mesb in DMSO-d_6, 400MHz, δ (ppm): 8.69 (d, 2H), 8.24 (t, 2H), 7.45 (d, 2H), 6.97 (d, 4H), 2.28 (s, 6H), 2.18 (s, 6H), 1.93 (s, 6H).

2 Steady-state and Control Experiments

The UV-Vis absorption spectra of the four pre-catalysts is presented in Fig. S6. A bathochromic shift of the HOMO-LUMO transition is evident in the case of Mn4dicarb due to the presence of electron-withdrawing groups on the bipyridine ligand, i.e., the methyl ester groups (see Fig. S6, magenta trace). The Mn4dicarb MLCT band is red-shifted with respect to the three other versions of the pre-catalyst and almost coincides with the PS spectrum (Fig. S6, blue trace).

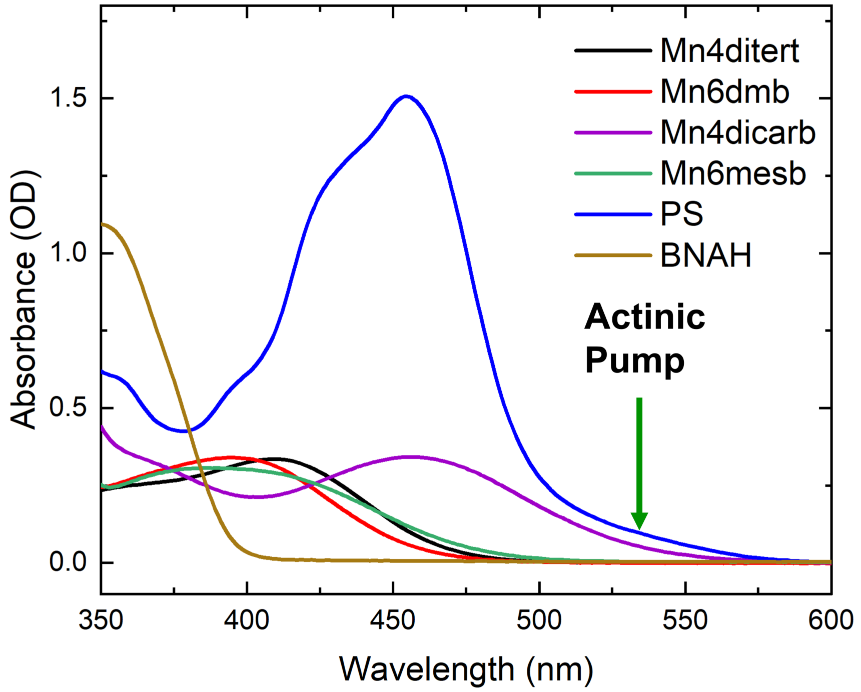

Figure S6: UV-Vis spectra of 10mM Mn4ditert, Mn6dmb, Mn4dicarb, Mn6mesb, BNAH, and PS in DMSO.

The electron-withdrawing groups also affect the IR spectra, although to a lesser extent. The FTIR spectrum of the Mn4dicarb resting state is characterized by slightly higher carbonyl frequencies, as compared to the other the pre-catalysts (see Fig. S7). This blue-shift is due to the reduced back-bonding effect in Mn4dicarb.

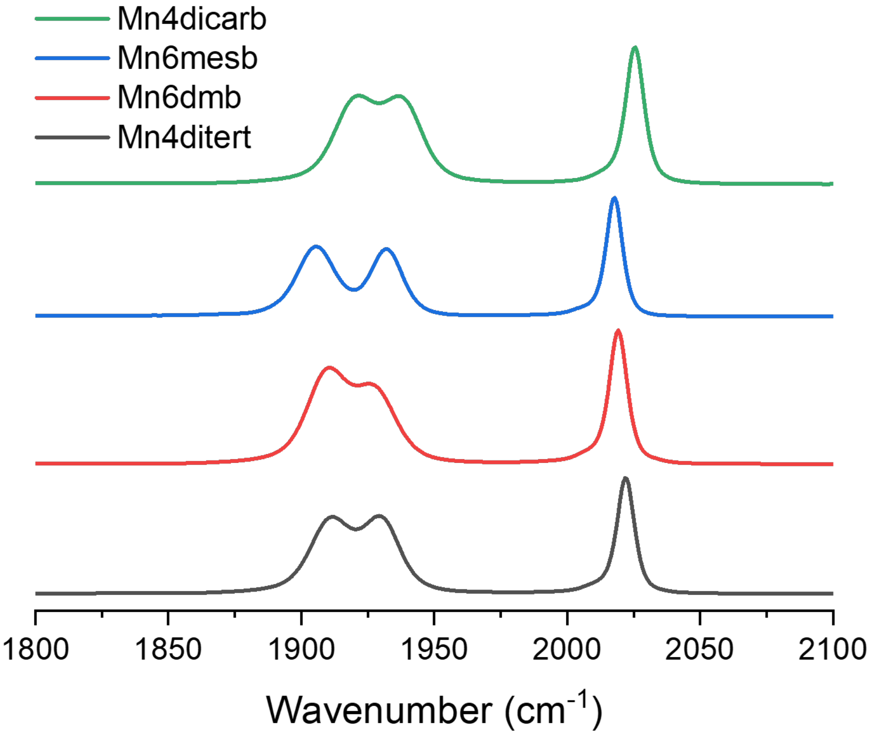

Figure S7: FTIR spectra of 10mM Mn4ditert, Mn6dmb, Mn4dicarb, and Mn6mesb in DMSO.

2.1 Solvolysis Experiments

Solvolysis of Mn6dmb and Mn4ditert is presented in Figures S8 and S9, respectively. As can be seen, the rate of exchange of axial Br^- ligand by solvent (DMSO) is more than 2x faster in the case of Mn6dmb. We propose two reasons contributing to this difference; firstly, bpy ring bending in Mn6dmb makes Bromide more labile (DFT-calculated Mn-Br bond length is also longer in the case of Mn6dmb), and secondly, the tert-butyl groups in Mn4ditert may be a steric hindrance, delaying formation of desired solvent cage configuration for the ligand's release.

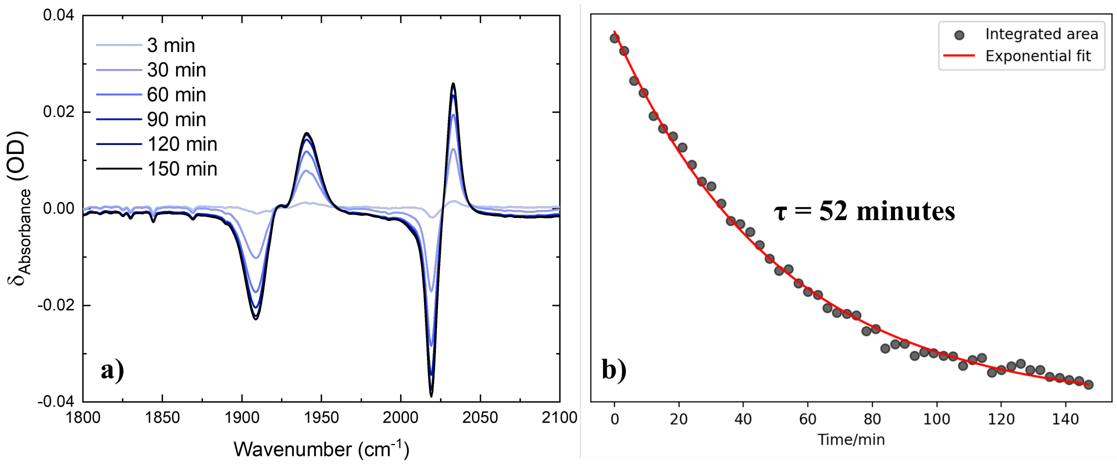

Figure S8: FTIR difference spectra of 10mM Mn6dmb in DMSO showing solvolysis (a), respective change in integrated peak area over 2.5 hours fit with exponential decay (b)

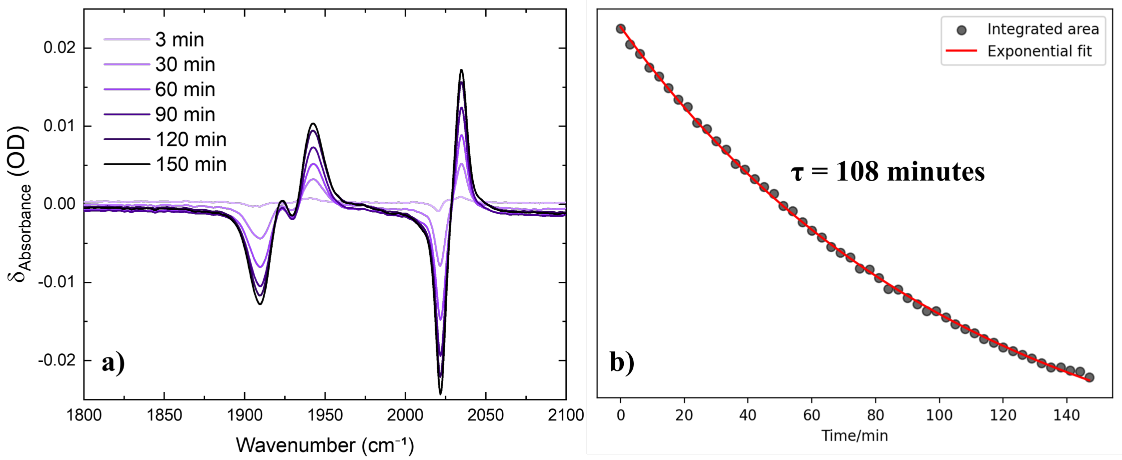

Figure S9: FTIR difference spectra of 10mM Mn4ditert in DMSO showing solvolysis (a), respective change in integrated peak area over 2.5 hours fit with exponential decay (b)

3 TRIR

A time window from ∼1 ns to ∼320 ms was covered using a single-VIS-pump-multiple-IR-probe arrangement. A visible pump pulse centered at 532 nm (6 μJ, 180 μm spot size, 5 ns duration) was generated with a diode laser (CNI) operated at 2 Hz rep rate, followed by multiple IR probe pulses from a Yb-doped fiber laser system at 100 kHz (Tangerine, Amplitude, France) pumping an optical parametric amplifier (OPA) (Twin STARZZ, Fastlite, France). The detector was a 2x32 MCT array with ∼6 cm^{-1} spectral resolution per pixel.[6]

The sample was measured in a home-built flow-cell consisting of two CaF_2 windows (thickness of 2 mm) with a 120 μm thick PTFE spacer between them and encapsulated in a metal frame. The sample exchange was facilitated by peristaltic pump in a stop-flow arrangement, see Ref.[7] for details. The sample reservoir was purged with Ar gas for the entire duration of the experiment. The Ar gas was pre-saturated with the solvent by bubbling it through a separate reservoir to avoid the loss of solvent in the main sample reservoir.

3.1 Photodegradation Side Reaction of Mn4dicarb

Mn(α-diimine)$(CO)_3$Br-type complexes are light-activated CO-releasing molecules. We have used excitation light at 532 nm to avoid direct excitation of the catalyst, but that was not sufficient for Mn4dicarb, which has a red-shifted optical absorption spectrum as compared to the other three compounds investigated here (see Fig. S6). Consequently, the TRIR spectra for Mn4dicarb in Fig. 7 in the main manuscript contain bleach and positive signals from the very start of the temporal window. These signals originate from the photodegradation of the catalyst. In order to understand this contribution to the reported spectra, we have done a control experiment with just the catalyst in DMSO, which is shown in Fig. S10. One needs to note that photodegradation happens to a lesser extent in the complete chemical system used during our studies, as PS absorbs a major portion of the excitation light.

K. White and colleagues[3,8] have suggested that photo-induced degradation involves equa-

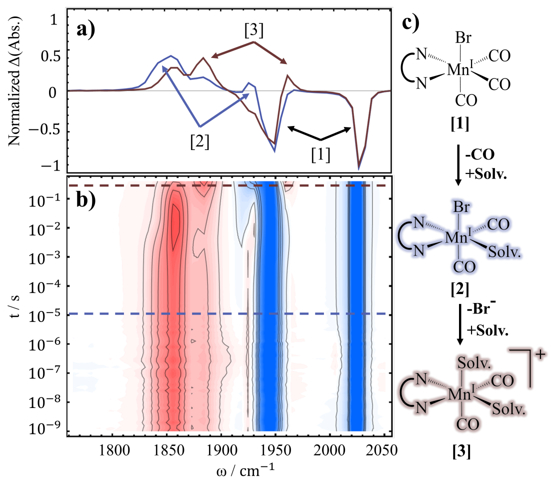

Figure S10: TRIR difference spectra of 10 mM Mn4dicarb in DMSO (b), accompanied by spectral cuts at selected delays (a) and reaction pathway (c).

torial carbonyl loss as the first step, followed by coordination of a solvent molecule to the empty site, and lastly by an exchange of axial Br^- with another solvent molecule. Although they were not able to directly observe these steps due to limitations of steady-state spectroscopy, they have predicted that reaction mechanism. Our results are in agreement with this scheme. While the 5 ns temporal resolution of the employed laser setup did not allow for observation of the initial CO-lost intermediate before solvent coordination, the rest of the steps were unambiguously resolved. The loss of CO reduces the number of bands to 2. Furthermore, the metal center share larger electron density with the two remaining carbonyls, which in turn is reflected into their reduced vibrational frequency. The first observed positive bands are hence assigned to $Mn^I(L)(CO)_2(DMSO)Br$ ([2] in Fig. S10). Additionally, contrary to the original catalyst, where Br^- exchange by solvent happens at a very slow rate (an hour timescale, see Sec. 2.1 in SI), the carbonyl loss also makes the halide more labile, because the Mn center becomes more electron-rich, and we observe the onset of exchange with a time constant of 200 ms, giving rise to a new pair of blue-shifted bands of $[Mn^I(L)(CO)_2(DMSO)_2]^+$ ([3] in Fig. S10). More details about this process is given in the section 4.2 below.

4 Data Analysis

A simple look at the TRIR data of the four complexes side by side, as shown in the Fig. S11, reveals multiple differences between them. A more systematic analysis of these spectra by the so-called "lifetime analysis"[9–13] follows.

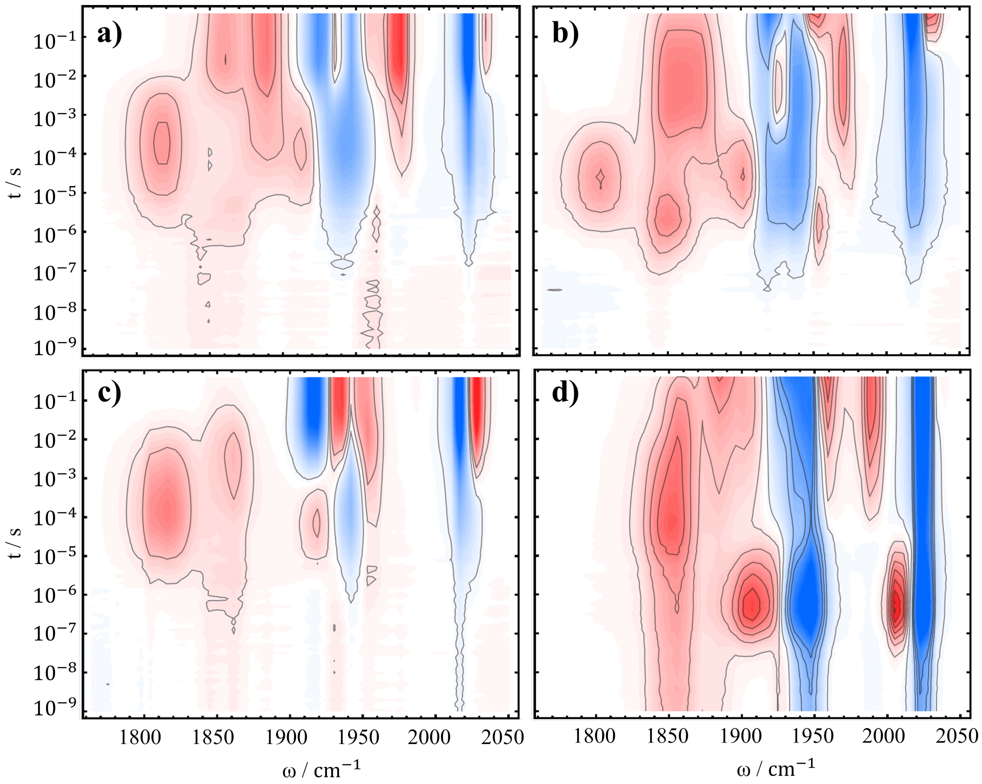

Figure S11: TRIR difference spectra of 10 mM PS, 50 mM BNAH, and 10 mM of the four complexes: Mn4ditert (a), Mn6dmb (b), Mn6mesb (c), and Mn4dicarb (d) in DMSO.

In the lifetime analysis, the time traces at each probe-wavelength ω_i are fit by:

$$f(t;\omega_i) = a_0(\omega_i) - \sum_k a(\omega_i,\tau_k)e^{-\frac{t}{\tau_k}} \tag{1}$$

which contains many exponential terms with time-constants τ_k that are distributed equally on a logarithmic time axis with 10 terms/decade. Only the amplitudes are treated as free fitting parameters, while the time constants, τ_k, are fixed. By summing up the fits at different wavelengths, ω_i, a lifetime density map $a(\omega_i,\tau_k)$ can be obtained. The fit is regularized with

a maximum entropy method,[9–11,13,14] to avoid over-fitting.

To condense the information of the lifetime density map, we finally averaged over all probe-frequency positions in a way that positive and negative amplitudes don't cancel out:

$$D(\tau_k) = \sqrt{\sum_i a^2(\omega_i, \tau_k)} \tag{2}$$

and call $D(\tau_k)$ the "dynamical content". The TRIR spectra of all the discussed samples, as well as their respective lifetime density maps and dynamical content plots can be viewed in Figures S12 to S19.

We can use Fig. S12 as an example in order to provide a quick guide on how to interpret the lifetime density maps. Fundamentally, lifetime density map highlights spectral, as well as temporal positions in the data where changes take place, and also shows the direction of this change, i.e., whether its a rise or depletion of certain species. The color code follows the same principle as in the TRIR figures. For example, one can see that the lifetime map in Fig. S12b shows a depletion of the bands of doubly-reduced complex, as well as parent complex, during the formation of dimer at 0.5 ms timescale, supporting our hypothesis of symproportionation.

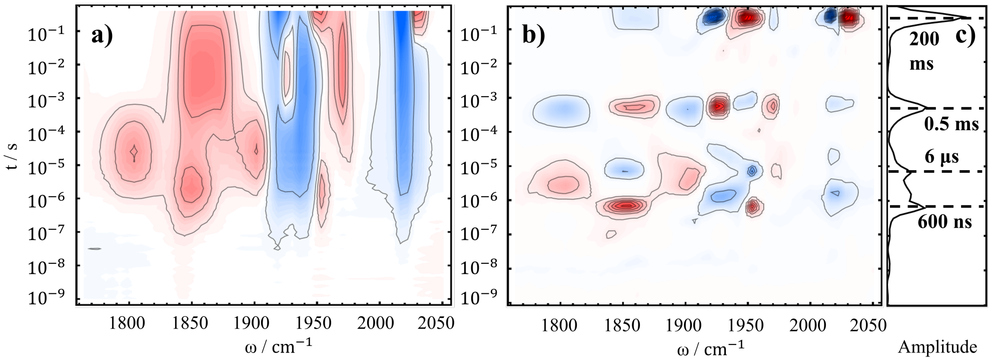

Figure S12: TRIR difference spectra of 10 mM Mn6dmb, 10 mM PS, and 50 mM BNAH in DMSO (a), respective lifetime distribution map (b), and dynamical content (c).

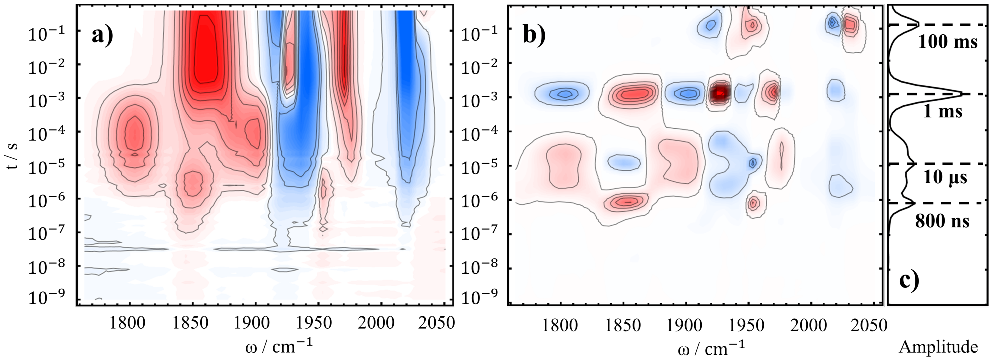

Figure S13: TRIR difference spectra of 10 mM Mn6dmb, 10 mM PS, and 50 mM BNAH, with 1 M TEOA in DMSO (a), respective lifetime distribution map (b), and dynamical content (c).

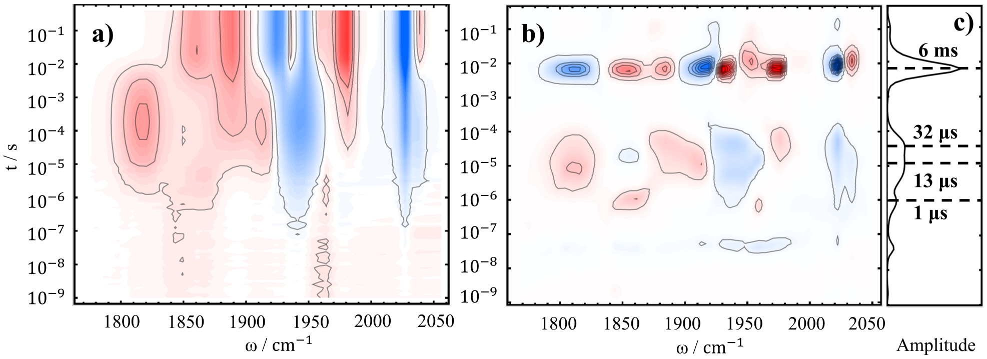

Figure S14: TRIR difference spectra of 10 mM Mn4ditert, 10 mM PS, and 50 mM BNAH in DMSO (a), respective lifetime distribution map (b), and dynamical content (c).

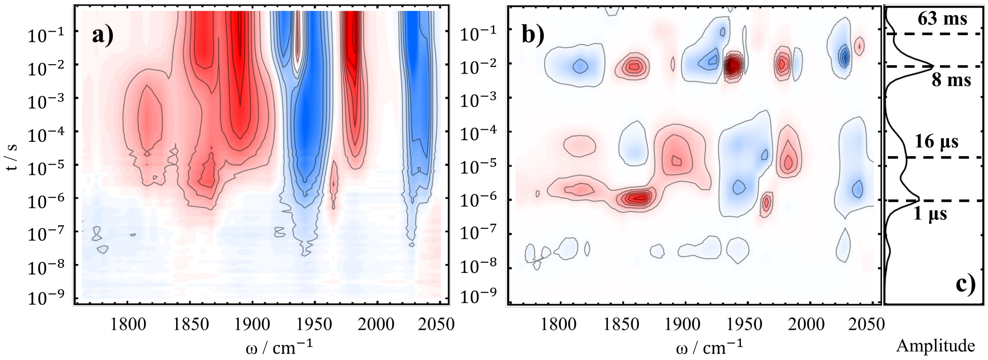

Figure S15: TRIR difference spectra of 10 mM Mn4ditert, 10 mM PS, and 50 mM BNAH, with 1 M TEOA in DMSO (a), respective lifetime distribution map (b), and dynamical content (c).

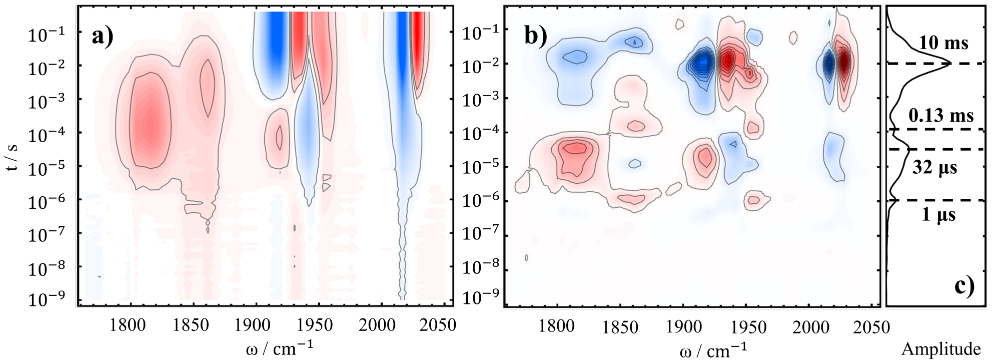

Figure S16: TRIR difference spectra of 10 mM Mn6mesb, 10 mM PS, and 50 mM BNAH in DMSO (a), respective lifetime distribution map (b), and dynamical content (c).

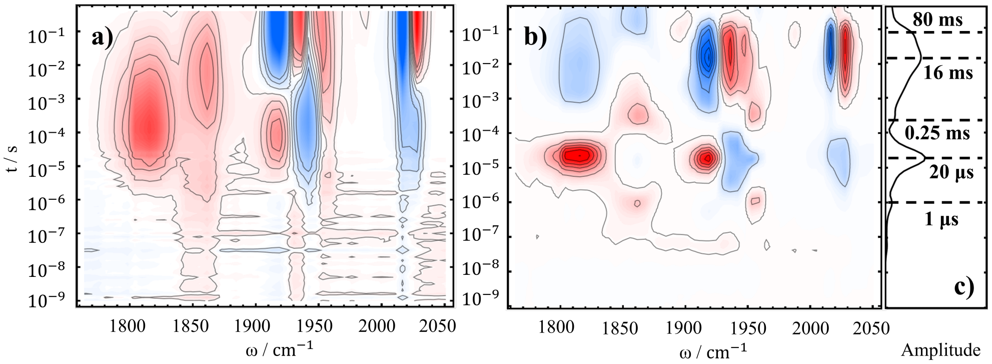

Figure S17: TRIR difference spectra of 10 mM Mn6mesb, 10 mM PS, and 50 mM BNAH, with 1 M TEOA in DMSO (a), respective lifetime distribution map (b), and dynamical content (c).

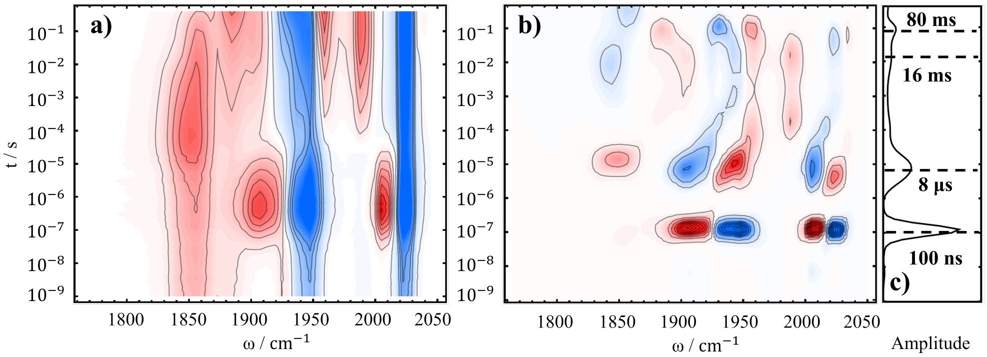

Figure S18: TRIR difference spectra of 10 mM Mn4dicarb, 10 mM PS, and 50 mM BNAH in DMSO (a), respective lifetime distribution map (b), and dynamical content (c).

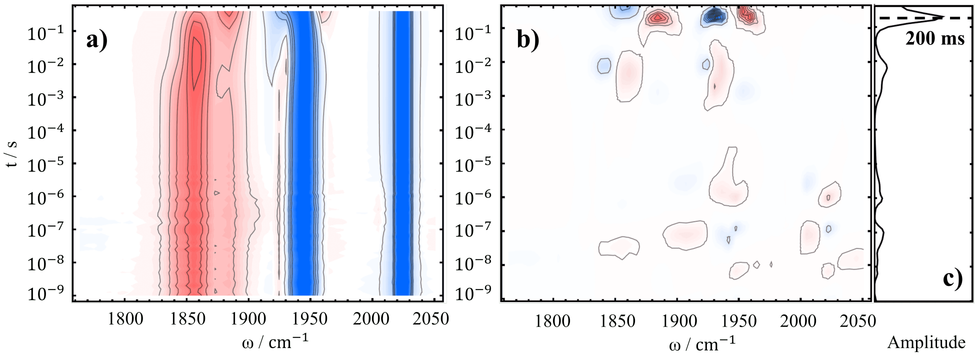

Figure S19: TRIR difference spectra of 10 mM Mn4dicarb in DMSO (a), respective lifetime distribution map (b), and dynamical content (c).

4.1 Gathering evidence for the elusive metal hydride

As has been discussed in the main manuscript in the analysis of Mn6dmb and Mn4ditert samples, a transient positive feature at $\sim$1980 cm^{-1} emerges on a timescale comparable to that of the doubly-reduced complex. One plausible explanation is the formation of an alternative dimer conformation, which then rearranges to the one observed in the millisecond regime. Two reasonable conformations can exist for Mn6dmb dimer species: pseudo-staggered (see Fig. S20a) and pseudo-eclipsed one (see Fig. S20b) with respect to equatorial carbonyls, which are in fact energetically equivalent according to the DFT calculations (see Table S1). However, the observed intensity profile of the dimer is more consistent with the pseudo-eclipsed one, as the $\sim$1975 cm^{-1} band is quite strong. The latter implies that the 1980 cm^{-1} feature cannot belong to the different, namely pseudo-staggered dimer conformation, because it exhibits a bathochromic shift instead of expected hypsochromic one. Moreover, both dimer conformers have three characteristic peaks (one that is a cluster of 4 modes), of which the middle one closest to the bleach is the most intense. This peak is missing from the observed trace along the 1980 cm^{-1} feature at $\sim$6 μs in Fig. S12. Furthermore, the DFT-calculated energies for Mn4ditert and Mn4dicarb dimers show that the pseudo-eclipsed version is the thermodynamically favored one and the two conformers are not energetically equivalent, as opposed to the Mn6dmb case.

A more plausible option is the transient formation of the metal hydride complex, Mn^{I}-H,

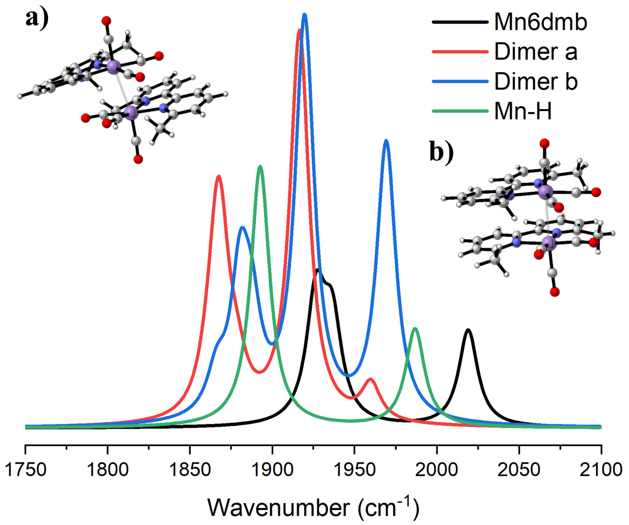

Figure S20: 2 possible dimer conformations: pseudo-staggered (a) and pseudo-eclipsed (b) with their respective calculated spectra. Mn-H represents the hydride complex spectrum. Parent complex, Mn6dmb, calculated spectrum is also shown as a reference. FWHM was set at 7 cm^{-1}.

generated by hydrogen atom transfer from $BNAH^{\bullet+}$ to Mn^0. The vibrational frequencies are quite close to the DFT-predicted modes of Mn^I-H (see Fig. S20, Mn-H trace), and the reaction is also thermodynamically feasible (ΔG_{rxn}^{298K} = -23.7 kcal/mol). Under basic conditions (1 M TEOA), lifetime distribution maps show enhanced intensity of this band, consistent with kinetic stabilization of the hydride through reduced bimolecular recombination rate, which is the major decay pathway of Mn^I-H species. One can inspect the lifetime distribution map (LDM) presented in Fig. S13b, which is a stark contrast from the LDM of normal condition spectra with regards to the discussed peak, which under basic media becomes more pronounced. The other two carbonyl modes of Mn^I-H are overlapped with the higher frequency mode of the doubly-reduced species near 1900 cm^{-1}, which obscures their resolution. It is also worth to point out that the highest frequency mode of Mn^I-H is observed slightly lower than the literature values,[15,16] however, the resolution of the MCT detector in our setup is ∼6 cm^{-1}, which could explain the relative mismatch.

Similar spectral behavior is observed for Mn4ditert, where the rise of the 1980 cm^{-1} band corresponds to an additional kinetic process in the dynamical content plots. Similar bands

to the Mn6dmb case above are clearly present (see Fig. S15). Notably, two time constants - $\sim$13 μs and $\sim$32 μs - can vaguely be distinguished during the formation of doubly-reduced catalyst, second of which could be associated to Mn^I-H species formation.

The lifetime distribution analysis of Mn6mesb spectra, shown in Fig. S16b, also follows the given interpretation. The path from initial 1e-reduced complex after Bromide loss, Mn^0, to 2e-reduced one, and then back to the Mn^0 radical is clearly present, followed by oxidation of metal center to Mn^I which preferably binds DMSO. In addition, there is faintly present new peaks in the LDM at approximately 1900 cm^{-1} and 1990 cm^{-1} in the millisecond regime. Their presence becomes significantly more observable under basic conditions with 1 M TEOA at 80 ms time constant (see Fig. S17b). These bands are close to the DFT-calculated frequency of Mn^I-H, and since it's related to the presence of base in solution the latter seems to be a plausible explanation. It is also evident the bands are very close to the positions of the Mn^I-H carbonyl modes that were discussed above for Mn6dmb and Mn4ditert systems.

4.2 Mn4dicarb photo-degradation disentangled

Lifetime distribution analysis of Mn4dicarb chemical system (Fig. S18b) as well as the control experiment with only the catalyst (Fig. S19b) are quite revealing. Noticeably, the first reduction happens significantly faster than in the case of 3 other catalysts, appearing at 100 ns regime, primarily due to the electron-withdrawing groups on Mn4dicarb. Another intriguing observation is the proof of regeneration of the parent complex at 8 μs that was inferred in the main manuscript based on the vertical trace along the high frequency bleach. Due to the delay of Bromide loss after the initial reduction of Mn4dicarb, the recombination events are more likely to happen than in the case of 3 other catalysts who rapidly release the halide upon reduction. This delayed loss affects second reduction and subsequent dimerization timescales. For comparison, Mn6dmb dimerization time constant is 0.5 ms, whereas for Mn4dicarb this process happens an order of magnitude slower ($\sim$16 ms). Following from

this, Mn^{I}-DMSO complex formation is also delayed. One can spot a faint hint of this process close to 80 ms at ∼2040 cm^{-1}. The positive feature extending before dimer formation could be related to a solvent-coordinated radical that may form before two Mn^{0} species combine into a dimer. The last process observed in the LDA of Mn4dicarb system is in fact convoluted with the photodegradation side reaction. Comparing the peaks from Fig. S18b to the ones in Fig. S19b (control experiment with only Mn4dicarb in DMSO), one can notice that they are identical. Since the photodegradation of catalyst was not completely suppressed it is not surprising to observe this reaction.

5 Computational Chemistry

DFT calculations have been performed with the Gaussian 16 package[17] using M06 functional[18] and Grimme's D3 model for dispersion forces.[19] All atoms were treated with the def2-SVP basis set, which belongs to the family of basis sets by Ahlrichs and coworkers.[20] Structure optimization and frequency calculation was done in a single step, with SuperFine integration grid option in Gaussian, as well as the solvation using implicit conductor-like polarizable continuum model (CPCM).[21,22] The minima were verified by the absence of imaginary frequencies in the normal mode analysis. An empirical scaling factor of 0.951 was used to scale harmonic frequencies and zero point vibrational energy. Single point energy calculations were performed for obtaining more accurate electronic energies using the same functional with the def2-TZVPPD basis set. Reaction energies were evaluated using electronic energies from the latter, corrected by the Gibbs free energy contributions from the standard thermochemical output of Gaussian.[23]

Table S1: The CO vibrational frequencies in cm^{-1} (IR intensities in brackets in km/mol) and Gibbs free energies (kcal/mol) of the relevant species calculated by DFT. All species are solvated in DMSO (CPCM), unless otherwise stated. The 3 vibrational modes are listed as symmetric, antisymmetric, symmmetric, from lower to higher frequency, with respect to the present symmetry element. In the case of thr dimer, 6 modes are listed in the same order. (ps)- and (pe)- stand for pseudo-staggered and pseudo-eclipsed conformations of dimer, respectively.

**Chemical species**	**CO stretch (s/as/s)**	**Gibbs Free Energy (kcal/mol)**
$Mn^{I}(6dmbpy)(CO)_3Br$	1927 (1918) / 1936 (1478) / 2019 (1502)	-2910863.5
$[Mn^{I}(6dmbpy^{\bullet-})(CO)_3Br]^-$	1900 (2081) / 1908 (1470) / 1997 (1672)	-2910929.6
$Mn^{0}(6dmbpy)(CO)_3$	1851 (2790) / 1856 (1971) / 1955 (1930)	-1295567.0
$[Mn^{0}(6dmbpy^{\bullet-})(CO)_3]^-$	1803 (2573) / 1816 (1707) / 1887 (4460)	-1295632.0
(ps)-$[Mn^{0}(6dmbpy)(CO)_3]_2$	1860 (191) / 1866 (1492) / 1868 (2149)<br>1879 (570) / 1917 (6052) / 1960 (577)	-2591137.7

(pe)-[Mn^0(6dmbpy)$(CO)_3]_2$	1862 (0) / 1866 (628) / 1881 (2096)<br>1888 (1238) / 1920 (6199) / 1969 (4308)	-2591137.7
Mn^0(6dmbpy)$(CO)_3$(DMSO)	1917 (1347) / 1922 (2068) / 2014 (1541)	-1642652.1
[Mn^I(6dmbpy)$(CO)_3$(DMSO)$]^+$	1940 (1717) / 1954 (1649) / 2038 (1256)	-1642582.0
Mn^I(6dmbpy)$(CO)_3$H	1892 (1725) / 1893 (2326) / 1987 (1521)	-1295934.4
Mer-Mn^I(6dmbpy)$(CO)_3$Br	1924 (1676) / 1946 (3233) / 2041 (202)	-2910850.2
Mn^I(4ditertbpy)$(CO)_3$Br	1931 (1815) / 1935 (1801) / 2023 (1482)	-3058731.1
[Mn^I(4ditertbpy$^{\bullet-}$)$(CO)_3$Br$]^-$	1905 (1946) / 1908 (1848) / 2001 (1620)	-3058796.2
Mn^0(4ditertbpy)$(CO)_3$	1860 (2884) / 1861 (2187) / 1961 (2511)	-1443431.3
[Mn^0(4ditertbpy$^{\bullet-}$)$(CO)_3]^-$	1817 (2547) / 1823 (2141) / 1900 (4590)	-1443498.8
(ps)-[Mn^0(4ditertbpy)$(CO)_3]_2$	1866 (421) / 1874 (1303) / 1875 (2190)<br>1888 (1154) / 1923 (5444) / 1968 (1149)	-2886875.9
(pe)-[Mn^0(4ditertbpy)$(CO)_3]_2$	1868 (220) / 1873 (1871) / 1881 (2417)<br>1888 (796) / 1925 (4551) / 1973 (4183)	-2886881.7
[Mn^I(4ditertbpy)$(CO)_3$(DMSO)$]^+$	1948 (1814) / 1951 (1765) / 2042 (1328)	-1790448.8
Mn^0(4ditert)$(CO)_3$(DMSO)	1920 (1968) / 1921 (1829) / 2015 (1615)	-1790520.6
Mn^I(4ditertbpy)$(CO)_3$H	1893 (2007) / 1900 (2206) / 1992 (1543)	-1443800.6
Mer-Mn^I(4ditertbpy)$(CO)_3$Br	1921 (1708) / 1949 (3379) / 2042 (255)	-3058721.4
Mn^I(6dmesbpy)$(CO)_3$Br	1925 (1611) / 1942 (1329) / 2016 (1427)	-3299219.6
[Mn^I(6dmesbpy$^{\bullet-}$)$(CO)_3$Br$]^-$	1900 (1757) / 1915 (1329) / 1996 (1552)	-3299286.9
Mn^0(6dmesbpy)$(CO)_3$	1856 (2371) / 1862 (1920) / 1952 (2059)	-1683921.2
[Mn^0(6dmesbpy$^{\bullet-}$)$(CO)_3]^-$	1827 (2144) / 1831 (1623) / 1900 (3992)	-1682829.2
Mn^0(6dmesbpy)$(CO)_3$(DMSO)	1910 (1993) / 1915 (1227) / 2000 (1443)	-2031008.2
[Mn^I(6dmesbpy)$(CO)_3$(DMSO)$]^+$	1943 (1500) / 1952 (1387) / 2032 (1194)	-2030938.1
Mn^I(6dmesbpy)$(CO)_3$H	1894 (1853) / 1899 (1679) / 1984 (1546)	-1684289.7
Mer-Mn^I(6dmesbpy)$(CO)_3$Br	1931 (1398) / 1942 (3154) / 2035 (262)	-3299204.5
Mn^I(4dcarbpy)$(CO)_3$Br	1940 (1766) / 1943 (1759) / 2026 (1635)	-3147492.1
[Mn^I(4dcarbpy$^{\bullet-}$)$(CO)_3$Br$]^-$	1914 (1905) / 1917 (1681) / 2008 (1616)	-3147570.3
Mn^0(4dcarbpy)$(CO)_3$	1903 (2740) / 1905 (1859) / 1978 (4419)	-1532195.7

$[Mn^0(4dcarbpy^{\bullet-})(CO)_3]^-$	1862 (2022) / 1862 (2351) / 1940 (4105)	-1532276.4
(ps)-$[Mn^0(4dcarbpy)(CO)_3]_2$	1887 (493) / 1893 (1448) / 1900 (1697)<br>1912 (920) / 1949 (4977) / 1981 (1078)	-3064405.1
(pe)-$[Mn^0(4dcarbpy)(CO)_3]_2$	1900 (134) / 1908 (1104) / 1917 (2961)<br>1920 (769) / 1958 (4726) / 1995 (5158)	-3064410.4
$Mn^0(4dcarbpy)(CO)_3(DMSO)$	1929 (1918) / 1930 (1659) / 2023 (1556)	-1879292.4
$[Mn^I(4dcarbpy)(CO)_3(DMSO)]^+$	1955 (1799) / 1959 (1706) / 2044 (1434)	-1879207.9
$Mn^I(4dcarbpy)(CO)_3H$	1906 (1954) / 1913 (2115) / 1995 (1939)	-1532563.5
Mer-$Mn^I(4dcarbpy)(CO)_3Br$	1932 (1724) / 1962 (3246) / 2046 (352)	-3147483.2
$Mn^I(4dcarbpy)(CO)_2Br(DMSO)$	1878 (1982) / 1942 (2432)	-3423463.9
$[Mn^I(4dcarbpy)(CO)_2(DMSO)_2]^+$	1886 (2036) / 1954 (2321)	-2155186.1
DMSO		-347097.2
Br^-		-1615372.1
BNAH		-431806.5
$BNAH^{\bullet+}$		-431686.3
$BNA^{\bullet}$		-431423.2
4,4'-BNA_2		-862865.7
BNA^+		-431342.5
H_2		-736.1
H^-		-410.8
CO_2		-118346.9
${HCO_2}^-$		-118786.7

5.1 Calculating Hydricities

The hydricities were calculated for all of the four hydride complexes reported here, according to a reported procedure with a slight modification.[24] The hydricity is a measure of hydride donating ability, see Eq. 3.

$$\text{M-H} \rightleftharpoons \text{M}^+ + \text{H}^- \tag{3}$$

If the hydricity of a metal hydride is lower than that of the formate (Eq. 4), then it is unlikely that the hydride complex will be able to reduce CO_2.

$$\text{HCO}_2{}^- \xrightarrow{\Delta G=+29.0 \text{ kcal/mol}} \text{CO}_2 + \text{H}^- \tag{4}$$

We have calculated the hydricities by considering it as solvolysis of the metal hydride. The following example is for Mn6dmb hydride complex (Eq. 5).

$$\text{Mn}^{\text{I}}(\text{6dmbpy})(\text{CO})_3\text{H} + \text{DMSO} \xrightarrow{\Delta G=+38.8 \text{ kcal/mol}} [\text{Mn}^{\text{I}}(\text{6dmbpy})(\text{CO})_3(\text{DMSO})]^+ + \text{H}^- \tag{5}$$

Similarly, calculations for the other three metal hydrides based on Mn4ditert, Mn6mesb, and Mn4dicarb result in 38.1 kcal/mol, 37.9 kcal/mol, and 41.9 kcal/mol, respectively. Whereas the reaction free energy of formate dissociation (Eq. 4) in DMSO (CPCM) was estimated at 29.0 kcal/mol, which suggests that $Mn^{I}(6dmbpy)(CO)_3H$, as well as the other hydrides reported here, are incapable of reducing CO_2.

5.2 Cartesian Coordinates for the reported species

5.2.1 Coordinates for Mn6dmb species

Table S3: Coordinates for Mn^{I}(6dmbpy)$(CO)_3$Br

Br	0.06417000	-0.44229300	2.23052700
C	3.41227300	1.79105300	-0.47103100
C	2.63224800	0.65247900	-0.70974000
N	1.30404600	0.66879700	-0.49360100
C	0.70580500	1.82965100	-0.14037800
C	1.43493700	2.98707800	0.11739700
C	2.81557600	2.95876200	-0.02623900
C	-0.76757000	1.81332600	-0.12996400
N	-1.34749000	0.63291500	-0.44485300
C	-1.51519900	2.96631100	0.09909100
C	-2.89517300	2.91110700	-0.03576400
C	-2.67948900	0.58622100	-0.64167900
C	-3.47495900	1.71931300	-0.43754600
Mn	0.00304500	-0.96229300	-0.27320600
C	-0.04092000	-1.32805900	-2.02537000
O	-0.06854200	-1.56286900	-3.15385700
C	-1.18020000	-2.24582700	0.17184000
C	1.24579600	-2.21350800	0.09808400
O	-1.86420300	-3.10667500	0.51481500
O	1.97469300	-3.05637300	0.38917900
C	-3.33436300	-0.67297400	-1.10118700
C	3.30333800	-0.57415700	-1.23037900
H	4.48960500	1.73898800	-0.64329200
H	0.93685000	3.90845100	0.41938300
H	3.41199700	3.85040900	0.18023100
H	-1.02896900	3.90469400	0.36680900
H	-3.50614200	3.79930200	0.14359600
H	-4.55244400	1.64621900	-0.60266900
H	-3.50958400	-1.36670000	-0.26437100
H	-2.73005200	-1.19909200	-1.85392200
H	-4.31549400	-0.44802000	-1.54151200
H	3.58206200	-1.25964900	-0.41458600
H	4.23241100	-0.30082900	-1.74926400
H	2.66364200	-1.12550100	-1.93343500

Table S4: Coordinates for $[Mn^{I}(6dmbpy^{\bullet -})(CO)_3Br]^-$

Br	-0.00006800	-0.61192500	2.28454500
C	3.44836100	1.79141300	-0.44193800
C	2.66362800	0.66182000	-0.65037200
N	1.33228000	0.65592800	-0.41855300
C	0.71084800	1.84518300	-0.06986300
C	1.48624100	3.00278000	0.20555200
C	2.84604800	2.97527400	0.03638200
C	-0.71049800	1.84530400	-0.06987100
N	-1.33212800	0.65615900	-0.41857600
C	-1.48570200	3.00302700	0.20555200
C	-2.84551400	2.97574500	0.03637700
C	-2.66347200	0.66226800	-0.65039400
C	-3.44802200	1.79198600	-0.44195100
Mn	-0.00007300	-0.93960000	-0.31905100
C	-0.00007100	-1.17983400	-2.07737900
O	-0.00011100	-1.31971000	-3.22572300
C	-1.20368300	-2.23060200	0.01007800
C	1.20326400	-2.23084500	0.01010700
O	-1.89371300	-3.11991200	0.27287200
O	1.89299400	-3.12035200	0.27302000
C	-3.33650200	-0.57544000	-1.15416500
C	3.33646800	-0.57598000	-1.15416800
H	4.52122900	1.74303500	-0.64429900
H	0.99366900	3.91975800	0.53610400
H	3.44990900	3.86363400	0.24340500
H	-0.99298100	3.91991800	0.53612100
H	-3.44923000	3.86420100	0.24341200
H	-4.52090000	1.74378300	-0.64430600
H	-3.50136000	-1.30872000	-0.34957400
H	-2.74528200	-1.07449400	-1.93727800
H	-4.32256700	-0.32811500	-1.57231300
H	3.50123300	-1.30929200	-0.34958800
H	4.32256200	-0.32879300	-1.57232700
H	2.74516500	-1.07493900	-1.93727900

Table S5: Coordinates for $Mn^0(6dmbpy)(CO)_3$

C	-3.44134400	1.63430100	0.22117000
C	-2.64314400	0.49853700	0.37443600
N	-1.31502700	0.54790600	0.12802100
C	-0.74340800	1.74119700	-0.16875300
C	-1.49966700	2.89622000	-0.36194000
C	-2.87438700	2.83517400	-0.18492800
C	0.72619500	1.74635000	-0.16563300
N	1.30527000	0.55504400	0.12363300
C	1.47449200	2.90884600	-0.34505600
C	2.84922200	2.85574900	-0.16651900
C	2.63396500	0.51174700	0.36871100
C	3.42437600	1.65435700	0.22630400
Mn	-0.00052100	-1.01892400	-0.14817300
C	0.04008800	-1.98439300	1.38798200
O	0.06946500	-2.63703900	2.34826500
C	1.18470000	-1.99424000	-1.06175700
C	-1.19152300	-2.03929500	-1.00234800
O	1.91592000	-2.65559900	-1.68070500
O	-1.92153800	-2.73266800	-1.58660400
C	3.25644700	-0.76602000	0.81665900
C	-3.25588200	-0.77874700	0.83679600
H	-4.51230700	1.55974100	0.42438500
H	-1.01951500	3.83793400	-0.63068500
H	-3.49246500	3.72387000	-0.33186800
H	0.98802000	3.85033000	-0.60299500
H	3.46113900	3.75040900	-0.30262700
H	4.49591100	1.58493300	0.42810000
H	3.39313900	-1.46765200	-0.02121000
H	2.62528900	-1.27294000	1.56226300
H	4.24652500	-0.57639400	1.25371500
H	-3.41821500	-1.47774300	0.00124300
H	-4.23327300	-0.58787000	1.30123900
H	-2.60448400	-1.28842200	1.56279000

Table S6: Coordinates for $[Mn^0(6dmbpy^{\bullet -})(CO)_3]^-$

C	-3.51980000	1.54969100	0.09059400
C	-2.72254400	0.42645600	0.16979500
N	-1.34841000	0.47808300	0.00798200
C	-0.80257100	1.75106100	-0.09954000
C	-1.59731000	2.91144000	-0.20838600
C	-2.96494600	2.82546700	-0.14118500
C	0.62293500	1.79522200	-0.07972700
N	1.23617400	0.56782500	0.03385600
C	1.36483700	2.99589600	-0.14713200
C	2.73338300	2.96305800	-0.05868600
C	2.60298400	0.55235900	0.17641200
C	3.35392600	1.71252000	0.12785500
Mn	-0.02378100	-1.01925400	-0.05614000
C	0.48198500	-1.84677800	1.41603000
O	0.82157500	-2.45973700	2.35617700
C	0.99292900	-1.78798800	-1.26916300
C	-1.12331100	-2.34897700	-0.46287600
O	1.66142900	-2.32264100	-2.07235400
O	-1.72406800	-3.30368300	-0.77872400
C	3.31175100	-0.74103600	0.41223500
C	-3.37607700	-0.87729900	0.47730100
H	-4.59907600	1.43251800	0.22507200
H	-1.11371300	3.88214400	-0.33763600
H	-3.59770500	3.71229500	-0.22674700
H	0.84395800	3.94885500	-0.26013700
H	3.32422200	3.88137500	-0.10895800
H	4.43887100	1.64236500	0.24393300
H	3.24444900	-1.41863100	-0.45280600
H	2.88273400	-1.28397400	1.26891200
H	4.37668200	-0.55651900	0.61385900
H	-3.41022900	-1.54821300	-0.39498500
H	-4.41074000	-0.71713600	0.81330800
H	-2.81977500	-1.41314200	1.26485800

Table S7: Coordinates for (ps)-[Mn^0(6dmbpy)$(CO)_3$]$_2$

C	1.46740500	2.69512400	-1.05446200
C	2.93740400	1.29627900	0.09210700
C	3.61874800	2.39396500	0.62707400
C	3.18828700	3.67306000	0.33044800
C	2.11356600	3.81710900	-0.54017200
C	0.36962100	2.90900400	-2.03852800
H	4.48073300	2.24646100	1.27729100
H	3.69559400	4.54546900	0.74836000
H	1.76532400	4.80819800	-0.84086700
C	3.38346400	-0.07593100	0.33230900
C	4.52382600	-0.35326900	1.09176000
C	4.93877000	-1.66257800	1.23978100
H	5.08354100	0.45314200	1.56503200
C	3.10143500	-2.33163900	-0.17984800
C	4.22734800	-2.65446900	0.57459500
H	5.81848600	-1.90658700	1.83968600
C	2.43072300	-3.42291000	-0.94227100
H	4.54756800	-3.69799200	0.62060600
N	1.84038100	1.43645600	-0.69511200
N	2.64561100	-1.05248800	-0.25416800
Mn	0.93900400	-0.36674300	-1.21775800
C	1.67863700	-0.44372400	-2.82357100
C	-3.10139100	-2.33168200	0.17963500
C	-3.38358700	-0.07591300	-0.33217400
C	-4.52410400	-0.35319000	-1.09142200
C	-4.93904400	-1.66248900	-1.23951900
C	-4.22743800	-2.65444900	-0.57463200
H	-5.08394700	0.45326100	-1.56447100
H	-5.81888700	-1.90644500	-1.83926000
H	-4.54762000	-3.69797900	-0.62074200
C	-2.93748400	1.29627600	-0.09194700
C	-3.61884200	2.39400000	-0.62682400
C	-3.18831200	3.67307300	-0.33021100
H	-4.48089000	2.24654200	-1.27696600
C	-1.46734900	2.69504500	1.05453200
C	-2.11351100	3.81706400	0.54032200
H	-3.69562700	4.54551000	-0.74805700
C	-0.36952600	2.90885400	2.03856800
H	-1.76520900	4.80813300	0.84101100
N	-2.64561100	-1.05251800	0.25406300
N	-1.84038100	1.43639500	0.69517500
Mn	-0.93898900	-0.36683500	1.21767000
C	-1.67864900	-0.44402300	2.82346000
C	0.04263100	-1.89514200	-1.42465100

O	-0.62674900	-2.80994400	-1.68847500
C	0.60122200	0.23480500	1.86721900
C	-0.04253200	-1.89520600	1.42441900
C	-0.60124000	0.23492000	-1.86722200
O	1.60289700	0.47105200	2.41279500
O	0.62698000	-2.80990600	1.68825700
O	-1.60295600	0.47154600	-2.41256100
C	-2.43048000	-3.42302900	0.94176500
H	-0.32295700	3.96754500	2.32823200
H	0.61295000	2.63872700	1.62479600
H	-0.52314200	2.30602700	2.94704500
H	-1.45678000	-3.68440000	0.50402200
H	-3.05271800	-4.32816200	0.92864400
H	-2.26195800	-3.13471100	1.99063600
H	3.05315600	-4.32791200	-0.92943600
H	2.26205200	-3.13432800	-1.99104300
H	1.45710600	-3.68460400	-0.50452600
H	-0.61289000	2.63900800	-1.62474200
H	0.52316900	2.30612500	-2.94697800
H	0.32316900	3.96769000	-2.32823300
O	2.10655600	-0.51215900	-3.89890400
O	-2.10650200	-0.51281800	3.89879400

Table S9: Coordinates for (pe)-[Mn^0(6dmbpy)$(CO)_3$]$_2$

C	1.88606500	1.03900300	2.25246600
C	-0.17356100	1.86959500	1.55241000
C	0.26298200	3.18929700	1.73113000
C	1.54890000	3.43107600	2.16678800
C	2.35779200	2.33376400	2.44189100
C	2.79643800	-0.08296700	2.61350100
H	-0.40728800	4.02485500	1.53109300
H	1.91213100	4.45156100	2.30738000
H	3.37688200	2.47127300	2.81083400
C	-1.55881500	1.57571100	1.18774800
C	-2.49190300	2.60017200	0.99271600
C	-3.81013700	2.28351000	0.72935700
H	-2.18936900	3.64507300	1.05322200
C	-3.20097700	-0.04939200	0.90206400
C	-4.16096900	0.94068600	0.69031200
H	-4.55371400	3.06714100	0.56766200
C	-3.65904000	-1.46757800	0.91546100
H	-5.19535100	0.63824200	0.51096000
N	0.62946200	0.79621300	1.77974700
N	-1.89579700	0.26025600	1.11769700
Mn	-0.31480300	-1.03624100	1.52815600
C	-0.74008400	-1.16528100	3.24224400
C	3.20103000	-0.04973800	-0.90220400
C	1.55908600	1.57563200	-1.18760900
C	2.49232000	2.59993200	-0.99243300
C	3.81050800	2.28304800	-0.72911500
C	4.16116000	0.94017300	-0.69029300
H	2.18994000	3.64488600	-1.05279400
H	4.55419400	3.06655300	-0.56730700
H	5.19550200	0.63755500	-0.51100400
C	0.17387800	1.86976300	-1.55223400
C	-0.26247100	3.18955200	-1.73078000
C	-1.54835300	3.43157900	-2.16640000
H	0.40792500	4.02498400	-1.53064400
C	-1.88585100	1.03956300	-2.25245100
C	-2.35739400	2.33442100	-2.44167700
H	-1.91144200	4.45213700	-2.30683800
C	-2.79634900	-0.08222500	-2.61375000
H	-3.37645900	2.47212900	-2.81061700
N	1.89588500	0.26011700	-1.11775500
N	-0.62929900	0.79652300	-1.77972300
Mn	0.31465100	-1.03609700	-1.52819500
C	0.73987200	-1.16518700	-3.24229200
C	-1.05649700	-2.60710300	1.11906900

O	-1.40001500	-3.70741600	0.96972100
C	-1.08297300	-2.12517800	-1.70827200
C	1.05608700	-2.60709400	-1.11912400
C	1.08259700	-2.12557800	1.70837100
O	-1.87238600	-2.96451900	-1.86788100
O	1.39942500	-3.70751000	-0.97012500
O	1.87181200	-2.96513400	1.86783300
C	3.65889600	-1.46798200	-0.91586300
H	-3.74275300	0.31160700	-3.00813300
H	-3.03509200	-0.71178000	-1.74502500
H	-2.34935600	-0.73187900	-3.38265300
H	3.21381600	-2.05755900	-0.09995400
H	4.75036800	-1.51381900	-0.80337400
H	3.39281600	-1.96380100	-1.86221500
H	-4.75050700	-1.51323800	0.80287200
H	-3.39311500	-1.96357800	1.86176700
H	-3.21397200	-2.05710500	0.09951000
H	3.03489800	-0.71249000	1.74467700
H	2.34948900	-0.73261400	3.38243700
H	3.74299100	0.31066700	3.00772800
O	-1.01264200	-1.29212000	4.36177900
O	1.01206700	-1.29219600	-4.36189600

Table S11: Coordinates for Mn^0(6dmbpy)$(CO)_3$(DMSO)

C	3.51169200	1.47494400	-0.31875900
C	2.67794000	0.37134800	-0.20424500
N	1.33803300	0.45474200	-0.40135100
C	0.78318700	1.69659700	-0.68011800
C	1.61580300	2.82786100	-0.87660000
C	2.97070500	2.72245600	-0.70123700
C	-0.63880600	1.77180200	-0.67562600
N	-1.31572700	0.60253800	-0.36456900
C	-1.35277900	2.97836400	-0.89113400
C	-2.70707700	3.01940800	-0.68249700
C	-2.64342600	0.67154400	-0.10158400
C	-3.36119200	1.85278600	-0.23186700
Mn	-0.08149100	-1.06754900	-0.56052500
C	-0.09197100	-0.85134300	-2.33426300
O	-0.09412500	-0.65039800	-3.46914100
C	-1.35222200	-2.34075700	-0.66037000
C	1.01980400	-2.48871100	-0.68869500
O	-2.06563300	-3.24164200	-0.77065200
O	1.60762800	-3.47499800	-0.80786300
C	-3.36767000	-0.55103000	0.36291200
C	3.29271700	-0.94280800	0.15621300
H	4.58152800	1.35730500	-0.13017200
H	1.17027800	3.78858000	-1.14219300
H	3.61870400	3.59275000	-0.83657400
H	-0.81816200	3.87781200	-1.20343900
H	-3.26564400	3.94655600	-0.83819400
H	-4.42750600	1.85879300	0.00690400
H	-2.75830900	-1.13357700	1.07073300
H	-3.62537000	-1.21573000	-0.47702000
H	-4.31035000	-0.27263800	0.85496300
H	2.68758400	-1.48640300	0.89783500
H	4.30079600	-0.79294100	0.56841800
H	3.39695500	-1.59294900	-0.72728600
O	-0.03376300	-1.16802000	1.53612200
S	-0.02484200	0.17340700	2.31829800
C	1.54397200	0.16679500	3.20115900
H	2.33390700	0.40226900	2.47351900
H	1.69716400	-0.83031200	3.64046600
H	1.51383900	0.94537600	3.97708500
C	-1.09858500	-0.19088300	3.71290800
H	-2.12318400	-0.26523200	3.32352800
H	-1.03564600	0.63023700	4.44117200
H	-0.78340400	-1.14642000	4.15714000

Table S12: Coordinates for [Mn^{I}(6dmbpy)$(CO)_3$(DMSO)]$^+$

C	-3.69697000	-1.60972500	0.45607100
C	-2.99475000	-0.46204100	0.06838600
N	-1.67625200	-0.52099200	-0.19477100
C	-1.04751700	-1.71821500	-0.17755600
C	-1.69265200	-2.88380500	0.22283400
C	-3.03701600	-2.82149900	0.56681000
C	0.33175800	-1.72704900	-0.69880700
N	0.81179100	-0.53420300	-1.12504700
C	1.06890900	-2.90111700	-0.82091400
C	2.31537300	-2.85285900	-1.43272700
C	1.98915100	-0.48900800	-1.77811200
C	2.76142400	-1.64500600	-1.94072500
Mn	-0.28000200	1.03175500	-0.28710000
C	-0.89384000	1.62859500	-1.87019200
O	-1.27873100	1.98226200	-2.89387200
C	1.02729100	2.27414700	-0.23243700
C	-1.22716700	2.23038200	0.69181200
O	1.80799600	3.11322200	-0.12344900
O	-1.74053700	3.01783700	1.35052200
C	2.49479000	0.79674600	-2.33671900
C	-3.73805000	0.82441100	-0.06314600
H	-4.76539700	-1.52944500	0.66845000
H	-1.16292700	-3.83614200	0.25273500
H	-3.56788400	-3.72007200	0.88898100
H	0.67422200	-3.84962700	-0.45608900
H	2.91691400	-3.75882400	-1.53371700
H	3.71527800	-1.57370100	-2.46790400
H	3.03247200	1.37814900	-1.57036700
H	1.68447900	1.42254300	-2.73554200
H	3.21065800	0.60016500	-3.14624700
H	-3.66636200	1.42792700	0.85517000
H	-4.80554500	0.62541500	-0.22933400
H	-3.36741600	1.43249500	-0.89995600
O	0.31082300	0.18892700	1.51343700
S	1.80798100	-0.09989000	1.82877400
C	1.69157000	-1.53774500	2.89208900
H	1.35036000	-2.37405000	2.26636400
H	0.96154200	-1.32083900	3.68523300
H	2.68532100	-1.75811000	3.30654500
C	2.23004900	1.13757700	3.05995300
H	2.24979900	2.10935300	2.54781500
H	3.22653900	0.91043900	3.46509700
H	1.46099100	1.12434500	3.84576800

Table S13: Coordinates for Mn^{I}(6dmbpy)$(CO)_3$H

C	-3.43480000	1.65320700	0.16980500
C	-2.64264300	0.50802500	0.31218900
N	-1.31678100	0.55086600	0.07560300
C	-0.73559800	1.74224900	-0.19643000
C	-1.47989800	2.90727900	-0.36868900
C	-2.85750300	2.85533700	-0.20635600
C	0.73571600	1.74220700	-0.19635800
N	1.31681900	0.55074600	0.07549800
C	1.48007700	2.90725100	-0.36828000
C	2.85766700	2.85520900	-0.20588400
C	2.64267800	0.50778600	0.31211400
C	3.43489400	1.65295800	0.17000200
Mn	-0.00007000	-1.04586900	-0.21544600
C	0.00016100	-1.66791600	1.49471200
O	0.00032000	-2.13672900	2.55266300
C	1.20571200	-2.17118800	-0.87824100
C	-1.20604800	-2.17132700	-0.87767000
O	1.93401500	-2.90320500	-1.40456600
O	-1.93446900	-2.90348600	-1.40363300
C	3.28274900	-0.76368700	0.75755600
C	-3.28277600	-0.76333100	0.75787400
H	-4.50787000	1.58107300	0.36222000
H	-0.99099200	3.85094500	-0.61253100
H	-3.46668600	3.75204400	-0.34126300
H	0.99122200	3.85100900	-0.61186600
H	3.46689700	3.75192600	-0.34052200
H	4.50795300	1.58073100	0.36243600
H	3.47714600	-1.44245400	-0.08703100
H	2.64503400	-1.30327300	1.47281200
H	4.24983200	-0.55332800	1.23488500
H	-3.47744500	-1.44213900	-0.08662100
H	-4.24973100	-0.55281200	1.23539200
H	-2.64496800	-1.30293800	1.47303000
H	-0.00017400	-0.55233800	-1.74626700

Table S14: Coordinates for Mer-Mn^{I}(6dmbpy)$(CO)_3$Br

C	-0.56539800	3.79542400	0.39857100
C	-0.88992300	2.45779700	0.66355100
N	-0.03860500	1.46793500	0.33427500
C	1.20011000	1.78617200	-0.11118000
C	1.56501500	3.09566100	-0.41274400
C	0.64896400	4.11457400	-0.18416000
C	2.16818500	0.67425000	-0.14575700
N	1.67995200	-0.54582500	0.18801000
C	3.52038300	0.88487900	-0.40282700
C	4.40643000	-0.17428300	-0.26320800
C	2.54305700	-1.56090900	0.39584400
C	3.91452800	-1.39353700	0.17193200
Mn	-0.37175100	-0.62524800	0.02052200
C	-0.71733100	-0.90756800	1.81219500
O	-0.97635600	-1.10735900	2.91285000
C	-0.59908900	-2.36724000	-0.31136200
C	-0.24616500	-0.33264500	-1.82767900
O	-0.84137300	-3.47416900	-0.53980900
O	-0.22896200	-0.18160700	-2.96172400
C	2.05342800	-2.88007000	0.88792800
C	-2.16953600	2.15599500	1.36325500
H	-1.28350500	4.57492500	0.66388000
H	2.55442100	3.32474900	-0.80957200
H	0.90159000	5.15052000	-0.42177700
H	3.88499300	1.87158000	-0.68867700
H	5.47173800	-0.03744600	-0.46173100
H	4.58384700	-2.23923900	0.34545100
H	1.61869900	-3.48205900	0.07528300
H	1.28374800	-2.76436100	1.66477300
H	2.88746300	-3.45791400	1.30894200
H	-2.94835500	1.82958000	0.65807100
H	-2.52512900	3.05050400	1.89335500
H	-2.04473200	1.33988400	2.08931300
Br	-2.89151000	-0.51343500	-0.51380100

5.2.2 Coordinates for Mn4ditert species

Table S15: Coordinates for $Mn^{I}(4ditertbpy)(CO)_3Br$

C	3.66115100	3.38593900	-1.37372700
C	3.00382300	3.72242900	-0.03100900
C	3.95656500	3.37147600	1.11654400
C	2.71835300	5.21969300	0.01186300
C	1.73241500	2.89919400	0.11291200
C	0.46140000	3.46045100	0.25395500
C	-0.64990300	2.63619700	0.37688900
N	-0.57566200	1.30448000	0.36770800
C	0.64102200	0.73512800	0.23288800
C	0.63981900	-0.73608200	0.23293100
N	-0.57775900	-1.30344500	0.36810500
Mn	-2.17119500	0.00182300	0.44172900
C	-2.18837400	0.00198500	2.23428300
O	-2.19794700	0.00203800	3.38671100
C	-3.43278300	1.28507600	0.32416100
O	-4.22878700	2.11063100	0.22055200
C	-3.43485700	-1.27942400	0.32433900
O	-4.23220000	-2.10368900	0.22079700
Br	-2.07400700	0.00133900	-2.10268900
C	-0.65414800	-2.63503300	0.37753500
C	0.45579200	-3.46112200	0.25449400
C	1.72766100	-2.90194200	0.11296900
C	2.99773600	-3.72721300	-0.03101500
C	3.65574500	-3.39169700	-1.37364200
C	3.95094300	-3.37790000	1.11666600
C	2.70982100	-5.22401000	0.01170600
C	1.79146000	-1.50435000	0.10632900
C	1.79397200	1.50150700	0.10656100
H	4.58134300	3.98050900	-1.49918200
H	2.98687200	3.62026200	-2.21439300
H	3.94047000	2.32262100	-1.44750300
H	4.88068700	3.96688200	1.03029300
H	4.24595300	2.30832600	1.11043200
H	3.49799800	3.59433300	2.09447700
H	3.66263300	5.77764400	-0.09294400
H	2.25881400	5.52467300	0.96676900
H	0.30851500	4.54040600	0.27081700
H	-1.64430100	3.07876000	0.48603800
H	-1.64923200	-3.07597500	0.48698600
H	0.30116700	-4.54082700	0.27164000
H	3.93689800	-2.32885600	-1.44732100
H	2.98115300	-3.62481200	-2.21439300
H	4.57494300	-3.98780700	-1.49906000
H	4.24198600	-2.31520000	1.11070200

H	4.87413200	-3.97475500	1.03043300
H	3.49193200	-3.60014100	2.09453100
H	2.04580800	-5.53997500	-0.80996500
H	2.24984600	-5.52835300	0.96660500
H	3.65318400	-5.78349500	-0.09320200
H	2.75620700	-1.00396600	-0.00136500
H	2.75795800	0.99954400	-0.00068000
H	2.05491500	5.53684700	-0.80981400

Table S17: Coordinates for $[Mn^{I}(4ditertbpy^{\bullet -})(CO)_3Br]^-$

C	3.65576500	3.43741100	-1.40471900
C	3.01219700	3.73579700	-0.04688300
C	3.99569500	3.38499700	1.07341000
C	2.71916500	5.23176400	0.02586100
C	1.74418600	2.89761400	0.10305000
C	0.45217000	3.47597000	0.24116300
C	-0.64710400	2.64263300	0.36343900
N	-0.58949800	1.30892900	0.35580300
C	0.65021900	0.70970000	0.22247500
C	0.65023200	-0.70968800	0.22248000
N	-0.58947100	-1.30894100	0.35581900
Mn	-2.15592200	-0.00001700	0.47515700
C	-2.10595600	-0.00002600	2.25129100
O	-2.05425000	-0.00001400	3.40646000
C	-3.41171500	1.28529800	0.39312500
O	-4.21260500	2.11472000	0.32011700
C	-3.41168000	-1.28536400	0.39311600
O	-4.21256400	-2.11479200	0.32009200
Br	-2.18349900	-0.00002500	-2.14106200
C	-0.64705400	-2.64264600	0.36343800
C	0.45223400	-3.47596200	0.24114500
C	1.74424000	-2.89758100	0.10304100
C	3.01226900	-3.73573900	-0.04687900
C	3.65593000	-3.43724500	-1.40464500
C	3.99568300	-3.38501300	1.07351200
C	2.71924500	-5.23171500	0.02573000
C	1.81136700	-1.51959100	0.09974100
C	1.81133800	1.51962500	0.09973800
H	4.56756900	4.04479600	-1.54069900
H	2.96250200	3.67582400	-2.22944200
H	3.94177800	2.37740300	-1.50052400
H	4.91340800	3.99166000	0.98225200
H	4.29393100	2.32455600	1.04604300
H	3.55284100	3.58593000	2.06389200
H	3.65765800	5.80111100	-0.07903400
H	2.26472500	5.51504900	0.99007700
H	0.29538500	4.55534300	0.25520000
H	-1.64380300	3.08699500	0.47172100
H	-1.64374400	-3.08702700	0.47172500
H	0.29546800	-4.55533800	0.25516100
H	3.94193600	-2.37722700	-1.50035700
H	2.96273400	-3.67561100	-2.22943700
H	4.56775400	-4.04460700	-1.54060100
H	4.29390900	-2.32456700	1.04624100

H	4.91340700	-3.99166300	0.98237800
H	3.55275600	-3.58602100	2.06394700
H	2.04249200	-5.55844500	-0.78134300
H	2.26476900	-5.51508400	0.98990200
H	3.65774700	-5.80104700	-0.07917300
H	2.78026200	-1.02203700	-0.00448600
H	2.78024100	1.02209000	-0.00449800
H	2.04237600	5.55855700	-0.78115600

Table S19: Coordinates for Mn^0(4ditertbpy)$(CO)_3$

C	3.41701900	-3.52755500	-1.25461900
C	3.73373100	-2.69359800	-0.00882000
C	3.37810700	-3.48888200	1.25170400
C	5.22899300	-2.39438600	0.00997800
C	2.90134000	-1.41981500	-0.04177800
C	3.45563300	-0.13295100	-0.05576000
C	2.63009100	0.97847900	-0.08915500
N	1.29040200	0.90510300	-0.11222900
C	0.73176800	-0.33079100	-0.09040200
C	-0.73146900	-0.33115400	-0.09039200
N	-1.29069900	0.90446200	-0.11217700
Mn	-0.00054800	2.48429300	-0.10477600
C	-0.00166900	2.98944300	1.62448300
O	-0.00247100	3.33456000	2.73264700
C	1.24619800	3.64983400	-0.63307400
O	2.06612400	4.40418500	-0.96641400
C	-1.24754800	3.64884600	-0.63471000
O	-2.06746400	4.40277400	-0.96903100
C	-2.63041300	0.97721900	-0.08901300
C	-3.45542800	-0.13460300	-0.05563000
C	-2.90052400	-1.42120400	-0.04174400
C	-3.73232000	-2.69537800	-0.00884700
C	-3.41523300	-3.52911300	-1.25470000
C	-3.37631200	-3.49056900	1.25162600
C	-5.22772100	-2.39686600	0.00998200
C	-1.50621200	-1.48827700	-0.05843400
C	1.50705900	-1.48754000	-0.05842700
H	4.01733800	-4.45275700	-1.25010800
H	3.65743100	-2.97011800	-2.17545800
H	2.35586500	-3.82061200	-1.30205700
H	3.97863800	-4.41292900	1.29478300
H	2.31618800	-3.78203100	1.27495700
H	3.58927400	-2.90288400	2.16193000
H	5.79411600	-3.34007600	0.03363300
H	5.52187400	-1.81236000	0.89964500
H	4.53491000	0.02645300	-0.04253800
H	3.06714200	1.98098400	-0.10334500
H	-3.06792200	1.97952700	-0.10313100
H	-4.53478000	0.02428500	-0.04234500
H	-2.35394400	-3.82167700	-1.30216500
H	-3.65591200	-2.97173100	-2.17550200
H	-4.01512500	-4.45459300	-1.25024000
H	-2.31425600	-3.78322500	1.27485100
H	-3.97641100	-4.41489800	1.29465800

H	-3.58774200	-2.90472100	2.16188800
H	-5.54898800	-1.84290200	-0.88775200
H	-5.52086700	-1.81502900	0.89968500
H	-5.79240000	-3.34282200	0.03358700
H	-1.00619300	-2.45966100	-0.04411800
H	1.00749200	-2.45915600	-0.04405300
H	5.54999300	-1.84032300	-0.88779100

Table S21: Coordinates for $[Mn^0(4ditertbpy^{\bullet -})(CO)_3]^-$

C	3.08379600	-3.81058800	-1.23748300
C	3.47536900	-2.99634900	-0.00060000
C	3.10339900	-3.77810100	1.26320500
C	4.98875100	-2.80103200	-0.01535100
C	2.72971000	-1.66442700	-0.01017700
C	3.38962900	-0.40333700	-0.01197400
C	2.65690200	0.75790700	-0.01704700
N	1.30147700	0.80363500	-0.02342500
C	0.63889800	-0.40328400	-0.01823200
C	-0.77539800	-0.26761800	-0.01768100
N	-1.20230800	1.05551000	-0.02277200
Mn	0.18607800	2.45357200	-0.01050100
C	1.00759200	3.14161600	1.39335200
O	1.59133300	3.61157700	2.29317900
C	1.14954000	3.22301500	-1.27791400
O	1.82387900	3.73734000	-2.08559500
C	-1.00546100	3.76601500	-0.05660100
O	-1.80257000	4.62167400	-0.08715300
C	-2.56010700	1.22469000	-0.01923000
C	-3.47533200	0.20580100	-0.01363600
C	-3.05183600	-1.15571800	-0.01005700
C	-4.01641500	-2.33793100	-0.00038800
C	-3.77497100	-3.20864000	-1.23745100
C	-3.78651900	-3.17564300	1.26159700
C	-5.47246500	-1.88130500	-0.01344200
C	-1.68628200	-1.34619400	-0.01187600
C	1.35057300	-1.62475200	-0.01259700
H	3.61952200	-4.77573100	-1.24862500
H	3.34039100	-3.26856100	-2.16365000
H	2.00384100	-4.02907000	-1.26289400
H	3.63917400	-4.74291500	1.29155100
H	2.02370800	-3.99445800	1.31072100
H	3.37433300	-3.21189300	2.17064900
H	5.49152600	-3.78238800	-0.01097300
H	5.34108200	-2.24523900	0.86978400
H	4.47866800	-0.32698800	-0.00878000
H	3.16536000	1.72882000	-0.01692500
H	-2.91352700	2.26040500	-0.02299600
H	-4.53451000	0.47309500	-0.01268400
H	-2.74812100	-3.60849600	-1.26514600
H	-3.93683400	-2.63098700	-2.16353200
H	-4.46769900	-4.06839800	-1.24711200
H	-2.75968400	-3.57385800	1.30871000
H	-4.47944400	-4.03493500	1.28820500

H	-3.95642500	-2.57342700	2.17044400
H	-5.70971300	-1.28953400	-0.91362000
H	-5.72061400	-1.27201700	0.87192600
H	-6.14010900	-2.75901100	-0.00854600
H	-1.27428700	-2.36091300	-0.00763300
H	0.77466000	-2.55571300	-0.00882700
H	5.32520200	-2.26057800	-0.91609700

Table S23: Coordinates for (ps)-[Mn^0(4ditertbpy)(CO)$_3$]$_2$

C	-3.41195900	4.57477600	1.15957600
C	-3.23957000	4.52973900	-0.36344200
C	-4.60470700	4.64274400	-1.04589100
C	-2.38181400	5.71665300	-0.79064600
C	-2.58442400	3.20225800	-0.71585500
C	-1.27155400	3.09839900	-1.19307600
C	-0.69645100	1.85351800	-1.37951900
N	-1.33662500	0.69833900	-1.13909600
C	-2.63166000	0.77313000	-0.73797100
C	-3.26780400	-0.52323100	-0.52686000
N	-2.44491900	-1.58564900	-0.71163000
Mn	-0.57736600	-1.16719200	-1.38988200
C	-1.01369700	-1.25917000	-3.10439900
O	-1.25071700	-1.33214000	-4.23706200
C	1.07055900	-0.58584800	-1.73315000
O	2.12383800	-0.19138800	-2.02881800
C	0.06059300	-2.83831900	-1.37788000
O	0.50308300	-3.91054300	-1.43807300
C	-2.95128900	-2.80811800	-0.48184000
C	-4.25239200	-3.02752300	-0.06576900
C	-5.12674800	-1.94495300	0.11678500
C	-6.57149200	-2.09957700	0.57252000
C	-6.75629600	-1.35236500	1.89749900
C	-7.50083700	-1.50538700	-0.49032900
C	-6.94682700	-3.56269200	0.78329800
C	-4.59633900	-0.68169100	-0.13397400
C	-3.25870000	1.99766500	-0.51235100
H	-3.85842800	5.53625100	1.46520600
H	-2.43670100	4.47315000	1.66708200
H	-4.06781400	3.76690500	1.52319400
H	-5.07647700	5.60514500	-0.78636100
H	-5.29757900	3.84453200	-0.73537500
H	-4.50385200	4.59861500	-2.14335800
H	-2.89918400	6.65675900	-0.53986500
H	-2.19359700	5.71837900	-1.87742100
H	-0.66181000	3.97972400	-1.40232600
H	0.34074600	1.78384300	-1.72406100
H	-6.55143700	-0.27410600	1.79872900
H	-6.08520600	-1.75511200	2.67481100
H	-7.79474700	-1.46285400	2.25278000
H	-7.31272200	-0.43225200	-0.65538100
H	-8.55250100	-1.61350500	-0.17554900
H	-7.37959900	-2.02410600	-1.45619200
H	-6.32705900	-4.03950700	1.56093800

H	-6.85002600	-4.15050500	-0.14477100
H	-7.99702200	-3.63180100	1.11019300
H	-5.21808100	0.20844800	-0.01029200
H	-4.28937600	2.00178800	-0.14923700
H	-1.40896300	5.73173300	-0.27121800
C	4.63990100	4.46709400	-0.12766500
C	3.19876500	4.57784800	0.35761900
C	3.17106600	5.44067700	1.62241200
C	2.36139600	5.24227000	-0.74137200
C	2.59357600	3.21607500	0.66140400
C	1.26983900	3.11351300	1.12375600
C	0.69580300	1.87491800	1.32159100
N	1.34469600	0.71548100	1.10348200
C	2.63917600	0.78936200	0.71108700
C	3.27911400	-0.50648000	0.51828700
N	2.45814800	-1.56884100	0.71609900
Mn	0.59041300	-1.14523600	1.38563000
C	1.02561900	-1.21183100	3.10141000
O	1.26321700	-1.26810100	4.23493800
C	-1.05894800	-0.56506500	1.72314500
O	-2.11379600	-0.17253800	2.01621400
C	-0.04340000	-2.81791500	1.39540300
O	-0.48329900	-3.89042700	1.46981500
C	2.96851300	-2.79329100	0.50462300
C	4.27073000	-3.01515900	0.09431000
C	5.14297200	-1.93272500	-0.10218400
C	6.58915700	-2.09039100	-0.55260400
C	6.77630100	-1.35944400	-1.88626300
C	7.51476100	-1.48126900	0.50505100
C	6.96770500	-3.55533000	-0.74418200
C	4.60917700	-0.66775300	0.12964700
C	3.27083200	2.01552300	0.47671000
H	5.04003900	5.47304000	-0.33319000
H	4.71682600	3.88457100	-1.06089600
H	5.29349100	3.99901500	0.62727700
H	3.59581700	6.43617500	1.40991900
H	3.76807300	4.98061200	2.42769300
H	2.14744800	5.59108900	2.00147100
H	2.79511200	6.21945300	-1.01261000
H	1.32218100	5.42031800	-0.41891800
H	0.65703300	4.00107300	1.30905900
H	-0.34392400	1.80491500	1.65760700
H	2.29332500	-3.63953100	0.66288900
H	4.59193900	-4.04596400	-0.06391700
H	6.56948300	-0.28038200	-1.80131000
H	6.10787000	-1.77283700	-2.66027800
H	7.81584900	-1.47260200	-2.23750600
H	7.32390600	-0.40656100	0.65617200

H	8.56748300	-1.59116500	0.19438500
H	7.39201300	-1.98810800	1.47701700
H	6.35035900	-4.04308000	-1.51696200
H	6.87014800	-4.13164400	0.19099500
H	8.01869500	-3.62657500	-1.06808400
H	5.22858100	0.22218700	-0.00619200
H	4.30516600	2.01254700	0.13008100
H	2.33342300	4.61606600	-1.65009300
H	-4.57063900	-4.05679000	0.10766600
H	-2.27402300	-3.65464900	-0.62906900

Table S25: Coordinates for (pe)-[Mn^0(4ditertbpy)(CO)$_3$]$_2$

C	1.76251500	4.38897900	-1.26573900
C	0.95061000	3.81385900	-2.43071200
C	-0.31184400	4.64848300	-2.62140700
C	1.77952200	3.89784700	-3.71568700
C	0.62774500	2.36079700	-2.11259800
C	1.62642600	1.37718100	-2.18590700
C	1.33012400	0.05626900	-1.91702700
N	0.10493000	-0.37077000	-1.56504200
C	-0.86655600	0.56671900	-1.44182700
C	-2.16497100	0.02778800	-1.05126900
N	-2.19823400	-1.32580000	-0.94516500
Mn	-0.47834300	-2.30746400	-1.41415900
C	-0.83894000	-2.44559300	-3.14368100
O	-1.04562900	-2.57545000	-4.27740200
C	1.17861300	-2.94687600	-1.59478100
O	2.25449500	-3.34316500	-1.78892500
C	-1.09185700	-3.95132200	-1.05958300
O	-1.49091400	-5.02285100	-0.86107300
C	-3.37565500	-1.88644000	-0.61865900
C	-4.52183300	-1.15759400	-0.35484700
C	-4.50489800	0.24380200	-0.43766700
C	-5.70469500	1.11631000	-0.09381900
C	-5.40961600	1.83005900	1.23080000
C	-5.93651400	2.15361600	-1.19495600
C	-6.97646700	0.28982500	0.07034100
C	-3.29198200	0.81586900	-0.81105900
C	-0.63117100	1.91943400	-1.71368200
H	2.04218900	5.43606700	-1.46995400
H	2.69095500	3.81780900	-1.09440600
H	1.17884200	4.36869300	-0.32998000
H	-0.03769300	5.68212200	-2.88646400
H	-0.92249000	4.70321000	-1.70597800
H	-0.94479600	4.24818700	-3.43106300
H	1.97257900	4.95406600	-3.96555100
H	1.24602900	3.43908800	-4.56509700
H	2.65335500	1.62652900	-2.46416300
H	2.11400700	-0.70241500	-2.00291400
H	-4.52657100	2.48710500	1.15198000
H	-5.22318200	1.10257700	2.03989300
H	-6.26678800	2.45805900	1.52671800
H	-5.08125200	2.83750400	-1.31653400
H	-6.81516600	2.77244300	-0.94819600
H	-6.12530400	1.66655300	-2.16634600
H	-6.90199800	-0.42411500	0.90692900

H	-7.21773300	-0.27487800	-0.84589100
H	-7.82655600	0.95700800	0.28607100
H	-3.19976000	1.90189700	-0.89360300
H	-1.46100400	2.62336500	-1.62820400
H	2.75955300	3.40369100	-3.61997700
C	0.31049600	4.64763300	2.62220400
C	-0.95175900	3.81274800	2.43126300
C	-1.78071500	3.89614500	3.71624700
C	-1.76374300	4.38805800	1.26646200
C	-0.62850900	2.35985800	2.11273900
C	-1.62690300	1.37593100	2.18586800
C	-1.33022300	0.05514900	1.91676000
N	-0.10491100	-0.37149300	1.56465600
C	0.86629200	0.56631800	1.44160600
C	2.16489100	0.02783800	1.05104800
N	2.19858200	-1.32574200	0.94485700
Mn	0.47900800	-2.30801900	1.41374200
C	0.83964600	-2.44579900	3.14329800
O	1.04641500	-2.57535000	4.27703500
C	-1.17775700	-2.94793800	1.59440700
O	-2.25350800	-3.34456500	1.78855500
C	1.09305600	-3.95166900	1.05906900
O	1.49244500	-5.02303900	0.86040300
C	3.37624200	-1.88598300	0.61851100
C	4.52223000	-1.15676000	0.35492100
C	4.50484000	0.24463100	0.43778500
C	5.70443800	1.11751900	0.09419700
C	5.40935300	1.83137100	-1.23035600
C	5.93580700	2.15474700	1.19551000
C	6.97647500	0.29142300	-0.06988300
C	3.29167800	0.81629000	0.81098700
C	0.63051500	1.91892300	1.71371400
H	0.03608600	5.68111900	2.88759400
H	0.92106000	4.70281300	1.70674200
H	0.94358100	4.24724400	3.43170800
H	-1.97397200	4.95224600	3.96644900
H	-1.24715900	3.43719300	4.56551000
H	-2.76065200	3.40184200	3.62033900
H	-2.04384100	5.43495300	1.47109700
H	-2.69195600	3.81663500	1.09472100
H	-2.65390000	1.62491800	2.46421000
H	-2.11388400	-0.70377000	2.00258200
H	3.39944900	-2.97679400	0.55271800
H	5.42719000	-1.70334800	0.08447400
H	4.52616600	2.48823500	-1.15156100
H	5.22314400	1.10395600	-2.03956400
H	6.26642600	2.45958500	-1.52610700
H	5.08032000	2.83835900	1.31705700

H	6.81431000	2.77386900	0.94896000
H	6.12460000	1.66760500	2.16686000
H	6.90235500	-0.42241500	-0.90658800
H	7.21775800	-0.27334500	0.84630500
H	7.82640300	0.95888900	-0.28537400
H	3.19904500	1.90228500	0.89349600
H	1.46019900	2.62305400	1.62846300
H	-1.17992200	4.36844500	0.33078200
H	-5.42656500	-1.70448200	-0.08424000
H	-3.39849100	-2.97726300	-0.55291000

Table S27: Coordinates for Mn^0(4ditert)$(CO)_3$(DMSO)

C	3.23528500	3.77736500	1.06570100
C	3.67748900	3.01500400	-0.18745000
C	3.42046800	3.87454800	-1.42872600
C	5.17762800	2.75437900	-0.08632400
C	2.87877700	1.71845100	-0.29275100
C	3.48955900	0.43512300	-0.27082800
C	2.69530300	-0.69109400	-0.37086700
N	1.36015900	-0.66568200	-0.47137600
C	0.73179500	0.56460800	-0.48691400
C	-0.68871700	0.52251100	-0.51256600
N	-1.24488100	-0.74231900	-0.56633300
Mn	0.10373700	-2.27051600	-0.56335400
C	0.18204000	-2.29813600	-2.34302400
O	0.23373200	-2.26744200	-3.49531200
C	1.42128700	-3.49343100	-0.39111300
O	2.26672200	-4.26819400	-0.26900800
C	-1.15198800	-3.56777200	-0.50987700
O	-1.95871500	-4.39081800	-0.47091100
C	-2.58327500	-0.84400700	-0.55617000
C	-3.43966000	0.23148700	-0.46022100
C	-2.90036900	1.55284300	-0.38159100
C	-3.83657600	2.74025200	-0.21700400
C	-4.60059000	2.57654600	1.10198500
C	-3.08572100	4.06672500	-0.17900100
C	-4.82693200	2.77713700	-1.38495800
C	-1.52992500	1.66641300	-0.42416500
C	1.50371900	1.75076900	-0.40804800
H	3.81286300	4.71216100	1.16953200
H	3.40113400	3.17244900	1.97347900
H	2.16723500	4.04642600	1.02995900
H	3.99933100	4.81246400	-1.37096700
H	2.35736700	4.14524900	-1.53242200
H	3.72535700	3.34318500	-2.34634400
H	5.71760300	3.71292300	-0.01463300
H	5.56344200	2.22203400	-0.97178600
H	4.56905000	0.30706600	-0.18268400
H	3.16610800	-1.68065200	-0.36412500
H	-2.99606600	-1.85756600	-0.61428000
H	-4.51696100	0.04912600	-0.44335200
H	-3.90185700	2.53478500	1.95579500
H	-5.20678000	1.65630300	1.11739300
H	-5.28258500	3.42970500	1.25996900
H	-2.38365100	4.11783300	0.66998000
H	-3.80136700	4.89766400	-0.06616700

H	-2.51596300	4.24210400	-1.10699800
H	-5.43469600	1.86008400	-1.44519800
H	-4.29750400	2.89615700	-2.34568200
H	-5.52003700	3.62816600	-1.27115500
H	-1.05570400	2.64788900	-0.36550300
H	0.98068600	2.71110800	-0.43066300
H	5.43359400	2.16305800	0.80864800
O	-0.02043600	-2.18292800	1.55495300
S	0.13452100	-0.85622600	2.33589900
C	0.87852700	-1.39951700	3.87807600
C	-1.52466900	-0.51551900	2.95089800
H	1.90199700	-1.71843600	3.63988700
H	0.29222300	-2.24262100	4.27191100
H	0.90020500	-0.55880200	4.58620100
H	-2.15000300	-0.26526500	2.08071300
H	-1.90534500	-1.41801200	3.45151200
H	-1.48352700	0.33887600	3.64225900

Table S29: Coordinates for $[Mn^{I}(4ditertbpy)(CO)_3(DMSO)]^{+}$

C	3.15981400	3.82269300	1.06478400
C	3.62454200	3.07155000	-0.18773300
C	3.33886300	3.91228400	-1.43657600
C	5.12783900	2.83697300	-0.08834400
C	2.85294300	1.76520500	-0.28791000
C	3.46144100	0.50838600	-0.27952300
C	2.68433200	-0.63863700	-0.37878900
N	1.35287700	-0.61070500	-0.46987400
C	0.73775800	0.59319000	-0.46651800
C	-0.73332000	0.53430200	-0.49670800
N	-1.25024900	-0.70613600	-0.59720700
Mn	0.11103800	-2.25158700	-0.58063200
C	0.23328700	-2.27854400	-2.37389900
O	0.31232400	-2.26398100	-3.52097400
C	1.43577700	-3.47353200	-0.39010600
O	2.28155100	-4.23755200	-0.24629700
C	-1.12998300	-3.57297300	-0.57894200
O	-1.92360000	-4.40340100	-0.56510200
C	-2.58263700	-0.84009600	-0.59598500
C	-3.44496200	0.23598100	-0.47657100
C	-2.93508100	1.53547100	-0.35197600
C	-3.87163300	2.72022300	-0.17833800
C	-4.66769400	2.51778400	1.11599600
C	-3.11401700	4.04025100	-0.08637500
C	-4.82627200	2.78244900	-1.37486200
C	-1.54568300	1.66009700	-0.37402000
C	1.45633800	1.77828300	-0.38758000
H	3.71772800	4.76886700	1.16031200
H	3.34183600	3.22631600	1.97445700
H	2.08723900	4.07221200	1.02979200
H	3.89630100	4.86195900	-1.38158100
H	2.27043200	4.16084000	-1.53954500
H	3.65599100	3.38370700	-2.35098700
H	5.64705000	3.80612500	-0.01882600
H	5.52314700	2.31361000	-0.97471700
H	4.54321600	0.39426500	-0.20069900
H	3.16450700	-1.62131600	-0.38137400
H	-2.98187700	-1.85381900	-0.68710400
H	-4.52041100	0.04313500	-0.47335100
H	-3.99452600	2.44938100	1.98736800
H	-5.28265000	1.60405600	1.08793800
H	-5.34626700	3.37219200	1.27517100
H	-2.43427700	4.06765700	0.78144300
H	-3.83117700	4.86780100	0.03310500

H	-2.52531500	4.24280400	-0.99654300
H	-5.44271100	1.87408200	-1.46648600
H	-4.27095400	2.91540600	-2.31832400
H	-5.51197100	3.63814400	-1.26014700
H	-1.08006400	2.64099600	-0.27900000
H	0.91889900	2.72880200	-0.39614600
H	5.39629100	2.25415700	0.80835800
O	-0.07263300	-2.18747400	1.48979800
S	0.08438900	-0.90160600	2.34306000
C	1.22498700	-1.40996200	3.62864800
C	-1.43884100	-0.85186100	3.29017900
H	2.19581400	-1.56709400	3.13899100
H	0.85714400	-2.34530000	4.07467000
H	1.30384900	-0.60805800	4.37618400
H	-2.24664800	-0.61307800	2.58342100
H	-1.60070300	-1.83970200	3.74566900
H	-1.36237900	-0.06271600	4.05184800

Table S31: Coordinates for $Mn^{I}(4ditertbpy)(CO)_3H$

C	3.40149300	-3.51835600	-1.27459700
C	3.72689200	-2.69619800	-0.02329000
C	3.37212500	-3.49933700	1.23239400
C	5.22331300	-2.40315000	-0.00787200
C	2.89988300	-1.41862800	-0.04309800
C	3.45702300	-0.13695800	-0.04704800
C	2.63158800	0.97907200	-0.06832200
N	1.29758800	0.90613600	-0.08817700
C	0.73417000	-0.32204100	-0.07560900
C	-0.73417600	-0.32202900	-0.07560900
N	-1.29757400	0.90615800	-0.08818000
Mn	0.00002000	2.50100700	-0.15561200
C	0.00001500	2.77858100	1.64404700
O	0.00001900	3.01562600	2.77558800
C	1.26581400	3.69120300	-0.54337000
O	2.09342000	4.43849400	-0.85762400
C	-1.26575700	3.69121200	-0.54339200
O	-2.09332000	4.43850400	-0.85775500
C	-2.63157300	0.97911600	-0.06832300
C	-3.45702700	-0.13690100	-0.04704400
C	-2.89990700	-1.41858000	-0.04309300
C	-3.72693700	-2.69613600	-0.02327800
C	-3.40155900	-3.51830300	-1.27458500
C	-3.37217700	-3.49927800	1.23240700
C	-5.22335200	-2.40306200	-0.00785100
C	-1.50379000	-1.48162300	-0.05649700
C	1.50376500	-1.48164800	-0.05649800
H	3.99790000	-4.44599300	-1.27974000
H	3.64081100	-2.95455000	-2.19181500
H	2.33901600	-3.80679600	-1.32037900
H	3.96885600	-4.42615400	1.26603100
H	2.30909900	-3.78830000	1.25731500
H	3.58916500	-2.92131300	2.14630900
H	5.78457100	-3.35129700	0.00567300
H	5.52202800	-1.82986400	0.88551200
H	4.53668600	0.01972000	-0.03344900
H	3.07182600	1.98038800	-0.07386100
H	-3.07179500	1.98043900	-0.07386300
H	-4.53668700	0.01979400	-0.03344100
H	-2.33908700	-3.80676300	-1.32037100
H	-3.64087200	-2.95449500	-2.19180200
H	-3.99798300	-4.44592900	-1.27972300
H	-2.30915600	-3.78825900	1.25732300
H	-3.96892300	-4.42608500	1.26604900

H	-3.58920200	-2.92124800	2.14632200
H	-5.54300000	-1.84286600	-0.90228500
H	-5.52205200	-1.82977000	0.88553300
H	-5.78462600	-3.35119900	0.00569700
H	-1.00188600	-2.45186900	-0.05025500
H	1.00184500	-2.45188600	-0.05026000
H	5.54296600	-1.84295800	-0.90230600
H	0.00001700	2.28326500	-1.75191200

Table S33: Coordinates for Mer-Mn^{I}(4ditertbpy)$(CO)_3$Br

C	-2.25555400	4.62158300	1.25387100
C	-1.37566300	4.58560000	-0.00004400
C	-2.25585300	4.62147000	-1.25375100
C	-0.47585700	5.81676800	-0.00020500
C	-0.56987300	3.29463700	-0.00006200
C	0.82616600	3.25625100	-0.00013600
C	1.48915100	2.03464900	-0.00011800
N	0.85071300	0.86437800	-0.00006000
C	-0.49945300	0.86981800	-0.00000200
C	-1.12330000	-0.46179200	-0.00003800
N	-0.25406900	-1.49851000	-0.00025100
Mn	1.72053900	-1.03728200	-0.00004300
C	1.83696600	-0.98242500	-1.85445500
O	1.97396500	-0.94184500	-2.99137000
C	2.32734800	-2.71621700	-0.00015200
O	2.72310900	-3.80356300	-0.00021300
C	-0.75929500	-2.73590600	-0.00040700
C	-2.12168100	-3.00314400	-0.00034400
C	-3.04100800	-1.95126800	-0.00005600
C	-4.54939800	-2.15100100	0.00007800
C	-5.13692700	-1.49515500	1.25405900
C	-5.13716700	-1.49491500	-1.25366900
C	-4.92577100	-3.62868900	-0.00002000
C	-2.49949100	-0.66220500	0.00009300
C	-1.22305800	2.05727600	0.00001700
H	-2.84077600	5.55603400	1.27395300
H	-1.64175200	4.58446300	2.16944200
H	-2.96917100	3.78275400	1.28832900
H	-2.84105700	5.55593300	-1.27379500
H	-2.96950000	3.78265700	-1.28794800
H	-1.64227100	4.58423500	-2.16946400
H	-1.09703400	6.72682500	-0.00021700
H	0.16829700	5.85677600	-0.89431700
H	1.42426500	4.16876000	-0.00022000
H	2.58351200	1.98166700	-0.00013300
H	-0.04276800	-3.56188200	-0.00056900
H	-2.44162300	-4.04618800	-0.00052900
H	-4.94248600	-0.41113700	1.28921600
H	-4.71854900	-1.94606300	2.16949100
H	-6.23035500	-1.63695400	1.27365400
H	-4.94273000	-0.41089100	-1.28864000
H	-6.23059800	-1.63671900	-1.27309600
H	-4.71894800	-1.94563800	-2.16926500
H	-4.54629800	-4.15094300	0.89398000

H	-4.54650400	-4.15077800	-0.89420500
H	-6.02299500	-3.72943000	0.00009100
H	-3.16076700	0.20709500	0.00032200
H	-2.31455700	2.01656700	0.00009600
H	0.16844800	5.85690100	0.89379000
C	1.83654000	-0.98260600	1.85445100
O	1.97322400	-0.94209600	2.99140400
Br	4.10751300	-0.12912600	0.00028700

5.2.3 Coordinates for Mn6mesb species

Table S35: Coordinates for $Mn^{I}(6dmesbpy)(CO)_3Br$

Mn	-0.00011200	-0.15573700	0.27769600
O	0.00569700	-1.93111600	-2.06720300
O	-1.79356200	-1.99233100	1.72444000
O	1.78539900	-1.99834100	1.72642600
N	1.33885800	1.28902100	-0.47659300
N	-1.33227200	1.28834300	-0.48870800
C	0.00227200	-1.19806100	-1.17560800
C	-1.18552100	-1.23382500	1.10757700
C	1.18034200	-1.23757600	1.10930600
C	2.68159900	1.21234500	-0.56400800
C	3.46317500	2.34063600	-0.83091700
H	4.54842800	2.22455800	-0.89255700
C	2.85659100	3.57203100	-1.01671400
H	3.44891400	4.46811300	-1.21449200
C	1.47241500	3.63718800	-0.97046000
H	0.96862200	4.58855100	-1.13899800
C	0.74102100	2.47833200	-0.71269900
C	-0.73170600	2.47399300	-0.73761300
C	-1.45816100	3.62078800	-1.05614800
H	-0.95141500	4.56595000	-1.24867600
C	-2.84027600	3.55015600	-1.13946200
H	-3.42847800	4.43678200	-1.38608000
C	-3.44923500	2.32473100	-0.92533000
H	-4.53269900	2.20412900	-1.00729400
C	-2.67261300	1.20696000	-0.60464000
C	3.38542800	-0.08827900	-0.43316500
C	3.43623800	-0.95331800	-1.54229500
C	4.13099200	-2.15801900	-1.41730900
H	4.15994800	-2.84319400	-2.27324100
C	4.81301300	-2.49733400	-0.24552400
C	4.79972000	-1.58699700	0.81377500
H	5.33923200	-1.83112700	1.73687700
C	4.09501900	-0.38485700	0.74369200
C	2.83842000	-0.56096800	-2.86028400
H	1.85180300	-0.08425300	-2.76112500
H	2.72996100	-1.42981200	-3.52604900
H	3.48719600	0.17148000	-3.37279100
C	5.52488400	-3.80852600	-0.12246300
H	4.85749700	-4.58197500	0.29570500
H	6.39313100	-3.73729300	0.55041300
H	5.87098200	-4.17739200	-1.10005400
C	4.06174400	0.54614000	1.91781400
H	4.58737900	1.49299700	1.70653500
H	4.54010600	0.09180400	2.79782300

H	3.02693700	0.81913100	2.19319900
C	-3.38234900	-0.08721600	-0.44268800
C	-3.44452700	-0.97676400	-1.53116100
C	-4.14893100	-2.17258400	-1.37590800
H	-4.18625600	-2.87725000	-2.21554900
C	-4.82968700	-2.47866000	-0.19449100
C	-4.80505100	-1.54404200	0.84356900
H	-5.34413900	-1.76171200	1.77351200
C	-4.09047700	-0.35025300	0.74323200
C	-2.84866200	-0.62007700	-2.86008800
H	-3.50748300	0.08386700	-3.39910900
H	-2.72471800	-1.50909300	-3.49594400
H	-1.86982200	-0.12493000	-2.77471200
C	-5.55246000	-3.78031200	-0.03809000
H	-4.89378800	-4.54615500	0.40703000
H	-5.89545000	-4.17487800	-1.00666600
H	-6.42425500	-3.68288100	0.62693600
C	-4.04562100	0.60902700	1.89374000
H	-3.00663300	0.85981300	2.17506600
H	-4.54717200	0.19130800	2.77890800
H	-4.54077800	1.56469100	1.65095000
Br	0.00096500	1.24848600	2.41748500

Table S37: Coordinates for $[Mn^{I}(6dmesbpy^{\bullet -})(CO)_3Br]^-$

Mn	-0.00001700	-0.16996100	0.21781600
O	-0.00029200	-1.72911000	-2.26357700
O	-1.76590100	-2.11899700	1.53977700
O	1.76618800	-2.11882000	1.53963000
N	1.34196000	1.29227400	-0.43254600
N	-1.34212800	1.29229900	-0.43209900
C	-0.00014800	-1.08552100	-1.30083600
C	-1.17509400	-1.30403700	0.97303600
C	1.17525400	-1.30394800	0.97289900
C	2.68669100	1.24004600	-0.54099600
C	3.46968700	2.35598500	-0.81759200
H	4.55445700	2.23856100	-0.89099500
C	2.84361600	3.60792600	-0.99857700
H	3.43438300	4.50554600	-1.20259300
C	1.47728300	3.67272900	-0.93332000
H	0.97174400	4.62714500	-1.09176900
C	0.71056500	2.50394000	-0.66889600
C	-0.71080400	2.50402600	-0.66829900
C	-1.47763500	3.67306400	-0.93130100
H	-0.97216200	4.62761600	-1.08915300
C	-2.84401700	3.60838700	-0.99564800
H	-3.43488100	4.50621500	-1.19846500
C	-3.47003100	2.35632700	-0.81525900
H	-4.55484200	2.23898600	-0.88819100
C	-2.68692200	1.24017900	-0.53985600
C	3.41026700	-0.05565000	-0.41792800
C	3.51789700	-0.89218800	-1.54424100
C	4.23743100	-2.08489700	-1.43312400
H	4.30807300	-2.74483700	-2.30697600
C	4.89180300	-2.44269400	-0.25154800
C	4.82120900	-1.56308600	0.83091300
H	5.33834300	-1.82011700	1.76387500
C	4.08828800	-0.37637500	0.77121900
C	2.94806200	-0.48083300	-2.86906000
H	1.96846700	0.01043000	-2.77409600
H	2.83637000	-1.34238800	-3.54467100
H	3.61527600	0.24653300	-3.36506200
C	5.63206000	-3.74032300	-0.14239700
H	4.97281200	-4.54441200	0.22887300
H	6.47560500	-3.66884600	0.56166000
H	6.02181400	-4.07075200	-1.11767300
C	3.99841700	0.51813000	1.97034300
H	4.47457200	1.49583800	1.78566600
H	4.48909800	0.06392700	2.84429400

H	2.94688400	0.73157900	2.23580300
C	-3.41036100	-0.05565900	-0.41756100
C	-3.51775000	-0.89152400	-1.54441100
C	-4.23705300	-2.08443600	-1.43408200
H	-4.30751700	-2.74384700	-2.30834700
C	-4.89143900	-2.44309500	-0.25276400
C	-4.82110000	-1.56414500	0.83023500
H	-5.33823100	-1.82186200	1.76301000
C	-4.08840100	-0.37724400	0.77132700
C	-2.94789900	-0.47921500	-2.86892700
H	-3.61485200	0.24890400	-3.36417300
H	-2.83665400	-1.34020600	-3.54532800
H	-1.96808500	0.01155200	-2.77363300
C	-5.63143300	-3.74094800	-0.14450100
H	-4.97184200	-4.54531900	0.22554800
H	-6.02169200	-4.07044600	-1.11989300
H	-6.47458600	-3.67032100	0.56010700
C	-3.99877400	0.51651700	1.97103100
H	-2.94731700	0.73056900	2.23629000
H	-4.48882800	0.06135200	2.84483500
H	-4.47573500	1.49399300	1.78720000
Br	0.00008500	1.06995500	2.52757300

Table S39: Coordinates for $Mn^0(6dmesbpy)(CO)_3$

Mn	0.00003200	0.06215600	0.70662300
O	-0.00119900	-2.34070800	-1.04242300
O	-1.83118000	-1.16939200	2.65886500
O	1.83157600	-1.17289300	2.65629200
N	1.30952600	1.49741300	-0.02899900
N	-1.30887000	1.49750100	-0.03011400
C	-0.00098000	-1.37291800	-0.40033400
C	-1.16587600	-0.65178700	1.85698800
C	1.16609000	-0.65390200	1.85546000
C	2.64217500	1.38646200	-0.23538900
C	3.43842000	2.50842300	-0.46151000
H	4.51054900	2.36994300	-0.62400700
C	2.86252000	3.77360600	-0.48786000
H	3.47356000	4.66506900	-0.64594000
C	1.48709800	3.87679500	-0.35006300
H	1.00049800	4.85075600	-0.41465000
C	0.73411600	2.71874900	-0.14988600
C	-0.73317200	2.71865400	-0.15169800
C	-1.48556400	3.87634800	-0.35607300
H	-0.99861500	4.85001200	-0.42241700
C	-2.86072300	3.77312500	-0.49633700
H	-3.47126400	4.66432500	-0.65779100
C	-3.43691600	2.50813500	-0.46793600
H	-4.50882800	2.36947400	-0.63173300
C	-2.64124400	1.38649000	-0.23823700
C	3.25963500	0.03828500	-0.28246700
C	3.03215900	-0.77272700	-1.41284200
C	3.61122000	-2.03991600	-1.45417700
H	3.42764100	-2.67786500	-2.32753100
C	4.42175300	-2.51537000	-0.41755400
C	4.67105500	-1.67012800	0.66388000
H	5.31746300	-2.01840000	1.47871500
C	4.11162400	-0.39153900	0.74736400
C	2.22139200	-0.27218000	-2.57015700
H	1.22699900	0.08162500	-2.25330100
H	2.07812200	-1.05720700	-3.32702900
H	2.71607000	0.58493400	-3.05929000
C	4.99224200	-3.89885100	-0.46780100
H	4.25010200	-4.64604000	-0.13713500
H	5.87047300	-4.00164100	0.18725900
H	5.28800600	-4.17804800	-1.49123400
C	4.42367400	0.48436600	1.92385500
H	5.20476800	1.22567800	1.68061300
H	4.79308000	-0.11212500	2.77138900

H	3.54455200	1.05127500	2.26907300
C	-3.25934700	0.03851200	-0.28305100
C	-3.03331000	-0.77412000	-1.41243900
C	-3.61308500	-2.04111300	-1.45152600
H	-3.43053700	-2.68037200	-2.32413200
C	-4.42298700	-2.51464200	-0.41359500
C	-4.67111100	-1.66762800	0.66679900
H	-5.31723200	-2.01432000	1.48254200
C	-4.11092600	-0.38928300	0.74801400
C	-2.22335500	-0.27546500	-2.57112900
H	-2.71872800	0.58042800	-3.06169800
H	-2.08006700	-1.06188400	-3.32655200
H	-1.22896000	0.07944300	-2.25552200
C	-4.99408300	-3.89798400	-0.46081500
H	-4.25547600	-4.64373700	-0.11912900
H	-5.28126300	-4.18296500	-1.48505700
H	-5.87792200	-3.99634500	0.18738900
C	-4.42183600	0.48884400	1.92314400
H	-3.54192100	1.05500700	2.26758100
H	-4.79221800	-0.10577500	2.77156500
H	-5.20176300	1.23096300	1.67861100

Table S41: Coordinates for $[Mn^0(6dmesbpy^{\bullet-})(CO)_3]^-$

Mn	0.00213000	0.01238000	0.59764700
O	-0.04138100	-2.20451600	-1.31564700
O	-1.78218200	-1.35801900	2.48561200
O	1.80220300	-1.44908800	2.40045800
N	1.28182900	1.47102900	0.07959300
N	-1.28228900	1.47088100	0.07371200
C	-0.03022300	-1.27945900	-0.59937100
C	-1.13614300	-0.77993900	1.70031000
C	1.15501500	-0.83287700	1.64604600
C	2.64594800	1.38892800	-0.10967200
C	3.43571300	2.49746400	-0.32518800
H	4.51267600	2.35258400	-0.45632000
C	2.85971400	3.78508500	-0.41875000
H	3.47889300	4.67140200	-0.57803100
C	1.49201200	3.87046400	-0.36442200
H	0.99539100	4.83491200	-0.49482500
C	0.70957000	2.71264900	-0.15343700
C	-0.71055200	2.71197700	-0.15636100
C	-1.49438700	3.86838600	-0.37182100
H	-0.99941300	4.83358300	-0.50249400
C	-2.86168600	3.77996300	-0.42996200
H	-3.48171100	4.66511300	-0.59269000
C	-3.43684100	2.49248300	-0.33376600
H	-4.51361800	2.34605100	-0.46445800
C	-2.64504500	1.38567900	-0.11476900
C	3.30023000	0.05544600	-0.18460900
C	3.15050700	-0.70627700	-1.36303600
C	3.78244200	-1.94557200	-1.45218100
H	3.65410300	-2.54260200	-2.36430700
C	4.58919500	-2.43924500	-0.41967900
C	4.76767200	-1.64165800	0.70952900
H	5.40638600	-2.00323500	1.52537500
C	4.13868800	-0.39853600	0.84470300
C	2.36659200	-0.17086700	-2.52265000
H	1.37290600	0.18559700	-2.20415600
H	2.22822200	-0.93466600	-3.30259600
H	2.88139300	0.69295900	-2.97886300
C	5.22804400	-3.78998800	-0.52922300
H	4.50063500	-4.59508600	-0.32537100
H	6.05475700	-3.90932200	0.18757300
H	5.62192100	-3.97162600	-1.54217900
C	4.36092000	0.41569300	2.08443100
H	5.12587500	1.19646500	1.92882800
H	4.70433000	-0.21800200	2.91664600

H	3.44322600	0.93293200	2.40622400
C	-3.29668700	0.05052300	-0.18463200
C	-3.15828300	-0.70709000	-1.36680400
C	-3.78809500	-1.94772800	-1.45292700
H	-3.66830300	-2.54155600	-2.36827400
C	-4.57905300	-2.44799200	-0.41169500
C	-4.74825600	-1.65348700	0.72134400
H	-5.37800900	-2.01861800	1.54259400
C	-4.12472100	-0.40707000	0.85166000
C	-2.38999900	-0.16527600	-2.53399700
H	-2.91768100	0.69254300	-2.98683100
H	-2.25110800	-0.92876400	-3.31414800
H	-1.39685500	0.20161300	-2.22618000
C	-5.21218700	-3.80188600	-0.51567500
H	-4.48261000	-4.60276800	-0.30295500
H	-5.60063500	-3.99151700	-1.52918500
H	-6.04150500	-3.92013800	0.19834100
C	-4.35032800	0.41075900	2.08837100
H	-3.44003300	0.94622700	2.40032200
H	-4.67624000	-0.22344800	2.92716500
H	-5.13077300	1.17615100	1.93250200

Table S43: Coordinates for $Mn^0(6dmesbpy)(CO)_3(DMSO)$

Mn	-0.00833800	-0.10448000	-0.47983400
O	0.00784900	-1.34982100	-3.13463300
O	1.67810400	2.15384300	-1.34837100
O	-1.76059500	2.06392500	-1.43433100
N	-1.33679000	-1.55927500	0.18214400
N	1.35781700	-1.55866100	0.12932100
C	0.00065000	-0.83141400	-2.10284200
C	1.14681900	1.19985400	-0.97229000
C	-1.20015900	1.15046300	-1.00228600
C	-2.68819500	-1.52386200	0.24309700
C	-3.45341800	-2.60648600	0.65830300
H	-4.54161700	-2.50343800	0.69066200
C	-2.81130400	-3.80518400	1.03337700
H	-3.39047000	-4.67226400	1.36247800
C	-1.44406600	-3.85704900	0.98412100
H	-0.92663100	-4.77117600	1.27950700
C	-0.69270400	-2.72730000	0.56046400
C	0.73041800	-2.73527600	0.50743100
C	1.49872000	-3.88962600	0.82141300
H	0.99439000	-4.81628700	1.10015800
C	2.86626900	-3.85171800	0.76780200
H	3.45798200	-4.74006300	1.00502700
C	3.49320300	-2.63912200	0.40991000
H	4.58204400	-2.54212200	0.37980100
C	2.71079300	-1.53399100	0.10263500
C	-3.44758800	-0.29302500	-0.11187800
C	-3.98144100	-0.14331300	-1.40425200
C	-4.76157700	0.97994500	-1.68518000
H	-5.16690000	1.10428200	-2.69697500
C	-5.03311700	1.95036600	-0.71823300
C	-4.53040700	1.75435800	0.57078600
H	-4.75959700	2.48859500	1.35373200
C	-3.75593100	0.63922500	0.89694600
C	-3.71081600	-1.16254000	-2.46936600
H	-2.63051600	-1.28744400	-2.65287300
H	-4.18250200	-0.88150100	-3.42225600
H	-4.08906700	-2.15774400	-2.18201700
C	-5.82504400	3.17504700	-1.05728000
H	-5.16368100	3.99152600	-1.39629700
H	-6.38133300	3.55477900	-0.18637000
H	-6.54141800	2.98439800	-1.87117600
C	-3.28335100	0.42235200	2.30212000
H	-3.74654400	-0.47707200	2.74305200
H	-3.53700400	1.27720200	2.94951800

H	-2.19246100	0.26061600	2.32975900
C	3.44578500	-0.27399500	-0.19395500
C	3.95326500	-0.02487600	-1.48147200
C	4.70107400	1.13381700	-1.69630600
H	5.08596600	1.33708400	-2.70331900
C	4.96363000	2.04486700	-0.67003100
C	4.48855300	1.75046200	0.61022100
H	4.71101100	2.43643600	1.43720000
C	3.74970600	0.59475000	0.87040300
C	3.69256200	-0.97859500	-2.60783700
H	4.12790300	-1.97142100	-2.40549300
H	4.11713900	-0.60841400	-3.55242000
H	2.61388100	-1.14424200	-2.76545800
C	5.71664800	3.31053800	-0.94041100
H	5.03089400	4.12024400	-1.24552600
H	6.44499500	3.18444300	-1.75635100
H	6.25348700	3.66452100	-0.04687900
C	3.33374000	0.25726300	2.27028100
H	2.29563100	-0.11228000	2.30587300
H	3.43193700	1.12595100	2.93984400
H	3.96096100	-0.55240200	2.68284500
O	-0.06346700	0.49964600	1.54707900
S	0.48965500	1.87051500	2.01975900
C	0.24771700	1.77461300	3.79567400
H	0.90836300	0.98058500	4.16833400
H	-0.80488400	1.52723500	3.99659700
H	0.52480800	2.73787900	4.24654600
C	-0.79519500	3.08331800	1.65039200
H	-0.75981900	3.30300500	0.57506200
H	-0.57228400	3.99837000	2.21820900
H	-1.77443400	2.66609600	1.93017100

Table S45: Coordinates for $[Mn^{I}(6dmesbpy)(CO)_3(DMSO)]^+$

Mn	-0.07031000	0.04830300	-0.33437400
O	-0.24405400	2.69193000	0.95846000
O	-1.90619100	0.91504300	-2.47955500
O	1.73685800	1.30110400	-2.30581200
N	1.31256400	-0.89359500	0.94235900
N	-1.34063000	-0.99716900	0.97030200
C	-0.17261600	1.63991000	0.49717600
C	-1.28058100	0.57849900	-1.57703800
C	1.11001700	0.78152300	-1.49319100
C	2.64147400	-0.68954400	1.04364700
C	3.47734000	-1.63894200	1.63977900
H	4.54831800	-1.43184000	1.70620500
C	2.93580300	-2.80948900	2.14854100
H	3.57310300	-3.57099300	2.60321500
C	1.55821100	-2.97720900	2.10787900
H	1.10404500	-3.86760800	2.54259400
C	0.77092100	-1.98956300	1.51945100
C	-0.70115100	-2.02991100	1.56331400
C	-1.38905400	-3.02898700	2.24820800
H	-0.85347800	-3.85550000	2.71516500
C	-2.77096200	-2.95215100	2.35005100
H	-3.33098500	-3.72338900	2.88339300
C	-3.41741600	-1.86522800	1.78370600
H	-4.50041200	-1.74006600	1.86277600
C	-2.67698600	-0.89884600	1.09660200
C	3.22388400	0.59993200	0.60110700
C	2.98588900	1.74324000	1.39089100
C	3.52565000	2.96198100	0.97858500
H	3.33179900	3.85718200	1.58169600
C	4.32708200	3.06591600	-0.16210400
C	4.60338100	1.90220600	-0.88279900
H	5.24547600	1.96164800	-1.76955700
C	4.06688100	0.66495100	-0.52252700
C	2.24459800	1.65022900	2.69214800
H	1.29492000	1.09830500	2.61323700
H	2.02286300	2.64840800	3.09654700
H	2.85044300	1.11133000	3.44175200
C	4.86467200	4.39091900	-0.60381400
H	4.14525000	4.90739400	-1.26258700
H	5.80022200	4.28057300	-1.17269900
H	5.05221900	5.05875500	0.25080700
C	4.34789400	-0.54526500	-1.36158500
H	4.81965200	-1.35830400	-0.78637500
H	5.01523700	-0.29907500	-2.19982900

H	3.42228500	-0.96427600	-1.79403300
C	-3.39508200	0.26928700	0.53265400
C	-3.39957100	1.48324700	1.24494800
C	-4.08488800	2.56995700	0.70002500
H	-4.07948700	3.52419400	1.24057200
C	-4.79464100	2.46765400	-0.50063000
C	-4.82283500	1.23147200	-1.14979700
H	-5.38263300	1.13032200	-2.08735300
C	-4.13308500	0.12408200	-0.65381700
C	-2.75143200	1.59266800	2.59293100
H	-3.36219200	1.08017100	3.35700500
H	-2.64511700	2.64280700	2.90188700
H	-1.75465900	1.12574700	2.63205900
C	-5.49161300	3.65789100	-1.08249500
H	-4.81225300	4.23216800	-1.73594400
H	-5.84094100	4.34752500	-0.29893600
H	-6.35538900	3.36247200	-1.69720300
C	-4.15178300	-1.17943600	-1.39525600
H	-3.14446100	-1.62306300	-1.47713100
H	-4.54869000	-1.05052200	-2.41303300
H	-4.78185600	-1.92944400	-0.88693100
O	-0.11773700	-1.82723000	-1.21377300
S	0.87589200	-2.27204400	-2.32203500
C	-0.21349400	-2.94362800	-3.57710300
H	-0.79271200	-2.09639400	-3.96967200
H	-0.87601600	-3.68238700	-3.10321600
H	0.38976900	-3.39412000	-4.37775600
C	1.55648700	-3.79539800	-1.66311700
H	2.22575100	-3.51943500	-0.83495600
H	2.12797200	-4.30565500	-2.45174700
H	0.72291500	-4.41503500	-1.29942600

Table S47: Coordinates for Mn^{I}(6dmesbpy)$(CO)_3$H

Mn	0.00028200	0.04148700	0.77464300
O	0.00067700	-2.10505300	-1.28013100
O	-1.85335300	-1.41812200	2.51464900
O	1.85378200	-1.41599900	2.51650600
N	1.31429700	1.48910000	-0.00288900
N	-1.31524000	1.48870200	-0.00130100
C	0.00065100	-1.19672200	-0.56174800
C	-1.18585700	-0.84186300	1.76370600
C	1.18645100	-0.84066000	1.76471700
C	2.64338300	1.38634100	-0.20143900
C	3.43906300	2.51380400	-0.42309700
H	4.51296000	2.37878300	-0.57705900
C	2.85733600	3.77234300	-0.45856800
H	3.46270400	4.66783400	-0.61587700
C	1.47906500	3.86546500	-0.33003800
H	0.98732500	4.83600500	-0.40263700
C	0.73415500	2.70441100	-0.12547600
C	-0.73589200	2.70452800	-0.12185500
C	-1.48211400	3.86629000	-0.31735800
H	-0.99125500	4.83761100	-0.38540000
C	-2.86080900	3.77296500	-0.44144800
H	-3.46729200	4.66891600	-0.59168400
C	-3.44169700	2.51388800	-0.41091600
H	-4.51592700	2.37909500	-0.56266600
C	-2.64479700	1.38587700	-0.19649800
C	3.27711600	0.04609000	-0.26150600
C	3.09726200	-0.73158300	-1.42257200
C	3.69515600	-1.98983800	-1.48205200
H	3.54714400	-2.60504100	-2.37817700
C	4.48673900	-2.48062500	-0.43796400
C	4.69366400	-1.66273400	0.67350400
H	5.32336200	-2.02403200	1.49567400
C	4.10678000	-0.39878700	0.77916200
C	2.32716200	-0.19995300	-2.59423600
H	1.33779900	0.18875600	-2.30404000
H	2.17590300	-0.97604100	-3.35873200
H	2.86430300	0.64065500	-3.06752700
C	5.08215300	-3.85259300	-0.51104200
H	4.34987400	-4.61936400	-0.20409600
H	5.95460200	-3.95379300	0.15197300
H	5.39422400	-4.10485800	-1.53653600
C	4.35634200	0.44837300	1.99089700
H	5.10855000	1.23115800	1.79058500
H	4.73173900	-0.15970200	2.82742100

H	3.44503300	0.96619700	2.33175700
C	-3.27733200	0.04529500	-0.26093500
C	-3.09516200	-0.72922200	-1.42374100
C	-3.69206400	-1.98779900	-1.48744000
H	-3.54236700	-2.60039700	-2.38506100
C	-4.48458200	-2.48206300	-0.44579300
C	-4.69360600	-1.66731500	0.66767000
H	-5.32414500	-2.03138700	1.48797500
C	-4.10799700	-0.40319800	0.77751200
C	-2.32382800	-0.19411800	-2.59303200
H	-2.86048800	0.64786700	-3.06441000
H	-2.17169800	-0.96798500	-3.35960200
H	-1.33482500	0.19379500	-2.30055900
C	-5.07863100	-3.85440900	-0.52267300
H	-4.35085700	-4.61985300	-0.20194000
H	-5.37577900	-4.11051000	-1.55158800
H	-5.96048600	-3.95343500	0.12817400
C	-4.36000500	0.44014100	1.99143800
H	-3.45009100	0.95917900	2.33411300
H	-4.73426600	-0.17119100	2.82610100
H	-5.11413600	1.22157100	1.79311700
H	0.00015100	0.99776200	2.07268900

Table S49: Coordinates for Mer-Mn^{I}(6dmesbpy)$(CO)_3$Br

Mn	-0.18804600	0.13418200	0.65680900
O	0.00062300	-2.09986400	-1.32621700
O	-2.03105300	-1.67057200	2.06816900
O	-0.06762200	1.73729200	3.20307400
N	1.20092000	1.53858100	-0.23587800
N	-1.45083000	1.48855400	-0.25889700
C	-0.07657700	-1.17235600	-0.65308900
C	-1.37891500	-0.92462000	1.47470700
C	-0.12842000	1.17245400	2.20850900
C	2.53113300	1.46874700	-0.43759700
C	3.30038000	2.62552800	-0.61140200
H	4.37609200	2.52323000	-0.77514100
C	2.69184500	3.87016700	-0.59833600
H	3.27932400	4.78448700	-0.70794700
C	1.30864100	3.92733600	-0.50048400
H	0.79548600	4.88801400	-0.54951100
C	0.59121500	2.74113500	-0.35539400
C	-0.87990300	2.70464000	-0.43191100
C	-1.62769400	3.83384000	-0.76235200
H	-1.14457200	4.80360100	-0.88259400
C	-2.99427100	3.70878800	-0.96540200
H	-3.59978700	4.57988900	-1.22492000
C	-3.56072500	2.44717400	-0.86909400
H	-4.62264500	2.28287600	-1.07013600
C	-2.76745800	1.35128900	-0.51831200
C	3.19924800	0.15640800	-0.61012800
C	2.91549700	-0.58831800	-1.77057600
C	3.56226000	-1.81250500	-1.95356000
H	3.33312000	-2.40276100	-2.84937900
C	4.50726100	-2.29106900	-1.04274400
C	4.81572100	-1.49759100	0.06462000
H	5.56546700	-1.85014700	0.78363600
C	4.17765600	-0.27916100	0.29995000
C	2.00461400	-0.05800500	-2.83981000
H	1.04290300	0.30610400	-2.44591500
H	1.79180700	-0.82552700	-3.59857100
H	2.46863400	0.80243300	-3.35381900
C	5.15953400	-3.62443800	-1.23928200
H	4.58696300	-4.42291100	-0.73597800
H	6.17612400	-3.64562800	-0.81729100
H	5.21955900	-3.89560800	-2.30448200
C	4.52582700	0.52562900	1.51562000
H	5.20650200	1.36136700	1.27713100
H	5.03099000	-0.09909000	2.26774500

H	3.62788500	0.95741500	1.98621600
C	-3.39756900	0.00829600	-0.49879500
C	-3.19669200	-0.85132400	-1.59574400
C	-3.80602900	-2.10652400	-1.57981000
H	-3.63979600	-2.78653100	-2.42424700
C	-4.63833800	-2.50892600	-0.53064700
C	-4.86592500	-1.61028200	0.51338900
H	-5.52229900	-1.90508500	1.34103400
C	-4.25782400	-0.35436900	0.55022200
C	-2.40145800	-0.41026300	-2.78820100
H	-2.96126500	0.34032600	-3.37343000
H	-2.17708600	-1.25521300	-3.45572800
H	-1.44776600	0.06629800	-2.51108700
C	-5.25256700	-3.87419600	-0.51934400
H	-4.57765900	-4.60698700	-0.04398600
H	-5.45287800	-4.23837900	-1.53869400
H	-6.19565600	-3.89072700	0.04783300
C	-4.49319500	0.56732400	1.70889400
H	-3.55196400	0.99995400	2.08871900
H	-4.98235300	0.04078800	2.54155000
H	-5.13789700	1.41921800	1.43258000
Br	1.49554500	-1.29941700	1.95417200

5.2.4 Coordinates for Mn4dicarb species

Table S51: Coordinates for $Mn^{I}(4dcarbpy)(CO)_3Br$

Mn	0.00003400	-2.12766000	0.42777800
N	-1.30682100	-0.53765500	0.35881400
C	-0.00003400	-2.15949800	2.22447600
C	-1.28090900	-3.39515700	0.30259300
C	1.28104000	-3.39510000	0.30267800
N	1.30682400	-0.53760200	0.35884500
C	-2.64012100	-0.62852300	0.36671700
C	-3.47066000	0.47819200	0.25162100
C	-2.88665500	1.73672200	0.12362900
C	-1.49937900	1.83440300	0.11528500
C	-0.73399600	0.67763800	0.23339000
C	0.73395500	0.67766700	0.23339500
C	1.49929300	1.83445800	0.11526400
C	2.88657200	1.73682900	0.12361000
C	3.47062500	0.47832400	0.25163200
C	2.64012700	-0.62842000	0.36674900
Br	0.00007000	-2.01779600	-2.10675500
C	-3.68260600	2.99431200	-0.00544500
C	3.68247700	2.99444500	-0.00550200
O	0.00006800	-2.18067800	3.37549200
O	2.10160100	-4.19369300	0.19308400
O	-2.10139300	-4.19380500	0.19284000
O	4.98697300	2.76185500	0.01752700
O	-4.98709300	2.76166700	0.01753700
O	3.18412300	4.08696400	-0.11691900
O	-3.18429300	4.08684900	-0.11686800
H	-3.07199000	-1.62752000	0.46819400
H	-4.55446200	0.35626300	0.26234100
H	-1.03956700	2.81950400	0.01445500
H	1.03944500	2.81954000	0.01440500
H	4.55443200	0.35643700	0.26235300
H	3.07203500	-1.62739800	0.46824500
C	5.83995600	3.89458100	-0.09988700
H	5.66391900	4.59804100	0.72712300
H	6.86737400	3.51740300	-0.06207100
H	5.66217600	4.41473400	-1.05257900
C	-5.84011800	3.89436000	-0.09989200
H	-6.86752200	3.51713900	-0.06211500
H	-5.66413700	4.59781700	0.72713300
H	-5.66232800	4.41453200	-1.05257100

Table S52: Coordinates for $[Mn^{I}(4dcarbpy^{\bullet -})(CO)_3Br]^-$

Mn	0.00001500	-2.12615900	0.45404400
N	-1.30923100	-0.55538400	0.35822000
C	-0.00002100	-2.11020900	2.23603800
C	-1.28298500	-3.38498000	0.35007500
C	1.28305500	-3.38494700	0.35016000
N	1.30923100	-0.55535600	0.35826200
C	-2.65260700	-0.62246200	0.36534200
C	-3.48045200	0.47259200	0.24887500
C	-2.88187700	1.75590700	0.11704800
C	-1.50060400	1.83663700	0.11183200
C	-0.71738500	0.67934500	0.23010400
C	0.71736300	0.67936000	0.23012200
C	1.50056000	1.83666700	0.11186100
C	2.88183500	1.75596600	0.11711500
C	3.48043300	0.47266400	0.24896700
C	2.65260900	-0.62240500	0.36542100
Br	0.00007500	-2.09801400	-2.14418100
C	-3.65609300	3.00340100	-0.01534400
C	3.65602900	3.00347400	-0.01526400
O	-0.00008200	-2.08507400	3.39068300
O	2.10912200	-4.18566000	0.25983900
O	-2.10906100	-4.18569400	0.25981400
O	4.97687700	2.78374500	0.00122700
O	-4.97693700	2.78365100	0.00119200
O	3.17417200	4.11076200	-0.12768400
O	-3.17425700	4.11070500	-0.12767800
H	-3.08973000	-1.62136400	0.46841200
H	-4.56418000	0.35074700	0.25818900
H	-1.03391900	2.81972100	0.01048200
H	1.03385900	2.81974000	0.01049200
H	4.56416400	0.35084200	0.25831200
H	3.08975000	-1.62129800	0.46851300
C	5.80849600	3.92408400	-0.12115300
H	5.63269400	4.63114400	0.70411200
H	6.84381100	3.56547000	-0.08934800
H	5.62349400	4.44719800	-1.07195000
C	-5.80857700	3.92398300	-0.12111400
H	-5.62362200	4.44713100	-1.07190100
H	-6.84388500	3.56535300	-0.08927800
H	-5.63275100	4.63101700	0.70416800

Table S53: Coordinates for $Mn^0(4dcarbpy)(CO)_3$

Mn	0.00032400	2.41563700	-0.12125800
N	1.28663100	0.87311400	-0.12125800
C	0.00357600	2.91130700	1.58840100
C	1.25919400	3.60628200	-0.59877600
C	-1.25897700	3.60767900	-0.59413100
N	-1.28667200	0.87376700	-0.12060500
C	2.63607900	0.95676900	-0.09162800
C	3.46398300	-0.14421800	-0.05982600
C	2.88683800	-1.42674900	-0.05225200
C	1.50369700	-1.52676600	-0.07020300
C	0.72436100	-0.36957700	-0.10026400
C	-0.72495400	-0.36923100	-0.10007100
C	-1.50476300	-1.52608600	-0.07024500
C	-2.88787100	-1.42552800	-0.05205900
C	-3.46446300	-0.14273300	-0.05913000
C	-2.63612100	0.95791200	-0.09073600
C	3.68545100	-2.67855700	-0.02070000
C	-3.68699000	-2.67699800	-0.02086800
O	0.00565900	3.20684100	2.70582800
O	-2.07117900	4.36935900	-0.90441200
O	2.07112100	4.36695000	-0.91224600
O	-4.99521500	-2.43582000	-0.00251800
O	4.99376800	-2.43792000	-0.00256200
O	-3.20530800	-3.78529200	-0.01246900
O	3.20329500	-3.78663500	-0.01184100
H	3.06560600	1.96171600	-0.09894200
H	4.54687400	-0.01443400	-0.04178800
H	1.04424100	-2.51769900	-0.05768600
H	-1.04569700	-2.51720500	-0.05818500
H	-4.54729700	-0.01249000	-0.04090000
H	-3.06528200	1.96301100	-0.09768700
C	-5.85044600	-3.56959100	0.03013700
H	-5.66182700	-4.17345500	0.93030900
H	-6.87726200	-3.18808000	0.04248400
H	-5.69364900	-4.20121500	-0.85700000
C	5.84852900	-3.57204000	0.03045600
H	6.87550500	-3.19095300	0.04259300
H	5.65971100	-4.17548700	0.93086600
H	5.69140200	-4.20392500	-0.85643600

Table S54: Coordinates for [Mn^0(4dcarbpy$^{\bullet -}$)$(CO)_3$]$^-$

Mn	0.00171000	2.40111000	-0.07731300
N	1.26364500	0.89375200	-0.12762500
C	0.03111700	2.94353100	1.59809700
C	1.24574000	3.55083500	-0.63831700
C	-1.24911600	3.56395600	-0.59468900
N	-1.26418200	0.89922300	-0.12762600
C	2.63187900	0.95998700	-0.09767500
C	3.45671100	-0.12765600	-0.06464000
C	2.88463500	-1.43664500	-0.04739800
C	1.50615700	-1.52848100	-0.05491300
C	0.70826000	-0.37586900	-0.08861300
C	-0.71316300	-0.37324400	-0.08859400
C	-1.51407600	-1.52341000	-0.05508000
C	-2.89249300	-1.42844800	-0.04798300
C	-3.46045100	-0.11735300	-0.06589400
C	-2.63305900	0.96800200	-0.09887200
C	3.67676200	-2.67160400	-0.01568500
C	-3.68770700	-2.66078200	-0.01608500
O	0.05103900	3.28337500	2.71058500
O	-2.06597200	4.31780000	-0.93791700
O	2.05881900	4.29501200	-1.01044400
O	-5.00830100	-2.41540800	-0.01123200
O	4.99771200	-2.42983400	-0.01099500
O	-3.23408400	-3.78727400	0.00448900
O	3.21967700	-3.79653000	0.00420300
H	3.06276000	1.96553100	-0.11054700
H	4.53934300	0.00981600	-0.05124000
H	1.04261100	-2.51870900	-0.03201300
H	-1.05288400	-2.51475500	-0.03194400
H	-4.54271200	0.02336500	-0.05337300
H	-3.06207300	1.97423900	-0.11283100
C	-5.85913900	-3.54573300	0.01881800
H	-5.68446400	-4.15010400	0.92253700
H	-6.88846600	-3.16812100	0.01893700
H	-5.69829700	-4.18704300	-0.86173100
C	5.84561500	-3.56253300	0.01830400
H	6.87590400	-3.18761300	0.01836000
H	5.66940400	-4.16677100	0.92178600
H	5.68272100	-4.20285900	-0.86255700

Table S55: Coordinates for (ps)-[Mn^0(4dcarbpy)$(CO)_3$]$_2$

Mn	-0.59204100	-1.01937500	-1.38082700
N	-1.41536100	0.79739700	-1.09075400
C	-1.00947400	-1.09856500	-3.10552800
C	1.03946900	-0.37347500	-1.72402700
C	0.10305900	-2.67398400	-1.39868800
N	-2.43088700	-1.54359300	-0.73498800
C	-0.80920400	1.98504600	-1.29503000
C	-1.43831100	3.19962300	-1.10277100
C	-2.77464100	3.20910800	-0.68035200
C	-3.41535500	1.99190700	-0.49112500
C	-2.72021500	0.80344300	-0.70674800
C	-3.29545100	-0.51811100	-0.51890300
C	-4.61565800	-0.74103000	-0.12741500
C	-5.06787100	-2.03894200	0.05379400
C	-4.17333600	-3.09581200	-0.17243200
C	-2.88375800	-2.80345700	-0.56729400
C	-3.53601700	4.45945500	-0.42367100
C	-6.47840400	-2.24403300	0.47787700
O	-1.25757100	-1.15193700	-4.23253000
O	0.57158500	-3.73120400	-1.46214200
O	2.07963800	0.05132500	-2.01152300
O	-6.77720100	-3.53206400	0.61710400
O	-2.79448700	5.54564200	-0.62087400
O	-7.26028900	-1.34427400	0.67292700
O	-4.69187000	4.48485500	-0.07364100
H	0.23336000	1.95791300	-1.62507800
H	-0.89255200	4.12927000	-1.27350600
H	-4.45881800	1.98611400	-0.16872000
H	-5.30277000	0.09011500	0.04515200
H	-4.48383000	-4.13354300	-0.04344800
H	-2.17051300	-3.61074700	-0.75229900
Mn	0.59202900	-1.01942800	1.38076900
N	1.41539800	0.79729200	1.09038800
C	1.00965400	-1.09794900	3.10546400
C	-1.03938100	-0.37333900	1.72364900
C	-0.10375300	-2.67380700	1.39934500
N	2.43068900	-1.54378300	0.73456700
C	0.80924800	1.98497400	1.29446800
C	1.43848600	3.19951000	1.10234900
C	2.77495300	3.20889900	0.68038400
C	3.41566700	1.99165500	0.49141400
C	2.72036900	0.80324000	0.70680600
C	3.29544500	-0.51837400	0.51892000
C	4.61566900	-0.74138100	0.12752800

C	5.06774700	-2.03931900	-0.05388600
C	4.17294500	-3.09610100	0.17168000
C	2.88331700	-2.80364700	0.56629900
C	3.53650200	4.45919400	0.42394000
C	6.47836700	-2.24451300	-0.47761900
O	1.25777700	-1.15060400	4.23249500
O	-0.57332000	-3.73052500	1.46324800
O	-2.07968900	0.05156700	2.01049900
O	6.77707200	-3.53256300	-0.61688000
O	2.79499200	5.54543200	0.62091700
O	7.26039100	-1.34481400	-0.67238900
O	4.69247800	4.48450700	0.07431500
H	-0.23339700	1.95792000	1.62425400
H	0.89273200	4.12919900	1.27287400
H	4.45925500	1.98580500	0.16942000
H	5.30292800	0.08970400	-0.04473300
H	4.48324100	-4.13384200	0.04229300
H	2.16962900	-3.61076500	0.75027900
C	-3.42732600	6.79814400	-0.39875700
H	-3.77459900	6.87916900	0.64206500
H	-2.67739100	7.56946900	-0.60525500
H	-4.29048000	6.92241100	-1.06953700
C	-8.10460100	-3.83808700	1.02123500
H	-8.16993400	-4.92981800	1.08282500
H	-8.32527500	-3.39128000	2.00208800
H	-8.83172000	-3.45755100	0.28852600
C	8.10454600	-3.83868600	-1.02069200
H	8.16979900	-4.93041900	-1.08230900
H	8.32550100	-3.39186000	-2.00147400
H	8.83151800	-3.45823900	-0.28779100
C	3.42799800	6.79789100	0.39902400
H	3.77558300	6.87891700	-0.64169400
H	2.67806300	7.56927000	0.60532300
H	4.29096400	6.92206800	1.07006300

Table S57: Coordinates for (pe)-[Mn^0(4dcarbpy)$(CO)_3$]$_2$

Mn	-0.09488100	2.40041800	-1.57910400
N	-1.09456700	0.66473400	-1.62865400
C	-0.02506000	2.58599600	-3.34120300
C	-1.60014400	3.37269300	-1.45356400
C	0.89498900	3.88554800	-1.33250800
N	1.43739100	1.09374700	-1.45924600
C	-2.43339700	0.51706600	-1.77627300
C	-3.06002900	-0.70733200	-1.82964700
C	-2.28309400	-1.87348800	-1.71650300
C	-0.90647900	-1.73877600	-1.63314200
C	-0.33415900	-0.46550900	-1.59450800
C	1.09297600	-0.22314600	-1.51733800
C	2.05435800	-1.23607900	-1.49985800
C	3.39721300	-0.90766700	-1.41614900
C	3.74927500	0.45079400	-1.37052700
C	2.74839300	1.40194500	-1.40064200
C	-2.86314700	-3.23845500	-1.63817600
C	4.39039700	-2.00641000	-1.30245900
O	0.02229600	2.70154800	-4.48901800
O	1.50941900	4.86170700	-1.25116000
O	-2.58950400	3.96845400	-1.40621900
O	5.62913800	-1.54485900	-1.17259400
O	-4.18726200	-3.23155900	-1.77183600
O	4.10064500	-3.18106100	-1.30275500
O	-2.20692900	-4.23695900	-1.45842700
H	-3.02722800	1.43229000	-1.83784000
H	-4.14753800	-0.76027500	-1.91375600
H	-0.28890500	-2.63794800	-1.56702000
H	1.76620300	-2.29034900	-1.52520700
H	4.79344900	0.75987100	-1.30526800
H	3.00160500	2.46548200	-1.36190500
Mn	0.09484500	2.39823200	1.58032200
N	1.09465900	0.66255600	1.62826500
C	0.02533400	2.58184400	3.34263700
C	1.60010600	3.37065800	1.45563400
C	-0.89531900	3.88349700	1.33576000
N	-1.43728700	1.09170100	1.45905500
C	2.43344100	0.51483500	1.77609600
C	3.06011900	-0.70958500	1.82867900
C	2.28325400	-1.87565800	1.71430000
C	0.90666400	-1.74095000	1.63050200
C	0.33427500	-0.46767400	1.59290400
C	-1.09287200	-0.22526800	1.51600800
C	-2.05428300	-1.23816300	1.49805400

C	-3.39717600	-0.90967800	1.41509500
C	-3.74921000	0.44882300	1.37043300
C	-2.74830200	1.39993500	1.40087100
C	2.86337600	-3.24058700	1.63557900
C	-4.39052000	-2.00829400	1.30144500
O	-0.02167400	2.69598700	4.49060500
O	-1.51028200	4.85944200	1.25581800
O	2.58970400	3.96608600	1.40909000
O	-5.62938900	-1.54654500	1.17353600
O	4.18737600	-3.23381400	1.77046700
O	-4.10082200	-3.18294800	1.30056900
O	2.20731700	-4.23895400	1.45457000
H	3.02721800	1.43003000	1.83864000
H	4.14759000	-0.76254400	1.91322900
H	0.28915200	-2.64010000	1.56347800
H	-1.76612700	-2.29245600	1.52267200
H	-4.79339100	0.75797700	1.30569700
H	-3.00153900	2.46349200	1.36287500
C	-4.83711400	-4.49069600	-1.65642000
H	-4.73697200	-4.87067900	-0.62789800
H	-5.89340000	-4.32218600	-1.89721200
H	-4.40503700	-5.22036500	-2.35647600
C	6.66494300	-2.50896500	-1.04225500
H	7.60767000	-1.95277300	-1.00311900
H	6.53559400	-3.09154100	-0.11839600
H	6.66640000	-3.19813900	-1.89933200
C	-6.66569500	-2.51018400	1.04409800
H	-7.60867700	-1.95391200	1.01266900
H	-6.54164700	-3.08817000	0.11658300
H	-6.66256100	-3.20352700	1.89778200
C	4.83718900	-4.49299000	1.65534500
H	4.73705200	-4.87330300	0.62693800
H	5.89349500	-4.32448000	1.89615000
H	4.40504800	-5.22248400	2.35555100

Table S59: Coordinates for $Mn^0(4dcarbpy)(CO)_3(DMSO)$

Mn	0.04969000	-2.22324400	-0.59276000
N	1.33003500	-0.63425900	-0.45704300
C	0.07850000	-2.15900200	-2.37862200
C	1.35334200	-3.47407500	-0.52751100
C	-1.22019200	-3.51038000	-0.57503100
N	-1.27955000	-0.67346000	-0.49057000
C	2.67436500	-0.68996000	-0.37875400
C	3.48403900	0.41972400	-0.28939000
C	2.87177400	1.70137200	-0.29561200
C	1.49303600	1.77175600	-0.37693800
C	0.72532600	0.60087300	-0.44765300
C	-0.70959700	0.58034600	-0.45242000
C	-1.51176400	1.72714000	-0.37081600
C	-2.89030500	1.61940300	-0.32474800
C	-3.46709900	0.31930600	-0.37196700
C	-2.62536000	-0.76546800	-0.46056800
C	3.63366900	2.96440300	-0.21409600
C	-3.68654000	2.85723400	-0.22392600
O	0.09826800	-2.07392700	-3.52776600
O	-2.03456600	-4.32448300	-0.55346300
O	2.18826800	-4.26549400	-0.47156400
O	-5.00027000	2.60980400	-0.17916900
O	4.95093000	2.75425000	-0.12974200
O	-3.22207000	3.97619000	-0.18405400
O	3.13798200	4.06991000	-0.22078200
H	3.12294900	-1.68847400	-0.38574800
H	4.56682700	0.30990500	-0.22116400
H	1.01599200	2.75491500	-0.37494700
H	-1.06179900	2.72224600	-0.33195100
H	-4.54810600	0.17870600	-0.34213800
H	-3.04600900	-1.77542500	-0.50147200
O	-0.00490800	-2.25737100	1.51501400
S	0.11363700	-0.95671300	2.34715300
C	1.02110200	-1.50359800	3.79525300
H	2.03850200	-1.74076600	3.45619800
H	0.52771200	-2.39951300	4.19957600
H	1.04938700	-0.69041800	4.53455700
C	-1.51379500	-0.77184300	3.09180500
H	-2.21042300	-0.51472500	2.28005600
H	-1.48338700	0.04177900	3.83080600
H	-1.79735000	-1.72769200	3.55624900
C	5.77182000	3.90720700	-0.04555300
H	6.80732600	3.55362900	0.01586400
H	5.64601400	4.54505900	-0.93367700

H	5.52522500	4.50089300	0.84787500
C	-5.85343600	3.73734500	-0.07981100
H	-5.72445000	4.40605900	-0.94454700
H	-6.88032100	3.35519400	-0.05435200
H	-5.64431500	4.30944400	0.83703300

Table S61: Coordinates for $[Mn^{I}(4dcarbpy)(CO)_3(DMSO)]^+$

Mn	-0.11960500	-2.18743600	-0.60572300
N	1.32084900	-0.71325800	-0.56852000
C	-0.16930600	-2.24169200	-2.40646300
C	1.05414300	-3.57258200	-0.53557300
C	-1.50624200	-3.34646500	-0.44199900
N	-1.28840400	-0.49163800	-0.57770700
C	2.63912800	-0.92226500	-0.52385200
C	3.55771500	0.10832300	-0.36643500
C	3.08065400	1.41137700	-0.25217400
C	1.70773800	1.63200100	-0.30137900
C	0.85078200	0.54757300	-0.46073500
C	-0.61290500	0.67457300	-0.49394000
C	-1.27625000	1.89386000	-0.40578800
C	-2.66711600	1.91179900	-0.39487300
C	-3.35782900	0.70549300	-0.48159000
C	-2.62574900	-0.47054300	-0.57777500
C	3.97784800	2.59422100	-0.07322400
C	-3.35347500	3.23637200	-0.28873700
O	-0.20467900	-2.24835100	-3.55457500
O	-2.38800000	-4.06790200	-0.30139400
O	1.79962700	-4.44313200	-0.48166800
O	-4.67146000	3.11203200	-0.28748900
O	5.25499400	2.24661900	-0.03969100
O	-2.76102000	4.28354100	-0.21419400
O	3.56985200	3.72413200	0.02792100
H	2.98639800	-1.95430200	-0.61526700
H	4.62626700	-0.10765500	-0.33233700
H	1.33251400	2.65295500	-0.20765300
H	-0.73455600	2.83917200	-0.33702900
H	-4.44796500	0.67555300	-0.47571700
H	-3.14198200	-1.43148800	-0.65064200
O	0.02714000	-2.09355200	1.45934000
S	-0.55000900	-0.96553300	2.35738500
C	0.75607400	-0.73625900	3.56178400
H	1.60442200	-0.28905600	3.02461600
H	1.02907400	-1.72012500	3.96991300
H	0.40534400	-0.05410500	4.34914200
C	-1.75739100	-1.82185400	3.37116900
H	-2.58102800	-2.11117800	2.70315700
H	-2.12683800	-1.13663200	4.14735400
H	-1.27935200	-2.71061300	3.80810300
C	6.20026400	3.29764600	0.12828600
H	7.19005500	2.82932200	0.13386000
H	6.12635800	4.01821400	-0.69930100

H	6.02506400	3.82557400	1.07709100
C	-5.42788600	4.31419000	-0.19161800
H	-5.20978800	4.97523400	-1.04313200
H	-6.48280500	4.02077800	-0.20193900
H	-5.19085500	4.84535100	0.74177700

Table S63: Coordinates for $Mn^{I}(4dcarbpy)(CO)_3H$

Mn	0.00000900	2.44366000	-0.14872800
N	1.29686200	0.86428000	-0.08107200
C	0.00011800	2.74882900	1.65014800
C	1.26584800	3.63574100	-0.55110100
C	-1.26585600	3.63578300	-0.55089200
N	-1.29686200	0.86430300	-0.08101000
C	2.63434300	0.95282600	-0.05876400
C	3.46539100	-0.15651200	-0.04239200
C	2.88680000	-1.42649900	-0.04610300
C	1.50092100	-1.52537100	-0.06052700
C	0.73162600	-0.36372000	-0.07441900
C	-0.73164600	-0.36370800	-0.07439300
C	-1.50095900	-1.52534700	-0.06049400
C	-2.88683600	-1.42645400	-0.04602900
C	-3.46540600	-0.15645800	-0.04228400
C	-2.63434200	0.95286700	-0.05866300
C	3.68545900	-2.68605800	-0.03204200
C	-3.68551300	-2.68600100	-0.03196200
O	0.00020200	3.00009600	2.77681900
O	-2.08794500	4.38189000	-0.87519500
O	2.08796800	4.38183700	-0.87535000
O	-4.99081300	-2.44879400	-0.01782200
O	4.99076200	-2.44887100	-0.01790300
O	-3.19361100	-3.78777700	-0.03333800
O	3.19353900	-3.78782600	-0.03335400
H	3.06390200	1.95777800	-0.05784400
H	4.54877600	-0.03021000	-0.02616000
H	1.04073700	-2.51564800	-0.05904700
H	-1.04078900	-2.51563000	-0.05904300
H	-4.54878900	-0.03013900	-0.02602100
H	-3.06388800	1.95782400	-0.05771400
H	-0.00003700	2.24240000	-1.74443900
C	-5.84535200	-3.58546300	-0.00285100
H	-5.65919700	-4.19780700	0.89180500
H	-6.87228100	-3.20491700	0.00812000
H	-5.68102900	-4.20504800	-0.89680500
C	5.84528400	-3.58555300	-0.00289200
H	6.87221800	-3.20502200	0.00807900
H	5.65911000	-4.19787000	0.89177800
H	5.68096000	-4.20515900	-0.89683100

Table S64: Coordinates for Mer-$Mn^{I}(4dcarbpy)(CO)_3Br$

Mn	-1.10024300	-1.61530600	0.00007200
N	-1.10280500	0.47179800	-0.00002700
C	-1.24136500	-1.61798400	1.85800000
C	-1.24130900	-1.61817400	-1.85786100
C	-0.96749400	-3.40028100	0.00016700
N	0.87907800	-1.22811300	0.00007300
C	-2.17950200	1.26239700	-0.00006500
C	-2.08817000	2.64873500	-0.00012100
C	-0.82375600	3.23454900	-0.00013800
C	0.29726700	2.41141900	-0.00009900
C	0.12610000	1.02966700	-0.00004300
C	1.24059600	0.07637200	0.00000500
C	2.57754700	0.46464100	-0.00001700
C	3.57264900	-0.50609000	0.00003300
C	3.19456500	-1.84834400	0.00010600
C	1.84323200	-2.15970200	0.00012300
C	-0.61081100	4.71307400	-0.00019900
C	4.99630700	-0.05985900	0.00000100
O	-1.39108800	-1.63963300	2.99241300
O	-0.89121400	-4.55294500	0.00022900
O	-1.39099600	-1.63994400	-2.99227600
O	5.83880800	-1.08404900	0.00007100
O	-1.75580300	5.38113400	-0.00023500
O	5.33071400	1.09925800	-0.00005500
O	0.48014800	5.22754100	-0.00021500
H	-3.14825800	0.75124700	-0.00005000
H	-2.99218300	3.25931500	-0.00015100
H	1.28921800	2.86727600	-0.00011200
H	2.86609700	1.51782700	-0.00007500
H	3.93955800	-2.64514500	0.00014800
H	1.52223500	-3.20410600	0.00017800
Br	-3.64234100	-1.78734200	0.00003600
C	7.22617600	-0.77013800	0.00006600
H	7.76283200	-1.72483100	0.00017900
H	7.49094600	-0.18736300	-0.89448100
H	7.49091100	-0.18716800	0.89449600
C	-1.66696100	6.80095700	-0.00029700
H	-1.13365600	7.15489100	-0.89481700
H	-2.69494000	7.17858200	-0.00031600
H	-1.13365900	7.15497000	0.89419300

Table S65: Coordinates for $Mn^{I}(4dcarbpy)(CO)_2Br(DMSO)$

C	1.19706400	-3.12780900	0.42256100
C	1.54725800	-1.79124300	0.56205600
N	0.64756600	-0.80701800	0.50426900
C	-0.65103700	-1.10282000	0.30411900
C	-1.08164600	-2.41921100	0.15619300
C	-0.14566100	-3.44718700	0.21468800
C	-1.53968700	0.06252800	0.25884000
N	-0.92084800	1.25783900	0.42002000
C	-2.91501500	-0.03228600	0.06906100
C	-3.68493800	1.12543700	0.03691600
C	-1.66952500	2.37032900	0.38667000
C	-3.04302100	2.35435400	0.19886200
Mn	1.07085400	1.20220300	0.58724600
C	1.04634400	1.26491900	2.36313000
O	1.03200400	1.32190700	3.52075700
C	1.33662400	2.95985600	0.50150700
O	1.49671500	4.10731300	0.43359500
H	1.95879100	-3.90713700	0.47389900
H	-2.13262000	-2.66650100	-0.00794600
H	-3.40561300	-0.99968200	-0.05840500
H	-3.60345700	3.29019200	0.17902300
C	5.57027400	1.39942200	0.57670000
S	4.06903700	1.26991400	-0.40357000
C	4.54206000	-0.14410100	-1.41072400
O	3.06974300	0.68043100	0.62635300
H	5.40409100	2.19676900	1.31312500
H	6.40914000	1.66475900	-0.08259300
H	5.74618600	0.43815100	1.08122100
H	5.36405800	0.15084300	-2.07931400
H	3.64909000	-0.41216300	-1.99255900
H	4.84693900	-0.96508200	-0.74444400
C	-0.63013500	-4.84871500	0.04911200
O	0.35913700	-5.73094900	0.11545900
O	-1.78835700	-5.13890300	-0.12544600
C	0.00398500	-7.09969000	-0.03298700
H	-0.46389800	-7.27443800	-1.01314000
H	0.93256500	-7.67478000	0.04855000
H	-0.70010900	-7.40514900	0.75506600
C	-5.15398300	0.99370900	-0.17047200
O	-5.76449900	2.17293400	-0.18567900
O	-5.71833000	-0.06444400	-0.30847600
C	-7.17273300	2.15940200	-0.37911700
H	-7.66942700	1.59609700	0.42473700
H	-7.50111800	3.20422700	-0.36429200

H	-7.42732300	1.69724000	-1.34441500
Br	1.15322800	1.10977600	-1.99351700
H	2.58207700	-1.47275300	0.72061000
H	-1.14399100	3.32013700	0.51472100

Table S67: Coordinates for $[Mn^{I}(4dcarbpy)(CO)_2(DMSO)_2]^+$

C	1.60252800	3.12672200	-0.52751600
C	1.81188300	1.78344000	-0.81627900
N	0.82642500	0.88704600	-0.77866400
C	-0.42218600	1.27391500	-0.45772100
C	-0.71146200	2.60006500	-0.14740400
C	0.31572900	3.53921500	-0.18154900
C	-1.42247600	0.19939500	-0.49600000
N	-0.94847800	-1.01065900	-0.88342200
C	-2.76400300	0.38957700	-0.17907400
C	-3.65095300	-0.67682500	-0.28440500
C	-1.81356200	-2.02822900	-1.00690800
C	-3.16535600	-1.90926300	-0.72267800
Mn	1.04236600	-1.14885900	-0.95852700
C	1.16752100	-1.10627200	-2.73505100
O	1.24468500	-1.08133300	-3.88909500
C	1.14088800	-2.92810000	-0.99981300
O	1.20661800	-4.08602600	-1.02746800
H	2.42941300	3.83720000	-0.56599100
H	-1.71916800	2.92634400	0.11874700
H	-3.13917800	1.35998900	0.15263300
H	-3.82671100	-2.76984700	-0.83148000
C	5.44067100	-1.62618600	-0.02138300
S	3.74448200	-1.46810500	0.54050600
C	3.95894600	-0.08296200	1.66683400
O	3.05713900	-0.85004300	-0.70815700
H	5.45366500	-2.41356600	-0.78645500
H	6.07528600	-1.91552300	0.82826100
H	5.75840500	-0.66444100	-0.44957700
H	4.55340600	-0.41488200	2.53070400
H	2.94711900	0.20469700	1.98334000
H	4.45926200	0.73865000	1.13266900
C	1.19204700	-3.07050000	2.77078600
S	0.07371000	-1.91147300	1.97456900
C	-0.28681800	-0.85036300	3.37303000
O	1.01277800	-1.01767300	1.11763000
H	1.58944600	-3.72605000	1.98310900
H	0.62529800	-3.66568700	3.50127000
H	2.00213600	-2.50551800	3.25638100
H	-0.84522000	-1.42491300	4.12564200
H	-0.90394400	-0.02414000	2.99390500
H	0.66396100	-0.47271400	3.77769000
C	-0.01799600	4.95526600	0.15667100
O	1.04110100	5.74951100	0.07969000
O	-1.12530900	5.32326900	0.46269500

C	0.83204900	7.12370800	0.38179300
H	0.46706800	7.24196200	1.41266900
H	1.80124100	7.62038300	0.26593400
H	0.09649400	7.56364100	-0.30764200
C	-5.08023700	-0.44490600	0.07253800
O	-5.81596200	-1.53784100	-0.08226400
O	-5.50770200	0.61625100	0.45611800
C	-7.19931600	-1.42580600	0.22858300
H	-7.67951900	-0.67038800	-0.41074400
H	-7.64197400	-2.41063700	0.04522100
H	-7.33756300	-1.13899900	1.28146000
H	2.80186500	1.39086800	-1.06795500
H	-1.40079700	-2.98668700	-1.33312900

5.2.5 Coordinates for extra species

Table S69: Coordinates for DMSO

C	1.35336400	-0.79633400	0.18925600
S	0.00000000	0.22443400	-0.45477700
C	-1.35336700	-0.79632900	0.18925700
O	0.00000400	1.47902800	0.39080000
H	2.28764000	-0.27124400	-0.05269800
H	1.34360200	-1.78696600	-0.28854900
H	1.23465800	-0.87539300	1.28072100
H	-1.34361500	-1.78695400	-0.28856200
H	-2.28763900	-0.27122900	-0.05268600
H	-1.23465500	-0.87540400	1.28072000

Table S70: Coordinates for BNAH

H	-4.97899600	-1.42343300	1.68271500
C	-4.18647300	-1.00039100	1.05870000
C	-4.50147800	-0.37868700	-0.15019600
H	-5.54327800	-0.31095700	-0.47725300
C	-3.49145000	0.16402100	-0.93931200
H	-3.74137700	0.65868500	-1.88480700
C	-2.15282100	0.08840600	-0.53896600
C	-1.08048400	0.65450900	-1.43882400
C	-1.84529300	-0.53202700	0.67506400
H	-0.80455100	-0.59067400	1.01188000
C	-2.85717300	-1.07287800	1.46855300
H	-2.60142500	-1.55369300	2.41738000
N	0.18533500	0.89459200	-0.78402700
C	1.18007500	-0.04111700	-0.79520100
C	0.30869200	1.96501200	0.10676600
C	2.30178700	0.03408800	-0.03859300
C	1.37562200	2.12885000	0.89892900
C	2.54274700	1.18465400	0.91002700
C	3.27856400	-1.06227200	-0.19650100
O	3.11405600	-2.01292000	-0.95921900
N	4.39703900	-0.96184300	0.57001700
H	4.57791700	-0.18917800	1.19724000
H	5.09821500	-1.68949900	0.49121100
H	3.47550300	1.73018700	0.64717600
H	2.72397400	0.81757100	1.94321000
H	1.40053500	3.00113300	1.55779400
H	-0.53432900	2.66308300	0.10566300
H	1.04148800	-0.89578600	-1.46640000
H	-1.43835900	1.59591000	-1.89272000
H	-0.89826800	-0.03224800	-2.28395100

Table S71: Coordinates for $BNAH^{\bullet+}$

H	5.13538600	-0.98550200	-1.73045800
C	4.30135100	-0.74872100	-1.06400300
C	4.37973300	0.35132800	-0.21321100
H	5.27399000	0.98035300	-0.20865700
C	3.31547300	0.65412500	0.63493800
H	3.37592100	1.51850400	1.30495600
C	2.17148300	-0.14687400	0.63993200
C	1.01486700	0.17863400	1.54405100
C	2.09836600	-1.25168300	-0.21611100
H	1.20478300	-1.88631700	-0.21516900
C	3.15895200	-1.55054000	-1.06508000
H	3.09665900	-2.41665900	-1.72944500
N	-0.17141700	0.58488000	0.76700000
C	-1.26562500	-0.21059200	0.71678700
C	-0.11696600	1.76336800	0.05975100
C	-2.36370200	0.11027200	-0.03789400
C	-1.15925000	2.16601900	-0.70692800
C	-2.39170600	1.36266000	-0.82882600
C	-3.49756500	-0.87131000	0.00443500
O	-3.41372200	-1.88214500	0.68448400
N	-4.57497900	-0.56156600	-0.74022600
H	-4.65919200	0.27382300	-1.30614800
H	-5.36012600	-1.20519500	-0.73310600
H	-3.26900500	1.98755400	-0.54622100
H	-2.58878900	1.14975300	-1.90361400
H	-1.08383700	3.11102800	-1.24843200
H	0.80801500	2.33539400	0.15952000
H	-1.25198800	-1.13232700	1.30471600
H	1.26391700	1.00724600	2.22496600
H	0.71633500	-0.68381400	2.15676200

Table S72: Coordinates for BNA$^{\bullet}$

H	4.99731200	-1.46958100	-1.60316500
C	4.19357300	-1.03692100	-1.00048700
C	4.49300800	-0.32664700	0.16213900
H	5.53360400	-0.19850600	0.47444200
C	3.46823600	0.22878800	0.92376000
H	3.70557800	0.79371500	1.83223700
C	2.13156600	0.07567200	0.54151700
C	1.04178100	0.64998900	1.41499900
C	1.83913900	-0.63334800	-0.62721900
H	0.79885400	-0.75192000	-0.95000100
C	2.86499000	-1.18595000	-1.39313100
H	2.62163700	-1.73638100	-2.30667200
N	-0.20331200	0.88512200	0.71888500
C	-1.23815300	0.00014600	0.80030300
C	-0.26927800	1.93773800	-0.21266800
C	-2.35514200	0.09613500	0.01643700
C	-1.37751800	2.07299600	-1.01157400
C	-2.45426700	1.17657700	-0.93663600
C	-3.38664800	-0.95384400	0.21472400
O	-3.35947900	-1.73971300	1.15727400
N	-4.37538400	-0.98718400	-0.71509100
H	-4.31001100	-0.48066600	-1.58923900
H	-5.05017800	-1.74198000	-0.65657900
H	-3.34659000	1.33332700	-1.54628500
H	-1.40741000	2.91308600	-1.71125400
H	0.59174800	2.60875700	-0.23296400
H	-1.13923600	-0.81255300	1.52646200
H	1.38923000	1.59701500	1.86616100
H	0.83445000	-0.03015700	2.25911100

Table S73: Coordinates for 4,4'-BNA_2

O	-0.53906300	3.89086400	-1.10369100
C	-0.86576800	2.83002400	-0.57355000
N	-2.16821500	2.44617900	-0.52786900
C	0.11697000	1.93426100	0.07688200
C	1.39100000	2.39262500	0.19002800
N	2.32016200	1.76685300	0.97521900
C	1.87372900	0.81672000	1.89436700
C	0.65095600	0.27766100	1.79394500
C	-0.16653000	0.52849900	0.55612000
C	0.16653000	-0.52849900	-0.55612000
C	-0.65095600	-0.27766100	-1.79394500
C	-1.87372900	-0.81672000	-1.89436700
N	-2.32016200	-1.76685300	-0.97521900
C	-1.39100000	-2.39262500	-0.19002800
C	-0.11697000	-1.93426100	-0.07688200
C	0.86576800	-2.83002400	0.57355000
O	0.53906300	-3.89086400	1.10369100
N	2.16821500	-2.44617900	0.52786900
C	-3.74910400	-1.96630500	-0.80636200
C	-4.37812500	-0.76819200	-0.14062600
C	-5.27533900	0.05055700	-0.82895700
C	-5.78993800	1.20135900	-0.22948400
C	-5.40243300	1.54864000	1.06248100
C	-4.50338200	0.73732100	1.75830600
C	-3.99700400	-0.41349000	1.16042800
C	3.74910400	1.96630500	0.80636200
C	4.37812500	0.76819200	0.14062600
C	3.99700400	0.41349000	-1.16042800
C	4.50338200	-0.73732100	-1.75830600
C	5.40243300	-1.54864000	-1.06248100
C	5.78993800	-1.20135900	0.22948400
C	5.27533900	-0.05055700	0.82895700
H	-2.85142800	3.03924300	-0.98520400
H	-2.47929900	1.55242300	-0.16559300
H	1.70162500	3.32797000	-0.28933300
H	2.57209600	0.59550700	2.70931100
H	0.28394800	-0.39208000	2.57687200
H	-1.23680800	0.39209700	0.80116900
H	1.23680800	-0.39209700	-0.80116900
H	-0.28394800	0.39208000	-2.57687200
H	-2.57209600	-0.59550700	-2.70931100
H	-1.70162500	-3.32797000	0.28933300
H	2.47929900	-1.55242300	0.16559300
H	2.85142800	-3.03924300	0.98520400

H	-3.89377500	-2.87878200	-0.20516500
H	-4.21656400	-2.14246800	-1.79081900
H	-5.57044600	-0.21435200	-1.85041100
H	-6.49185800	1.83386600	-0.78044700
H	-5.79916500	2.45396100	1.53050900
H	-4.19659900	1.00373400	2.77385800
H	-3.29172100	-1.05022000	1.70853300
H	4.21656400	2.14246800	1.79081900
H	3.89377500	2.87878200	0.20516500
H	3.29172100	1.05022000	-1.70853300
H	4.19659900	-1.00373400	-2.77385800
H	5.79916500	-2.45396100	-1.53050900
H	6.49185800	-1.83386600	0.78044700
H	5.57044600	0.21435200	1.85041100

Table S75: Coordinates for BNA^+

H	5.16692600	-0.99812700	-1.67556900
C	4.32013100	-0.75768700	-1.02674500
C	4.38675100	0.33999800	-0.17235900
H	5.28416100	0.96398900	-0.14724200
C	3.30594700	0.64734700	0.65313700
H	3.35692800	1.50984100	1.32635300
C	2.15679300	-0.14610200	0.63111500
C	0.98886600	0.18441300	1.51349500
C	2.09692900	-1.25026900	-0.22689300
H	1.20003200	-1.87996700	-0.24559200
C	3.17330800	-1.55297300	-1.05398300
H	3.12062400	-2.41743800	-1.72130600
N	-0.20099100	0.58823300	0.71591400
C	-1.30047000	-0.17916700	0.70538100
C	-0.13674100	1.72596600	-0.01044100
C	-2.40354600	0.15761000	-0.06608200
C	-1.20921800	2.12779300	-0.77679600
C	-2.35598200	1.33546300	-0.81004700
C	-3.57989800	-0.78108400	0.02171400
O	-3.64107300	-1.58710000	0.93373900
N	-4.51238000	-0.65671700	-0.93842500
H	-4.39137300	-0.08886500	-1.76823000
H	-5.30407800	-1.29220000	-0.91606500
H	-3.21068800	1.66169100	-1.40789200
H	-1.14570600	3.05711100	-1.34333700
H	0.79737100	2.28915000	0.05174500
H	-1.30627300	-1.07807600	1.32731100
H	1.22090900	1.01580400	2.19479200
H	0.67612200	-0.67456400	2.12371700

Table S76: Coordinates for H_2

H	0.00000000	0.00000000	0.37905800
H	0.00000000	0.00000000	-0.37905800

Table S77: Coordinates for CO_2

C	0.00000000	0.00000000	0.00000000
O	0.00000000	0.00000000	1.15864200
O	0.00000000	0.00000000	-1.15864200

Table S78: Coordinates for HCO_2^-

C	0.00000000	0.00000000	0.31687400
O	0.00000000	1.12655400	-0.21056200
O	0.00000000	-1.12655400	-0.21056200
H	0.00000000	0.00000000	1.46774500

References

(1) Staal, L.; Oskam, A.; Vrieze, K. The Syntheses and Coordination Properties of $M(CO)_3X(DAB)$ (M=Mn, Re; X= Cl, Br, I; DAB= 1,4-Diazabutadiene). *J. Organomet. Chem.* **1979**, *170*, 235–245.

(2) Bourrez, M.; Molton, F.; Chardon-Noblat, S.; Deronzier, A. [Mn (bipyridyl)$(CO)_3$Br]: An Abundant Metal Carbonyl Complex as an Efficient Electrocatalyst for CO_2 Reduction. *Angew. Chem., Int. Ed.* **2011**, *50*, 9903–9906.

(3) Pordel, S.; Schrage, B. R.; Ziegler, C. J.; White, J. K. Impact of steric bulk on photoinduced ligand exchange reactions in Mn(I) photoCORMs. *Inorganica Chim. Acta* **2020**, *511*, 119845.

(4) Schmittel, M.; Ganz, A.; Schenk, W. A.; Hagel, M. Synthesis and Coordination Properties of 6,6'-Dimesityl-2,2'-bipyridine. *Zeitschrift für Naturforschung B* **1999**, *54*, 559–564.

(5) Sampson, M. D.; Nguyen, A. D.; Grice, K. A.; Moore, C. E.; Rheingold, A. L.; Kubiak, C. P. Manganese Catalysts with Bulky Bipyridine Ligands for the Electrocatalytic Reduction of Carbon Dioxide: Eliminating Dimerization and Altering Catalysis. *J. Am. Chem. Soc.* **2014**, *136*, 5460–5471.

(6) Farrell, K. M.; Ostrander, J. S.; Jones, A. C.; Yakami, B. R.; Dicke, S. S.; Middleton, C. T.; Hamm, P.; Zanni, M. T. Shot-to-Shot 2D IR Spectroscopy at 100 kHz Using a Yb Laser and Custom-Designed Electronics. *Opt. Express* **2020**, *28*, 33584–33602.

(7) Buhrke, D.; Ruf, J.; Heckmeier, P.; Hamm, P. A Stop-Flow Sample Delivery System for Transient Spectroscopy. *Rev. Sci. Instrum.* **2021**, *92*.

(8) Pordel, S.; White, J. K. Impact of Mn(I) photoCORM ligand set on photochemical intermediate formation during visible light-activated CO release. *Inorganica Chim. Acta* **2020**, *500*, 119206.

(9) Lorenz-Fonfría, V. A.; Kandori, H. Transformation of Time-Resolved Spectra to Lifetime-Resolved Spectra by Maximum Entropy Inversion of the Laplace Transform. *Appl. Spectrosc.* **2006**, *60*, 407–417.

(10) Hobson, M.; Lasenby, A. The Entropic Prior for Distributions with Positive and Negative Values. *Mon. Not. R. Astron. Soc.* **1998**, *298*, 905–908.

(11) Lórenz-Fonfría, V. A.; Kandori, H. Practical Aspects of the Maximum Entropy Inversion of the Laplace Transform for the Quantitative Analysis of Multi-Exponential Data. *Appl. Spectrosc.* **2007**, *61*, 74–84.

(12) Anderson, P. A.; Deacon, G. B.; Haarmann, K. H.; Keene, F. R.; Meyer, T. J.; Reitsma, D. A.; Skelton, B. W.; Strouse, G. F.; Thomas, N. C. Designed Synthesis of Mononuclear Tris(heteroleptic) Ruthenium Complexes Containing Bidentate Polypyridyl Ligands. *Inorg. Chem.* **1995**, *34*, 6145–6157.

(13) Beckwith, J. S.; Rumble, C. A.; Vauthey, E. Data Analysis in Transient Electronic Spectroscopy–An Experimentalist's View. *International Reviews in Physical Chemistry* **2020**, *39*, 135–216.

(14) Buhrke, D.; Oppelt, K. T.; Heckmeier, P. J.; Fernandez-Teran, R.; Hamm, P. Nanosecond Protein Dynamics in A Red/Green Cyanobacteriochrome Revealed by Transient IR Spectroscopy. *J. Chem. Phys.* **2020**, *153*, 245101.

(15) Garcia Alonso, F. J.; Llamazares, A.; Riera, V.; Vivanco, M.; Garcia Granda, S.; Diaz, M. R. Effect of an nitrogen-nitrogen chelate ligand on the insertion reactions of carbon monoxide into a manganese-alkyl bond. *Organometallics* **1992**, *11*, 2826–2832.

(16) Dey, S.; Masero, F.; Brack, E.; Fontecave, M.; Mougel, V. Electrocatalytic metal hydride generation using CPET mediators. *Nature* **2022**, *607*, 499–506.

(17) Frisch, M. J. et al. Gaussian~16 Revision A.03. 2016; Gaussian Inc. Wallingford CT.

(18) Zhao, Y.; Truhlar, D. G. The M06 suite of density functionals for main group thermochemistry, thermochemical kinetics, noncovalent interactions, excited states, and transition elements: two new functionals and systematic testing of four M06-class functionals and 12 other functionals. *Theor. Chem. Acc.* **2008**, *120*, 215–241.

(19) Grimme, S.; Antony, J.; Ehrlich, S.; Krieg, H. A consistent and accurate *ab initio* parametrization of density functional dispersion correction (DFT-D) for the 94 elements H-Pu. *J. Chem. Phys.* **2010**, *132*.

(20) Weigend, F.; Ahlrichs, R. Balanced Basis Sets of Split Valence, Triple Zeta Valence and Quadruple Zeta Valence Quality for H to Rn: Design and Assessment of Accuracy. *PCCP* **2005**, *7*, 3297–3305.

(21) Li, H.; Pomelli, C. S.; Jensen, J. H. Continuum solvation of large molecules described by QM/MM: a semi-iterative implementation of the PCM/EFP interface. *Theor. Chem. Acc.* **2003**, *109*, 71–84.

(22) Li, H.; Jensen, J. H. Improving the efficiency and convergence of geometry optimization with the polarizable continuum model: New energy gradients and molecular surface tessellation. *J. Comput. Chem.* **2004**, *25*, 1449–1462.

(23) Ochterski, J. W. Thermochemistry in Gaussian. *Gaussian Inc* **2000**, *1*.

(24) Waldie, K. M.; Ostericher, A. L.; Reineke, M. H.; Sasayama, A. F.; Kubiak, C. P. Hydricity of Transition-Metal Hydrides: Thermodynamic Considerations for CO_2 Reduction. *ACS Catal.* **2018**, *8*, 1313–1324.
